# Barriers and facilitators to the implementation and adoption of computerised clinical decision support systems: an overview of reviews

**DOI:** 10.1186/s13643-026-03200-2

**Published:** 2026-05-13

**Authors:** Anna Katharina Böhm-Hustede, Johanna Sophie Lubasch, Anna Thalea Hoogestraat, Eike Buhr, Antje Wulff

**Affiliations:** 1 Big Data in Medicine, Carl von Ossietzky Universität Oldenburg, Ammerländer Heerstraße 114-118, 26129 Oldenburg, Germany; 2 Oldenburg Research Network Emergency- and Intensive Care Medicine (OFNI), Carl von Ossietzky Universität Oldenburg, Ammerländer Heerstraße 114-118, 26129 Oldenburg, Germany; 3 Ethics in Medicine, Carl von Ossietzky Universität Oldenburg, Ammerländer Heerstraße 114-118, 26129 Oldenburg, Germany

**Keywords:** Systematic review, Overview of reviews, Umbrella review, Clinical decision support systems, Barriers, Facilitators, Implementation, Evaluation, Human factors, Contextual factors

## Abstract

**Background:**

The use of computerised clinical decision support systems (CDSSs) holds considerable potential in enhancing healthcare delivery by improving patient safety, practitioner performance, and patient outcomes. However, despite the numerous reported advantages of CDSSs, their adoption remains restricted, thereby compromising the full realisation of their potential. To enable a profound evaluation and successful implementation of these systems, it is imperative to identify the reasons for their limited uptake. The objective of this study is to provide a comprehensive overview of the barriers and facilitators to the implementation and adoption of decision support systems across healthcare settings.

**Methods:**

This study was reported in accordance with the Preferred Reporting Items for Overviews of Reviews (PRIOR) statement. A systematic search was conducted in the databases PubMed, IEEE Xplore, Scopus, and Web of Science from inception to 15 October 2024, targeting at review articles of primary studies focusing on the identification and reporting of barriers and facilitators to the implementation and adoption of CDSSs across healthcare settings. The risk of bias of the included reviews was assessed with the Risk of Bias in Systematic Reviews (ROBIS) tool, and the primary study overlap was calculated using the corrected covered area method. All data on barriers and facilitators to the implementation and adoption of CDSSs were extracted from the included reviews, synthesised through a mapping of identical factors, and ranked according to their frequency of occurrence.

**Results:**

Of the 1640 records retrieved through database searches, 30 reviews were included in this overview, which summarised the findings of 721 unique primary studies. A total of 101 distinct factors influencing the implementation and adoption of CDSSs were identified and could be categorised into the overarching categories of human, technology-related, and contextual factors. The following six factors were reported most frequently: usability, usefulness/perceived benefits, organisational readiness, training, trust, and workflow integration.

**Conclusions:**

The findings of this study highlight the diversity of factors that influence a successful implementation of CDSSs and emphasise the need for their comprehensive evaluation that goes beyond the assessment of general performance aspects but takes into account human and contextual factors.

**Systematic review registration:**

PROSPERO CRD42024507614.

## Background

Computerised clinical decision support systems (CDSSs) are defined as digital tools intended to aid medical professionals in clinical decision-making by integrating patient-specific data, medical knowledge, and decision support algorithms [1]. CDSSs combine patient information with targeted clinical knowledge, grounded in evidence-based guidelines, best practices, and clinical expertise, to provide specific recommendations and alerts to clinicians at the point of care. These systems offer a multitude of functionalities, encompassing such features as personalised medication dosing, diagnostic algorithms, alerts for potential adverse events, or support for clinical workflows.

CDSSs manifest in a variety of forms, types, and functions, but they can generally be divided into two main categories: knowledge-based systems and non-knowledge-based (data-driven) systems. Knowledge-based systems typically employ rule-based reasoning, with medical knowledge being captured in the form of conditional statements (IF-THEN). The system then evaluates the rule against patient-specific data, subsequently generating a respective action or output [2]. In non-knowledge-based systems, artificial intelligence (AI) and machine learning (ML) are utilised to gain novel insights from clinical data and employ it to provide clinical recommendations [1].

The potential of CDSSs to improve and progress healthcare is multifaceted. These systems can enhance patient safety through increased medication safety [3] or by means of reminder functions for specific medical events [1]. In addition, they can exert a positive impact on practitioner performance and patient medical outcome [4], have the ability to improve diagnostic accuracy [5, 6], foster adherence to clinical guidelines [7], and generally elevate clinical efficiency [8]. From an economic perspective, CDSSs can induce considerable cost-savings [9].

Notwithstanding the numerous advantages and the considerable potential of CDSSs, the implementation and adoption of available CDSSs remains rather restricted [10–12]. Kouri et al. [13] conducted a systematic review and meta-regression to evaluate the uptake of CDSSs in published studies, finding that only 34.2% of CDSSs were adopted in practice. Due to an expected reporting bias towards higher adoption rates, they assume the actual uptake to be even lower [13]. Kotsis et al. carried out a study on the use of CDSSs among nephrologists and found that 81.2% of the participants rarely or never employed available CDSSs [14]. Given the comparatively limited reporting of CDSS uptake in published studies [13], it is worthwhile considering the broader digital maturity of hospitals as a further approximation. A frequently employed benchmark is the Healthcare Information and Management Systems Society (HIMSS) Electronic Medical Record Adoption Model (EMRAM), which categorises hospitals according to their digital development into stages from 1 to 7 [15]. In this maturity model, advanced CDSS functionalities that provide evidence-based clinical support are covered in stage 6. Despite the relevance of this model, openly accessible, recent and comprehensive data on EMRAM stage assessments are scarce. However, available reports indicate that the majority of hospitals have not yet reached these advanced stages, suggesting that fully implemented CDSSs remain the exception. For instance, an analysis of of 130 National Health Service (NHS) trusts from 2024 resulted in only 13 trusts being assessed as stage 6 or 7 [16]. In 2024, Snowden et al. conducted a cross-sectional observational study on 1026 United States (US) hospitals, resulting in only 15.8% being referred to stage 6 or 7 [17]. In Germany, the recent digital health readiness evaluation of 1584 hospitals was mapped on the EMRAM stages, showing that 56.0% reached stage 0, 35.9% stage 1, 3.3% stage 2, 3.3% stage 3, and only 0.5% and 1.1% stage 4 or 5, respectively [18]. While these reports do not provide a comprehensive overview of specific CDSS implementations or their adoption, they may serve as a useful indicator for assessing the readiness of hospitals to implement advanced CDSSs.

It is evident that the mere availability of a CDSS does not necessarily guarantee its adoption by the intended users [19, 20]. Thus, the full potential of CDSSs to enhance and optimise healthcare delivery remains unexploited. In order to foster the effective and successful implementation of CDSSs, it is crucial to identify and analyse the underlying reasons for the low uptake and adoption of existing systems.

Despite the existence of various literature reviews addressing barriers and facilitators to CDSS implementation and adoption, these are frequently constrained to a particular healthcare setting, condition, population, or CDSS type [21–26]. Furthermore, there is also a variation in CDSS maturity and the digital ecosystem. Consequently, a comprehensive overview encompassing all findings is required. An overview of this kind has the capacity to provide a comprehensive knowledge base concerning the factors that limit or enhance the implementation and adoption of CDSSs across a range of healthcare settings and conditions. This methodological approach facilitates a wide-ranging examination of barriers and facilitators, in addition to a targeted evaluation of impactful factors affecting a specific domain of application.

The objective of this work is to provide a comprehensive and broad overview of the factors that impede or promote the uptake of CDSS in healthcare. Hence, the following research question is addressed: what are the barriers and facilitators to the implementation and adoption of computerised clinical decision support systems across healthcare settings?

## Methods

### Protocol and registration

The protocol for this overview of reviews has been published [27] and registered in advance with the Prospective Register of Systematic Reviews (PROSPERO, registration number: CRD42024507614). This overview of reviews was conducted and reported in accordance with the Preferred Reporting Items for Overviews of Reviews (PRIOR) statement [28] (see Additional file 1 for the populated checklist).

In accordance with the PRIOR statement, the term “overview of reviews” is employed in this work to denote a systematic review of existing review articles. Synonyms for this study type include “umbrella review”, “meta-review”, or “review of systematic reviews”.

### Eligibility criteria

Systematic reviews, scoping reviews, rapid reviews, meta-analyses, and meta-syntheses of primary studies focusing on the identification and reporting of factors that promote or limit the implementation and adoption of CDSSs were eligible for this overview of reviews. With regard to the study type, the eligibility of articles was not dependent on self-identification, but rather determined by methodological characteristics, requiring an explicit reporting of a systematic search strategy and selection process. The inclusion was not limited by the healthcare setting, condition, healthcare professional providing care, or type of patient that motivates the use of a CDSS. However, the targeted user group must be healthcare professionals and not the patient (i.e. patient decision aids are excluded as their supporting features are not immediately directed towards healthcare professionals). Furthermore, review articles that did not concentrate on CDSSs but on a broader area of technologies (e.g. “eHealth”) were excluded from this overview of reviews. All inclusion and exclusion criteria are summarised in Table 1 based on the PICOS framework.

**Table 1:** PICOS criteria for eligible studies

Criteria	Inclusion	Exclusion
Population	The study must be directly related to any healthcare setting; there are no limitations regarding the healthcare sector, medical condition, healthcare professional providing care, or type of patient that motivates the use of a CDSS	Not healthcare related
Intervention	Implementation or evaluation of CDSSs whose support features are directed to any healthcare professional	$\bullet$ Includes patient-facing CDSS
		$\bullet$ Includes CDSS that is not computerised
		$\bullet$ No CDSS
Comparator	No comparator applicable	No comparator applicable
Outcomes	Qualitative or quantitative data on barriers and facilitators to the use and adoption of CDSSs	$\bullet$ Data on barriers to the implementation of specific technologies/algorithms in the development of CDSSs (e.g. the lack of explainability of AI technologies may prevent their use in CDSSs)
		$\bullet$ No focus on barriers and facilitators
Study type	$\bullet$ Systematic review	$\bullet$ Overview of reviews (also known as umbrella review)
	$\bullet$ Scoping review	$\bullet$ Review including not exclusively primary studies, but also other reviews
	$\bullet$ Rapid review	
	$\bullet$ Meta-analysis	
	$\bullet$ Meta-synthesis	

*PICOS* population, interventions, comparators, outcomes, and study types, *CDSS* computerised clinical decision support system

### Information sources and search strategy

Relevant publications were retrieved through comprehensive searches of four electronic bibliographic databases, including PubMed, IEEE Xplore, Scopus, and Web of Science. The search was conducted on 14 December 2023 and updated on 15 October 2024. The searches were not limited by article language, publication date, or publication type.

The search strategy was constructed in accordance with the PICOS framework, addressing systematic literature reviews of primary studies identifying barriers and facilitators to the implementation and adoption of CDSSs across healthcare settings. The search string is composed of four “AND”-concatenated thematic blocks, each comprising multiple “OR”-concatenated search terms targeting the respective eligibility theme. The first thematic block represents the required embedding into a medical context (population), the second block addresses the use of CDSSs (intervention), the third block covers the aspired identification of factors that promote or limit the use and adoption of a CDSS (outcome), and the fourth block defines the search for systematic literature review articles (study type). As the eligibility of studies is not constrained by particular comparators, there are no search terms covering the comparator part of the PICOS framework within the search string. All search terms were restricted to title, abstracts and/or keywords. Where applicable, standard search terms were supplemented by suitable MeSH terms. The following search string was constructed for PubMed:

(clinic*[Title/Abstract] OR medic*[Title/Abstract] OR healthcare[Title/Abstract])

AND

(decision support systems, clinical[MeSH Terms] OR “decision support system*”[Title/Abstract] OR “decision support tool*”[Title/Abstract] OR “computer* decision support”[Title/Abstract] OR “clinical decision support”[Title/Abstract] OR CDSS[Title/Abstract])

AND

(barrier*[Title/Abstract] OR limit*[Title] OR hinder*[Title/Abstract] OR enabler*[Title/Abstract] OR acceptability[Title/Abstract] OR acceptance[Title/Abstract] OR adoption[Title/Abstract] OR uptake[Title/Abstract] OR facilitator*[Title/Abstract] OR attitude to computers[MeSH Terms] OR attitude of health personnel[MeSH Terms] OR human factor*[Title/Abstract] OR contextual factor*[Title/Abstract] OR avoidance[Title/Abstract])

AND

(“systematic review*”[Title/Abstract] OR “scoping review*”[Title/Abstract] OR (review*[Title/Abstract] AND literature[Title/Abstract]) OR meta-analys*[Title/Abstract] OR “PRISMA”)

The complete search strategy for each database is available in the supplementary information (see Additional file 2). Additional publications were identified through a manual search of the reference lists of all included articles.

### Selection process

Following the database searches, all retrieved records were imported into Rayyan [29] in order to manage and monitor the study selection process. After the removal of all duplicates, the titles and abstracts of all identified records were independently screened and assessed for eligibility by four authors (AKBH, JSL, ATH, EB) using the predefined inclusion and exclusion criteria. Subsequently, the full-text articles of potentially eligible records were retrieved and independently assessed for inclusion by the same four reviewers. The initial screening process was over-inclusive. If in doubt, the record was included in the subsequent stage of the full-text analysis. Any disagreements between reviewers regarding the eligibility of an article were resolved by discussion or by consultation with a fifth reviewer (AW).

### Data collection process

A data extraction form was developed using Microsoft Excel and subsequently tested and refined with several included articles. The following information was extracted from each included article by one author (AKBH) and verified by a second author (AW): general information about the publication (authors, title, year of publication, publication medium, country), study type (e.g. systematic review, scoping review), methodological framework or reporting guideline (e.g. PRISMA), number of included articles, publication timeframe of included articles, databases searched, quality assessment method, analysis/synthesis method, type of CDSS, target CDSS user group (e.g. physician, nurse), target healthcare setting (e.g. hospital, primary care), target medical condition/health domain (e.g. cancer, chronic diseases), barriers and facilitators to adoption and implementation of CDSS, and the framework used to map those factors, where applicable. Furthermore, for each included article, all included primary studies were identified and their citations exported into Citavi 7 in order to facilitate the management of primary studies and identify the number of unique primary studies or their overlap. Citavi 7 is a reference management software used in academic fields.

### Risk of bias assessment and overlap management

The risk of bias of eligible articles was evaluated using the Risk of Bias in Systematic Reviews (ROBIS) tool [30] by one author (AKBH) and confirmed by a second author (AW). The ROBIS tool supports a rigorous assessment of the included articles by identifying concerns with the review process in four key domains: study eligibility criteria, identification and selection of studies, data collection and study appraisal, and synthesis and findings. The overall risk of bias of the eligible articles is classified as high, low, or unclear, based on the individual assessment guided by the signalling questions in each domain. ROBIS was applied consistently across all included reviews, irrespective of their review type. It is acknowledged that not all review types routinely include risk of bias assessments of primary studies (e.g. scoping reviews). However, the intention is to account for those that do so, as the risk of bias in reviews that do not consider the risk of bias of their included primary studies is potentially higher.

In the context of overviews of reviews, there is a potential for a primary study to be included in more than one eligible review, resulting in the occurrence of a primary study overlap and the subsequent attribution of undue weight to any overlapping study [31]. In order to address this issue and evaluate the extent of primary study overlap among the included reviews, citation matrices and the corrected covered area (CCA) method were employed. A citation matrix is created by listing all the included reviews as columns and all the included unique primary studies as rows. Subsequently, each review (column) is assigned all of the included primary studies by marking the cells in the corresponding rows. Then, the CCA is calculated using the following formula:

$$\begin{aligned} CCA = \frac{N - r}{rc - r} \end{aligned}$$

where *N* is the number of included primary studies (including double counting; corresponds to the number of marked cells in the citation matrix), *r* is the number of unique primary studies (number of rows), and *c* represents the number of included reviews (number of columns). A CCA value of 0–5% is indicative of slight overlap, 6–10% is considered as a moderate overlap, 11–15% is interpreted as high overlap, and a value above 15% represents a very high overlap [32, 33]. The Graphical Representation of Overlap for OVErviews (GROOVE) tool was used to calculate and visualise the CCA values for the included reviews [34].

### Data synthesis

As part of a thematic analysis, the barriers and facilitators extracted from each included review were analysed, and conceptually overlapping factors were combined in order to determine the unique set of influential factors identified across all included reviews, as well as their frequency of occurrence. For included reviews that did not report their synthesised findings in enough detail but provided the data on extracted factors from their primary studies, this data was used for the mapping.

The categorisation of an identified factor as either a barrier or a facilitator was not a determining factor in the mapping of the set of influencing factors. Rather, it was understood as the characteristics and direction of effect of the respective factor. Thus, for each identified unique factor, both the absolute number of occurrences within all included reviews and the individual direction of impact in a single review were synthesised. Given that each of the included reviews synthesises the findings of several primary studies, the manifestation of an influential factor in a single included review can be either solely a barrier, a facilitator, or both. In the latter case, two types of characteristics can be recognised: either the same factor is listed once as a barrier and once negated as a facilitator (e.g. lack of training (barrier) vs. sufficient training (facilitator)), or they are actually expressing opposing effects (e.g. little professional experience, both as barrier and facilitator). In addition, all identified influencing factors were categorised into broader themes that describe the originating concept or environment of the respective factor. In the context of synthesising the frequency of occurrence of influencing factors, it has to be noted that the frequency does not necessarily equate to the strength of effect.

The synthesis of the data was conducted by one author (AKBH) and subsequently checked by a second author (AW). Following this, the synthesis was then discussed and verified in five interdisciplinary workshops with six experts from multiple domains, including computer science, medical informatics, and psychology. The experts were provided with the synthesised results in the form of a complete mapping of all extracted items in order to perform a plausibility check and interpretive validation and a voting on category fit.

In order to evaluate the impact of reviews assessed to be at high risk of bias on the synthesised results of this overview of reviews and to assess their robustness, a subgroup analysis of the low risk of bias reviews was performed.

## Results

### Study selection

**Fig. 1:**
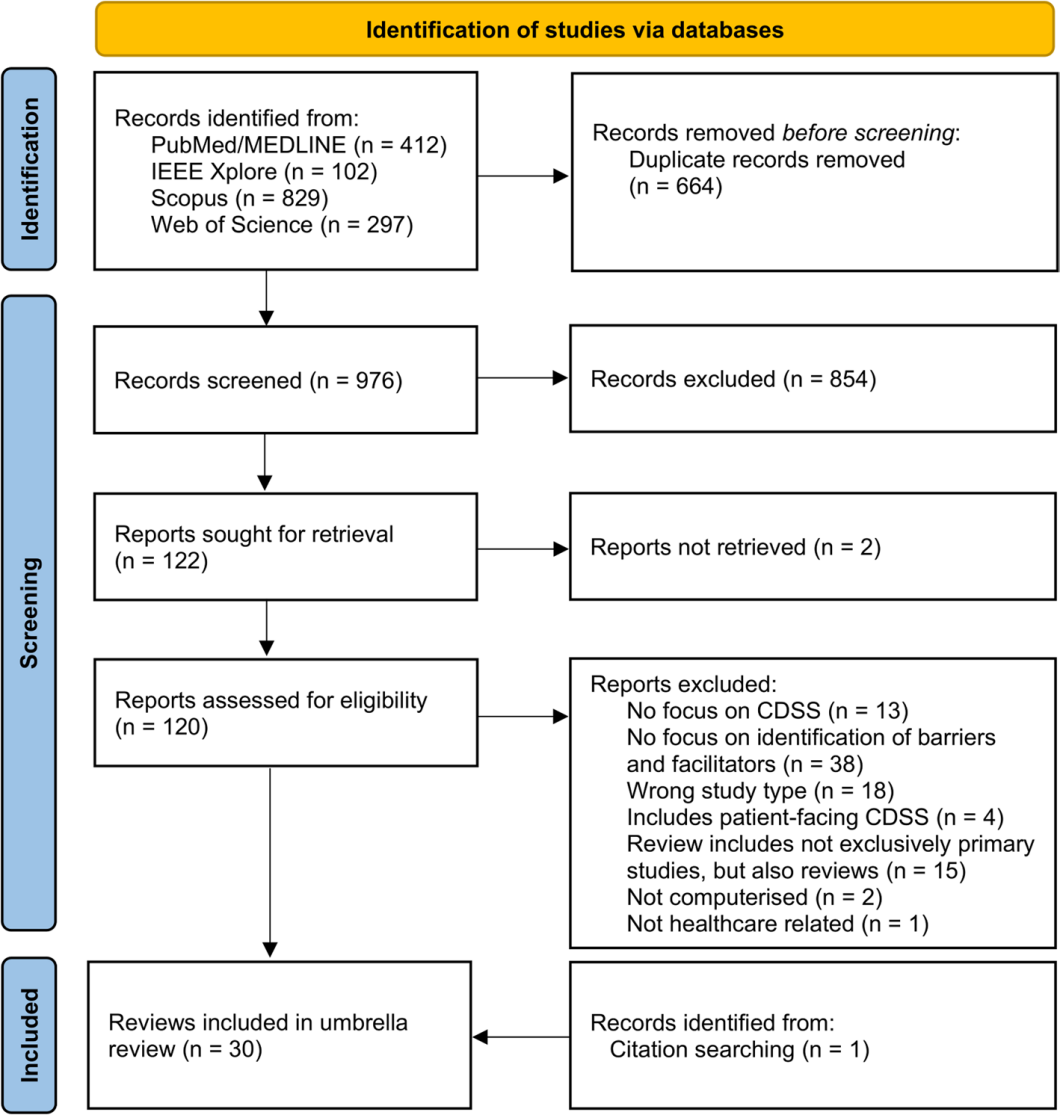
PRISMA flow diagram of study selection process

A total of 1640 records were identified from the initial search in four databases. Following the removal of duplicates, the titles and abstracts of 976 records were screened, resulting in the exclusion of 854 records. Subsequently, 122 reports were sought for retrieval, of which 120 reports were retrieved and assessed for eligibility, leading to the exclusion of 91 articles. A list of these articles, accompanied by the underlying rationales for exclusion, is provided in the supplementary information (see Additional file 3). The remaining 29 articles were included in this overview of reviews, complemented by one additional eligible article identified through citation searching (see Fig. 1 for the PRISMA flow diagram [35] of the study selection process with reasons for exclusion).

### Study characteristics

**Fig. 2:**
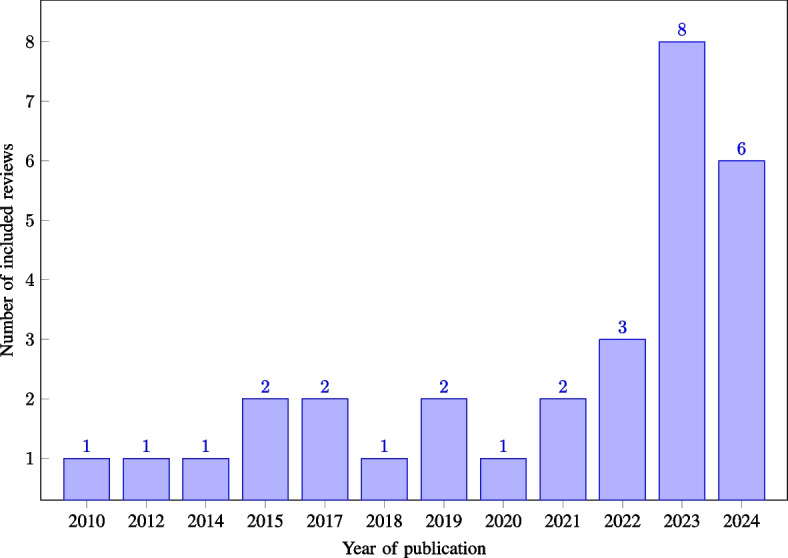
Number of included reviews by publication year ($n=30$)

**Fig. 3:**
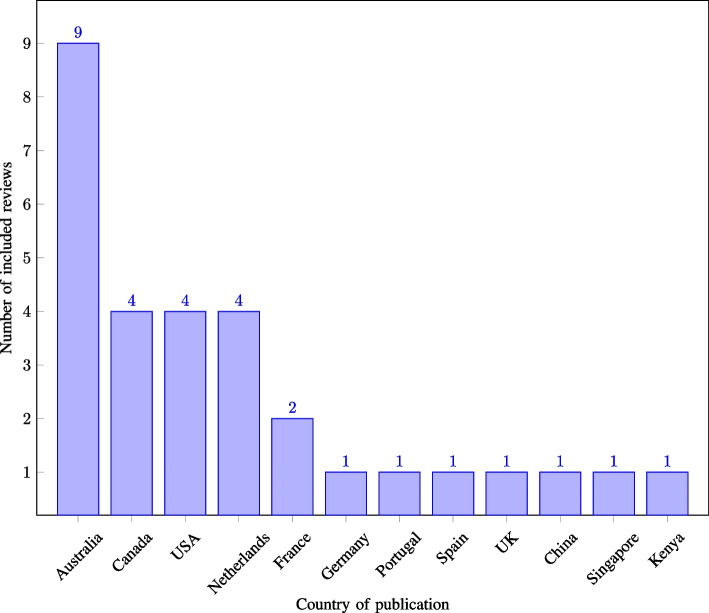
Number of included reviews by country ($n=30$)

**Fig. 4:**
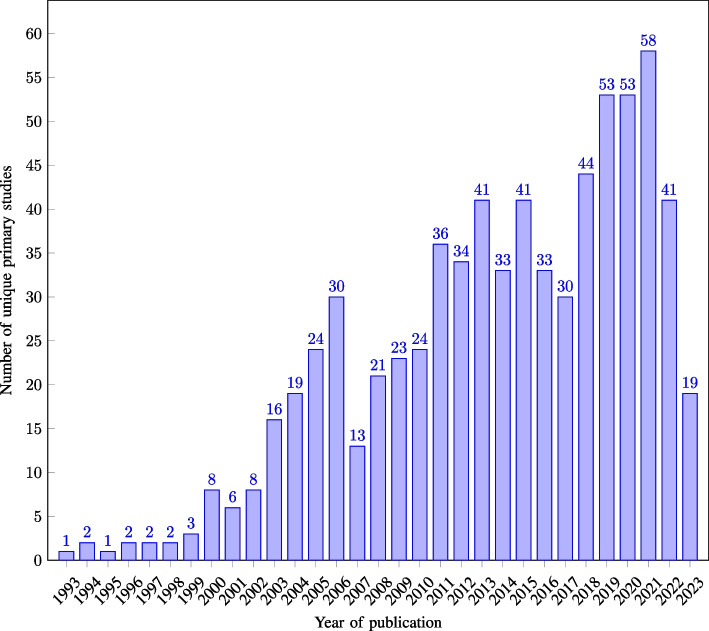
Number of unique primary studies by publication year ($n=721$)

The included reviews were published in 23 distinct scientific journals between 2010 and 2024, with 56.67% of the reviews being published in the years 2022–2024 (see Fig. 2). Of the 30 reviews included, 90.00% had their first author affiliated with a country in Europe, North America, or Australia (see Fig. 3 for the distribution by country). While the first author’s affiliation does not imply direct information regarding the country in which the included primary studies are conducted, it is noteworthy that the interpretation and prioritisation of primary study results by the original authors may be influenced by their respective cultural backgrounds. The 30 reviews included between four and 63 primary studies, with a mean of 28.2 and a median of 26 primary studies. The primary studies included in the reviews were published between 1993 and 2023, with 74.91% of these published from 2010 onwards, and 51.60% between 2015 and 2023 (see Fig. 4 for the distribution by year of publication).

With regard to the CDSS type, 30.00% ($n=9$) of the reviews focused on CDSSs described as based on AI, ML, prediction models, or as data-driven [36–44]. Restrictions to mobile CDSSs [45, 46], electronic health record (EHR) based CDSSs [21, 47], or alerting systems [48, 49] were imposed in 6.67% ($n=2$) of the reviews, respectively. One review each (3.33%) concentrated on CDSSs described as knowledge-based [50], guideline-based [51], computerised physician order entry (CPOE) embedded [52], or as cognitive support technologies [53]. The remaining 36.67% ($n=11$) of the reviews did not describe any restrictions to the type of CDSS under investigation [13, 22–24, 54–60]. As the classification of the CDSS type was based on the descriptions and restrictions of CDSSs considered in the included reviews as provided by the respective authors, the categories are not necessarily mutually exclusive.

The specifications of the targeted user group were given in heterogeneous levels of detail. In 80.00% ($n=24$) of the reviews, the targeted user group was no more restricted or specified than any kind of medical professional (miscellaneous), in some cases generally described as “clinicians” [13, 21, 23, 24, 36, 37, 39–49, 53, 54, 56–60]. In the respective reviews, this resulted in the inclusion of primary studies with varying user groups (e.g. one primary study focusing on nurses and another on physicians). In each of the remaining reviews included in this overview of reviews, the user group was described as physicians [51], nurses [50], general practitioners [55], oncological multidisciplinary teams [38], primary care professionals [22], or prescribers [52], respectively.

In 23.33% of the reviews ($n=7$), the healthcare setting was confined to hospitals [36, 41, 43, 52–54, 60], with two reviews focusing exclusively on emergency departments [36, 53]. A proportion of 16.67% of the reviews ($n=5$) focused on primary care facilities or any outpatient settings [21, 22, 45, 55, 57]. In half of the reviews ($n=15$), the healthcare setting was not restricted [13, 23, 24, 37, 39, 40, 42, 44, 46, 47, 49–51, 58, 59], while 10.00% ($n=3$) did not report on the specific setting [38, 48, 56]. Furthermore, two of the reviews exclusively considered primary studies conducted in resource-constrained settings [45, 47], while the remaining reviews did not impose any restrictions on the resource-related setting of included studies.

With regard to the medical condition or field of support considered, 53.33% ($n=16$) of the reviews did not concentrate on any specific medical condition but examined a broad range of studies [13, 22, 36, 37, 41–45, 51, 53, 54, 56, 58–60]. A total of 16.67% ($n=5$) of the reviews focused on medication-related support [23, 24, 48, 49, 52], while 10.00% ($n=3$) exclusively addressed infectious or chronic diseases [21, 47], with one of these reviews solely focusing on asthma [57]. In 6.67% ($n=2$) of the reviews, the medical condition is restricted to cancer [38, 55]. The remaining reviews are each focused on a specific area, namely pressure ulcer management [50], mental health [39], medical imaging [40], and healthcare emergencies [46]. The characteristics of each included review can be found in Table 2.

**Table 2:** Characteristics of included reviews

Author (Year)	Country (affiliation of first author)	Review type	Number of primary studies	Publication timeframe of primary studies	CDSS type	User group	Healthcare setting	Medical condition, field of support
Abell et al. (2023) [54]	Australia	Scoping review	44	2002–2022	Miscellaneous	Miscellaneous	Hospital	Miscellaneous
Adepoju et al. (2017) [45]	Netherlands	Scoping review	22	2008–2016	Mobile CDSS	Miscellaneous	Primary healthcare facilities (resource constrained in Africa)	Miscellaneous
Araujo et al. (2020) [50]	Portugal	Systematic review	16	1995–2017	Knowledge-based systems	Nurses	Miscellaneous	Pressure ulcer management
Chan et al. (2023) [36]	Singapore	Scoping review	36	2000–2021	Prediction models	Miscellaneous	Hospital (emergency department)	Miscellaneous
Chen et al. (2022) [21]	Australia	Systematic review and meta-aggregation	33	2011–2020	EHR-based CDSS	Miscellaneous	Non-acute setting (e.g. primary care, specialist outpatient care)	Chronic diseases
Chima et al. (2019) [55]	Australia	Systematic review	12	2000–2017	Miscellaneous	General practitioners	Primary care	Cancer
Devaraj et al. (2014) [56]	USA	Systematic review	26	2000–2011	Miscellaneous	Miscellaneous	NR	Miscellaneous
Dingel et al. (2024) [37]	Germany	Systematic review and meta-analysis	17	2019–2023	AI-CDSS	Miscellaneous	Miscellaneous	Miscellaneous
Fernández-Barceló et al. (2023) [48]	Spain	Systematic review	12	2006–2020	Alerts	Miscellaneous	NR	Medication-related
Gao et al. (2022) [57]	USA	Scoping review	35	1999–2022	Miscellaneous	Miscellaneous	Outpatient care	Asthma
Hendriks et al. (2024) [38]	Netherlands	Scoping review	44	2005–2023	Data-driven CDSS	Oncological multidisciplinary teams	NR	Cancer
Higgins et al. (2023) [39]	Australia	Integrative review	4	2020–2021	AI/ML-CDSS	Clinicians	Miscellaneous	Mental health
Hua et al. (2024) [40]	Australia	Scoping review	31	2020–2023	AI-CDSS	Miscellaneous	Miscellaneous	Medical imaging, radiology
Jun at al. (2018) [53]	Canada	Scoping review	24	2006–2017	Point-of-care cognitive support technologies	Miscellaneous	Hospital (emergency department)	Miscellaneous
Kamel Rahimi et al. (2024) [41]	Australia	Systematic review	26	2019–2022	AI-CDSS on EMR data	Miscellaneous	Hospital	Miscellaneous
Kilsdonk et al. (2017) [51]	Netherlands	Systematic review	35	2003–2015	Guideline-based CDSS	Physicians	Miscellaneous	Miscellaneous
Kouri et al. (2022) [13]	Canada	Systematic review and meta-regression	55	1999–2020	Miscellaneous	Miscellaneous	Miscellaneous	Miscellaneous
Liu et al. (2021) [58]	USA	Systematic review and meta-regression	34	2003–2019	Miscellaneous	Miscellaneous	Miscellaneous	Miscellaneous
Marcilly et al. (2015) [49]	France	Systematic review	26	2002–2012	Alerting functions	Miscellaneous	Miscellaneous	Medication-related
Meunier et al. (2023) [22]	France	Systematic review	48	1998–2021	Miscellaneous	Primary care professionals	Primary care	Miscellaneous
Miller et al. (2015) [59]	USA	Meta-synthesis	9	2004–2012	Miscellaneous	Miscellaneous	Miscellaneous	Miscellaneous
Moxey et al. (2010) [24]	Australia	Systematic review	58	1993–2007	Miscellaneous	Miscellaneous	Miscellaneous	Medication-related
Olouch et al. (2012) [47]	Kenya	Systematic review	12	2003–2012	EMR-based CDSS	Miscellaneous	Miscellaneous (resource constrained setting)	Infectious or chronic diseases
Perivolaris et al. (2024) [42]	Canada	Systematic review	34	2006–2022	AI-CDSS	Miscellaneous	Miscellaneous	Miscellaneous
Shakibaei Bonakdeh et al. (2024) [60]	Australia	Systematic review and meta-synthesis	13	2012–2023	Miscellaneous	Miscellaneous	Hospital	Miscellaneous
Tricco et al. (2023) [43]	Canada	Scoping review	21	2009–2021	ML-CDSS	Miscellaneous	Hospital	Miscellaneous
van Dort et al. (2019) [52]	Australia	Systematic review and meta-synthesis	13	2009–2018	CPOE-embedded CDSS	Prescribers	Hospital	Medication-related
Wang et al. (2023) [44]	China	Systematic review	20	2012–2022	AI-CDSS	Miscellaneous	Miscellaneous	Miscellaneous
Westerbeek et al. (2021) [23]	Netherlands	Systematic review	63	1996–2020	Miscellaneous	Miscellaneous	Miscellaneous	Medication-related
Wohlgemut et al. (2023) [46]	UK	Systemtic review and qualitative synthesis	23	2003–2021	Mobile CDSS	Miscellaneous	Miscellaneous	Healthcare emergencies (miscellaneous)

*NR* not reported, *CDSS* computerised clinical decision support system, *AI* artificial intelligence, *ML* machine learning, *EHR* electronic health record, *EMR* electronic medical record, *CPOE* computerised physician order entry

### Primary study overlap

**Fig. 5:**
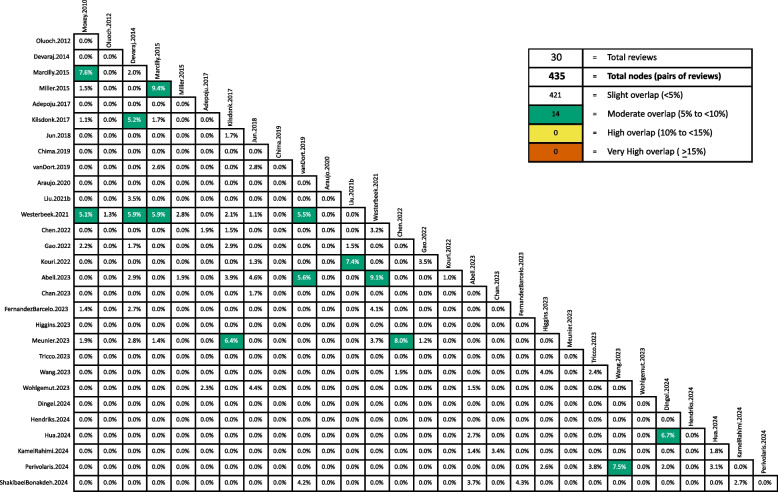
Corrected covered area calculated for each pair of included reviews

A total of 721 unique primary studies were included within the 30 review articles that were included in this overview of reviews. A comprehensive list of these primary studies is provided in the supplementary information (see Additional file 4). The overall overlap of the primary studies is minimal, with a CCA of 0.60%. Even if structural missingness is taken into account to adjust the CCA, the resulting value increases only marginally to 0.72% (see Table 3). Of the 721 unique primary studies, 615 (85.30%) were included in only one of the 30 review articles, and thus did not contribute to any overlap at all. A total of 88 (12.21%) primary studies were included in two review articles, 16 (2.22%) primary studies were included in three reviews, and two (0.28%) primary studies were included in four review articles. The CCA for all possible pairs of included reviews is illustrated in Fig. 5. Of the total of 435 pairs of reviews, 368 (84.60%) exhibited no overlap, 53 (12.18%) pairs showed slight overlap, and 14 (3.22%) pairs of reviews were moderately overlapping.

**Table 3:** Primary study overlap – overall results

Number of columns (number of reviews)	c	30
Number of rows (number of index publications)	r	721
Number of included primary studies (including double counting)	N	847
Covered area	$\frac{N}{rc}$	3.92%
Corrected covered area	$\frac{N-r}{rc-r}$	0.60%
Interpretation of overlap	**Slight overlap**	
Structural zeros	X	3434
Corrected covered area (adjusting by structural zeros)	$\frac{N-r}{rc-r-X}$	0.72%

### Risk of bias

**Fig. 6:**
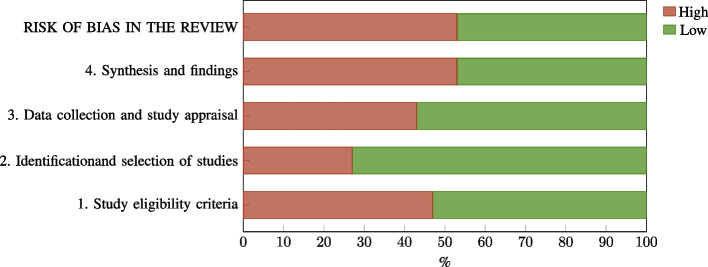
Risk of bias in the included reviews ($n=30$)

The overall risk of bias was rated high in 53.33% ($n=16$) of the included reviews and low in 46.67% ($n=14$). However, the proportion of high risk of bias within the four key domains assessed according to the ROBIS tool varies considerably (see Fig. 6). In domain 4 (synthesis and findings), 53.33% ($n=16$) of the included reviews were judged to be at high risk of bias, followed by 46.67% ($n=14$) of the included reviews being at high risk of bias in domain 1 (study eligibility criteria). A high risk of bias in domain 3 (data collection and study appraisal) was identified in 43.33% ($n=13$) of the included reviews, but only 26.67% ($n=8$) were assessed as being at high risk in domain 2 (identification and selection of studies). A more detailed breakdown of the risk of bias assessment for the individual reviews can be found in Table 4.

Two principal aspects were identified as primarily responsible for the high risk of bias ratings:

1. A language restriction (predominantly to English-language articles) was the primary factor contributing to a high risk of bias rating in domain 1 (when formulated as a restriction in eligibility criteria; $n=14$) and domain 2 (when manifested as a search restriction; $n=8$)).
2. A lacking risk of bias assessment of the included primary studies was the primary factor contributing to a high risk of bias rating in domain 3. This also led to high risk of bias ratings in domain 4, as it is not possible to address any biases of the primary studies in the synthesis if those biases have not been assessed in the first place.

**Table 4:** Results of the risk of bias assessment using the ROBIS tool

	Phase 2				Phase 3
Review	1. Study eligibility criteria	2. Identification and selection of studies	3. Data collection and study appraisal	4. Synthesis and findings	Risk of bias in the review
Abell et al. (2023) [54]	Low	Low	High	High	**High**
Adepoju et al. (2017) [45]	High	Low	High	High	**Low**
Araujo et al. (2020) [50]	High	Low	Low	Low	**Low**
Chan et al. (2023) [36]	High	Low	High	High	**Low**
Chen et al. (2022) [21]	Low	High	Low	Low	**Low**
Chima et al. (2019) [55]	High	Low	Low	Low	**High**
Devaraj et al. (2014) [56]	Low	Low	High	High	**High**
Dingel et al. (2024) [37]	High	Low	High	High	**High**
Fernández-Barceló et al. (2023) [48]	High	Low	Low	High	**High**
Gao et al. (2022) [57]	High	Low	High	High	**High**
Hendriks et al. (2024) [38]	High	Low	High	High	**High**
Higgins et al. (2023) [39]	High	Low	Low	Low	**Low**
Hua et al. (2024) [40]	High	Low	High	High	**High**
Jun at al. (2018) [53]	Low	Low	Low	Low	**Low**
Kamel Rahimi et al. (2024) [41]	High	Low	Low	High	**High**
Kilsdonk et al. (2017) [51]	Low	Low	Low	Low	**Low**
Kouri et al. (2022) [13]	Low	Low	Low	Low	**Low**
Liu et al. (2021) [58]	Low	Low	Low	High	**High**
Marcilly et al. (2015) [49]	Low	High	Low	Low	**Low**
Meunier et al. (2023) [22]	Low	Low	Low	Low	**Low**
Miller et al. (2015) [59]	Low	High	Low	Low	**Low**
Moxey et al. (2010) [24]	Low	High	High	High	**High**
Olouch et al. (2012) [47]	Low	High	High	High	**High**
Perivolaris et al. (2024) [42]	High	Low	Low	Low	**Low**
Shakibaei Bonakdeh et al. (2024) [60]	High	Low	Low	Low	**Low**
Tricco et al. (2023) [43]	Low	High	High	High	**High**
van Dort et al. (2019) [52]	High	Low	Low	Low	**High**
Wang et al. (2023) [44]	Low	High	High	High	**High**
Westerbeek et al. (2021) [23]	Low	Low	Low	Low	**Low**
Wohlgemut et al. (2023) [46]	Low	High	High	High	**High**

### Summary of synthesis of results

All data items on barriers and facilitators to the implementation and adoption of CDSSs that have been extracted from the included reviews were thoroughly analysed with regard to their specific content. Subsequently, a mapping process was employed to identify and align identical or overlapping concepts within the extracted data. A small number of the extracted items were not assignable and and were excluded from the mapping, as their meaning was not clearly discernible ($n=25$, accounting for 1.3% of all extracted items). This synthesis approach resulted in the identification of 101 unique influencing factors across all included reviews. The analysis also yielded a categorisation of the identified factors into three overarching areas: human factors, technology (CDSS)-related factors, and contextual factors. The broad field of human factors encompassed a multitude of facets, including perceptual and attitudinal factors, ethical and legal concerns, knowledge-related factors, skills, social influence, and demographics. The area of technology-related factors mainly concerned factors related to system- or information quality, as well as development aspects. The domain of contextual factors comprised a broader range of variables that arise from the environment and processes within which a system is embedded. These include aspects pertinent to clinical processes, infrastructure, management, implementation strategy, clinical setting, patients, and the external context. While the human factors are directly grounded in the users’ perceptions, attitudes, experiences, skills, or concerns, and the technology-related factors are formed by the specific system and information characteristics, the contextual factors are much more dependent on third instances, local circumstances, or the impact of a system on existing processes.

**Fig. 7:**
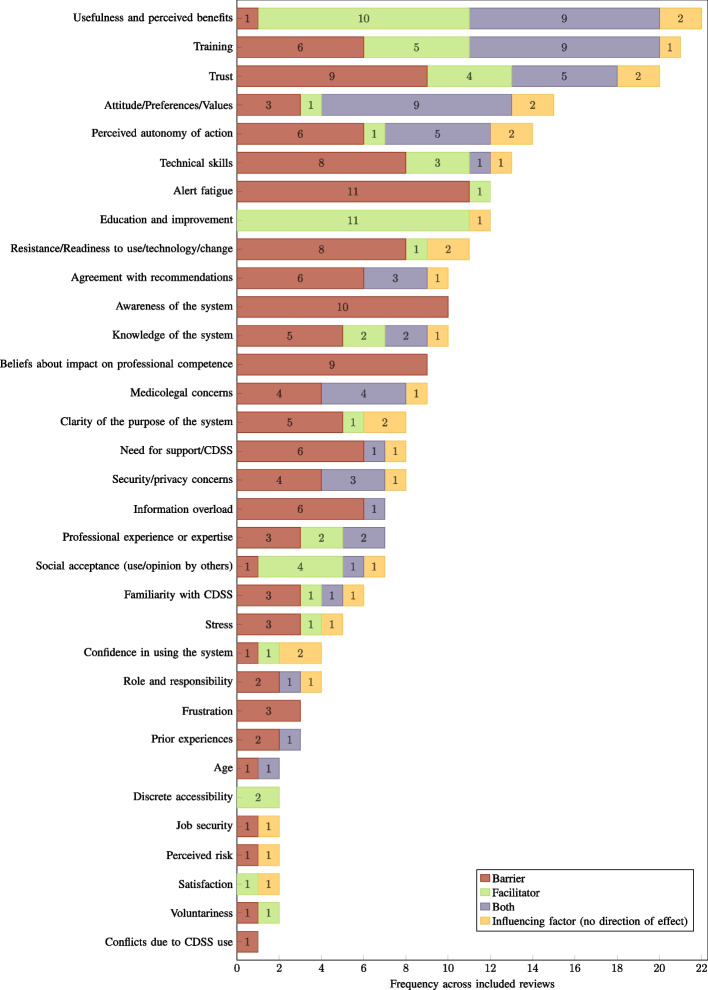
Human factors: frequency across included reviews ($n=30$) by direction of effect. CDSS: computerised clinical decision support system

**Fig. 8:**
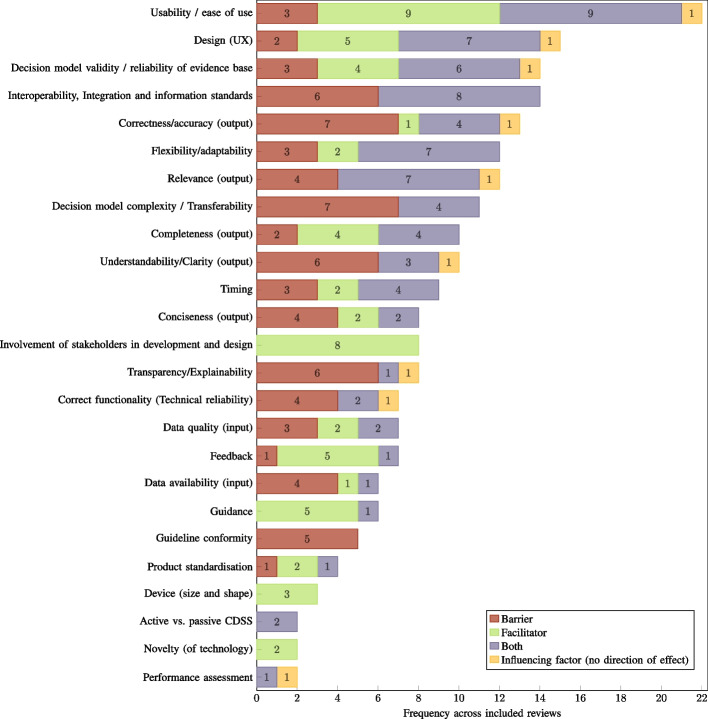
Technology-related factors: frequency across included reviews ($n=30$) by direction of effect. CDSS: computerised clinical decision support system, UX: user experience

**Fig. 9:**
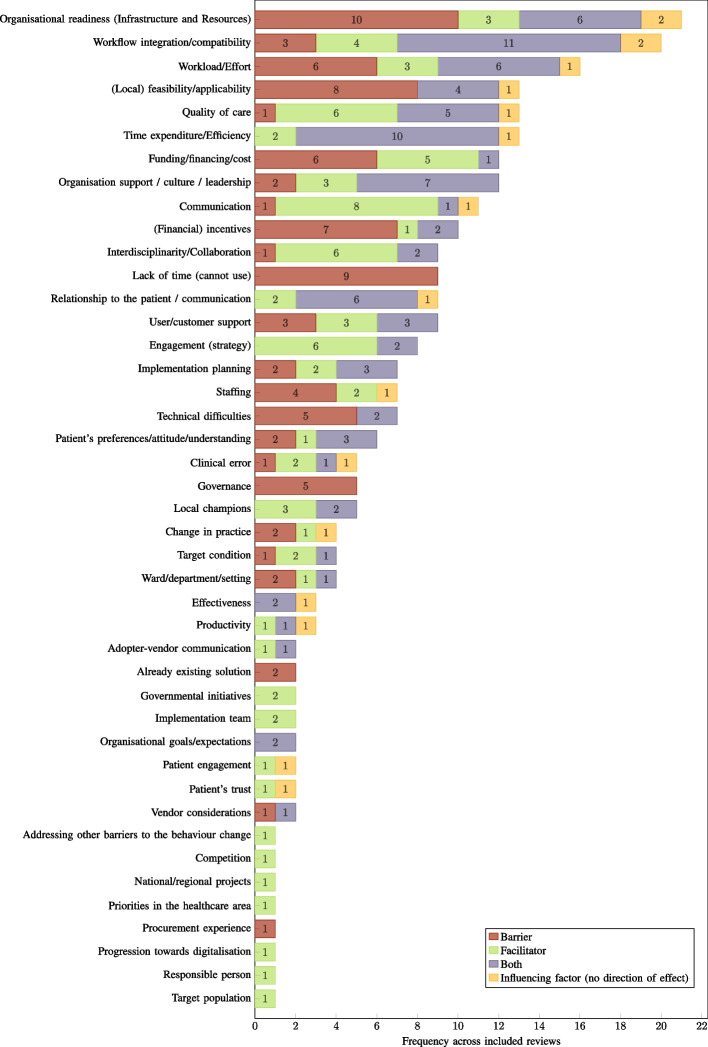
Contextual factors: frequency across included reviews ($n=30$) by direction of effect. CDSS: computerised clinical decision support system

Within the included reviews, an identified influencing factor could be reported as either a facilitator or a barrier, or both. In two reviews, the direction of effect (i.e. barrier or facilitator) was not indicated for the identified factors, which are thus, only reported as generally influencing factors. For each unique factor identified, the absolute number of occurrences within the 30 included reviews was assessed in order to establish a prioritisation of factors. The frequency of occurrence is indicative of the prevalence of reporting, and not necessarily the effect size. The number of occurrences is visualised for each factor in Figs. 7, 8, and 9, with consideration given to the direction of effect (barrier, facilitator, both, or no direction reported).

The most frequently reported influencing factors across all reviews were *Usability* and *Usefulness and perceived benefits*, with a rate of 73.33% ($n=22$). In the case of *Usability*, various usability issues were reported as significant barriers to the implementation and adoption of CDSSs, while aspects highlighting ease of use of a system and the absence of usability flaws were reported as facilitators. In the case of *Usefulness and perceived benefits*, a lack of these factors was the most commonly reported barrier in this category. In addition to general usefulness and perceived benefits acting as facilitators, specific functionalities and features reported as facilitators were also assigned to this category. The second most frequently reported influencing factors were *Organisational readiness* and *Training* with 70.00% ($n=21$) each. The factor *Organisational readiness* is particularly concerned with the infrastructure and resources available at a site that is implementing a CDSS. Problems with infrastructure and resources, or even their absence, are determining barriers to the implementation and adoption of the respective systems. Furthermore, an increase in resource requirements due to a CDSS was also identified as a barrier. In the case of *Training*, the reported barriers manifested analogously in a lack of training, poor training quality, or a delay between training and implementation, while the presence or sufficiency of training was identified as a facilitator. Immediately following, the third most frequently reported influencing factors were *Trust* and *Workflow integration/compatibility*, with 66.67% ($n=20$) each. In the case of *Trust*, in addition to its absence, various reported concerns regarding CDSSs were assigned to this category as barriers. Considering the influential factor *Workflow integration/compatibility*, its absence or workflow interruptions were reported as barriers to the implementation and adoption of CDSSs. Conversely, its presence, an improved workflow, or the feature of not being interruptive to the workflow were identified as facilitators. With regard to the frequency of reporting across all included reviews, the remaining factors ranged between 53.33% and 3.33% ($n=[16,1]$). A detailed overview and mapping of the identified factors to the included reviews can be found in the supplementary information (see Additional file 5).

The categorisation of an identified factor as either a barrier or a facilitator was not a determining aspect with respect to the mapping on the set of influencing factors. Instead, it was interpreted as the direction of effect of the respective factor. Consequently, the influencing factors are named as universally as possible to consider all directions of effect. In many cases, the manifestation of an influencing factor as either a barrier or facilitator was observed as the absence or presence of the factor described (e.g. lack of technical skills (barrier) vs. technical skills (facilitator) for the influencing factor ‘technical skills’). The specific characteristics of all identified influencing factors as barriers and facilitators are described in the supplementary information (see Additional file 6).

While the majority of the individual manifestations of barriers and facilitators of an influencing factor were largely complementary or corresponding with each other, a small number of factors expressed conflicting characteristics. With regard to the factor *Flexibility/adaptability*, conflicting manifestations have been identified in the group of barriers. On the one hand, there is a reported lack of flexibility, adaptability, or customisation; on the other hand, the presence of work around pathways was reported as a barrier, which is attributable to excessive flexibility. Another example is the factor *Active vs. passive CDSS*, where both passive and active characteristics are identified as barriers and facilitators, resulting in conflicting expressions both within and between the categories.

Subgroup analyses regarding healthcare settings and health conditions were deemed to be not feasible due to heterogeneity and inconsistent reporting granularity across included reviews.

In order to consider the relatively high proportion of reviews assessed to be at high risk of bias in the synthesis of results and to evaluate the actual impact of these reviews on the results of this overview of reviews, a subgroup analysis of the low risk of bias reviews was performed. All 101 influencing factors identified within this overview of reviews were also covered and represented by the low-risk subgroup. In a few cases, the relative frequency of reported factors within the subgroup deviates from the ranking of the overall group. However, the differences are mostly minor, and in all three categories (human, technology-related, contextual factors), the most frequently reported factors are the same for the low-risk subgroup and the overall group (see Additional file 7 for a visualisation of the number of occurrences of each factor within the low risk of bias subgroup). This subgroup analysis demonstrates that the results of the included reviews, which were assessed as being at high risk of bias, do not alter or influence the outcomes of this overview of reviews with respect to the identified group of barriers and facilitators.

## Discussion

The objective of this work was to provide a comprehensive overview of the factors that impede or facilitate the implementation and adoption of CDSSs across healthcare settings globally. A total of 30 reviews were included in this overview of reviews, which, in turn, encompassed and synthesised the findings of 721 unique primary studies. Overall, 101 unique influencing factors were identified and categorised into the three overarching categories of human, technology-related, and contextual factors.

Several implementation frameworks have been published in the extant literature, including the Nonadoption, Abandonment, Scale-up, Spread, and Sustainability (NASSS) framework [61], which was developed to assist in predicting the success of technology-supported healthcare programs; the Consolidated Framework For Implementation Research (CFIR) [62, 63] to assess the determinants to implementation effectiveness; and the integrated Promoting Action on Research Implementation in Health Services (i-PARIHS) framework [64]. These frameworks predominantly target a broader scope of healthcare research implementation, and are not necessarily or exclusively related or tailored to CDSSs. Each of the frameworks delineates domains that should be considered when planning or assessing the implementation of innovations. For instance, the NASSS framework defines seven domains that encompass the condition, the technology, the value proposition, the adopter system, the organisation, the wider context, and the embedding and adaptation over time. The CFIR comprises the domains of intervention/innovation, inner and outer setting, the individuals, and the implementation process, while i-PARIHS delineates four constructs: the innovation, recipients, context, and facilitation.

A number of factors identified in this study can also be mapped to domains of established frameworks, e.g. “workflow integration” fits within the NASSS organisation domain, “knowledge of the system” could be attributed to the i-PARIHS recipient construct, and “reliability of evidence base” can be mapped to the CFIR innovation domain. Yet, the added value of this study lies in its comprehensive compilation of factors, tailored to CDSSs, that influence their adoption and use, especially focussing on their specific manifestations as barriers and facilitators. This enables an interpretation of the direction of effect for the factors identified, as the absence of a reported barrier does not necessarily imply facilitation without further confirmation.

The multitude of factors identified underlines that barriers and facilitators can affect the implementation process of a CDSS across different phases, thereby determining the respective implementation success. Factors have an impact on the preliminary stages of planning and procurement, as well as influence the integration and provision of the system. However, in particular the later stages of adoption and sustained use of the CDSSs are subject to a high number of influencing factors.

Furthermore, the mapping of the identified factors to commonly applied CDSS validation measures, such as diagnostic accuracy, clinical relevance, and usability, revealed that only a small subset ($n=12$) is routinely assessed, whereas most factors are not regularly captured. These “performance aspects” of a CDSS are typically evaluated in a validation study by means of standardised metrics, thereby establishing the accuracy and reliability of the system. This focus is likely to be attributed to the requirements for certification or approval processes for medical devices by legislative authorities, e.g. as demanded by the European Union (EU) medical device regulation (MDR) [65]. It is indisputable that this constitutes a relevant stage in the evaluation of a CDSS, as only a valid and accurate system can be safely implemented within any healthcare setting. However, the synthesised findings of this review reflect the importance of further evaluation, taking into account the many additional influencing factors that impair the implementation and adoption of CDSSs, preferably in an early stage, to save resources. It is evident that a theoretically perfect system can only evolve its benefits and supporting features if it is accepted and regularly used. In light of the inherent subjectivity of certain human factors, a standardised evaluation tool enabling the operationalisation of these factors would be highly valuable.

With regard to the potential for a standardised evaluation of the influencing factors that transcend the general performance aspects, the findings reveal that the remaining factors can be categorised into two groups: those of locally independent, more individual perception-oriented and adoption aspects ($n=65$), such as trust or perceived benefit; and those that are strongly dependent on local aspects of the implementing site ($n=24$), such as infrastructure and resources, or organisational goals. While the evaluation of local factors may be challenging in a standardised or generalised manner prior to the concrete implementation on site, due to their strong dependence on the local circumstances and often pre-fixed resource landscapes (e.g. “organisational readiness”), locally independent perception and adoption factors could bear the potential for a standardised assessment in an earlier phase. The large number of influencing factors that could be assigned to the more general and not completely locally dependent aspects underscore the potential of expanding the initial performance-oriented validation of CDSSs with more of the also relevant perception-oriented and adoption factors.

Despite the existence of tools designed to evaluate specific factors (e.g. the System Usability Scale (SUS) [66] for usability, the Charité Alarm Fatigue Questionnaire (CAFQa) [67] for measuring alarm fatigue, or traditional metrics for accuracy, e.g. sensitivity, specificity, area under the curve (AUC), positive predictive value (PPV)), a comprehensive tool that combines the most relevant factors in a low-barrier, efficient manner remains to be developed. The influential factors identified within this overview of reviews have the potential to serve as a cornerstone and a strong foundation for the development of such an evaluation tool that allows a standardised assessment of relevant human and contextual factors affecting the implementation and adoption of CDSSs. In order to achieve this objective, several additional steps are required, including defining measuring methods of factors, their weighting, and validation. The successful development of such an evaluation tool could contribute to a more holistic and use-oriented evaluation of CDSSs. Moreover, it has the potential to enable the generation of specific recommendations and measures that reinforce the facilitating aspects, and reduce the identified barriers. Consequently, this approach may promote an enhanced implementation process and effective utilisation of CDSSs.

### Strengths and limitations

The strength of this study lies in its broad scope, as it is not restricted to a specific clinical setting, medical condition, targeted clinical user group, or type of CDSS. Consequently, a comprehensive overview of barriers and facilitators to the implementation and adoption of CDSSs, irrespective of the aforementioned characteristics, could be generated. A further strength of this overview of reviews lies in its methodological rigour. The selection of studies was subjected to independent screening and study selection by four reviewers, with the objective of minimising any potential error in the selection of studies. The process of data extraction and risk of bias assessment involved two reviewers. The risk of bias of the included reviews was formally assessed using a published and validated tool (ROBIS) and has been addressed in the synthesis of results by demonstrating that the reviews at high risk of bias do not influence the overall results. The primary study overlap was analysed using the CCA method, which demonstrated minimal overlap across reviews. This finding invalidates the common issue of attributing inappropriate weight to overlapping primary studies in overviews of reviews, thereby distorting the results.

However, it must be acknowledged that this study is not without its limitations. A general issue inherent to overview of reviews is that they only consider studies that have been included in existing reviews. Consequently, there is a possibility that relevant individual studies may be omitted from this overview if they have not been incorporated in any systematic review previously. Furthermore, despite the employment of a comprehensive search strategy, it is still conceivable that potentially relevant reviews have not been identified and incorporated into this overview of reviews. However, given that the objective of this overview was to provide a summary of existing data on barriers and facilitators to the implementation and adoption of CDSSs, and not to evaluate any effect sizes, the aforementioned limitations do not compromise the validity of the results. At most, it is possible that they may result in the omission of any influential factor from the overview. A difficulty encountered during the synthesis of results was the heterogeneity in terminology used to describe barriers and facilitators to the implementation and adoption of CDSSs across the included reviews. These differences in phrasing resulted in a complicated mapping of identical or similar factors. In order to minimise any subjectivity that might affect the synthesis and mapping process, the synthesised results were discussed and validated in an interdisciplinary workshop.

## Conclusions

With the increasing development of CDSSs to assist medical professionals in their daily work and enhance healthcare delivery, the need for suitable evaluation and implementation strategies for these systems is becoming more and more apparent. This study provides a comprehensive synthesis of barriers and facilitators specifically relevant to CDSS adoption and use across healthcare settings. The results highlight the importance of extending standard evaluation frameworks beyond technical performance by incorporating human and contextual metrics, which are currently underrepresented.

The findings of this overview of reviews may have meaningful implications for those involved in the implementation processes of CDSSs and researchers exploring human and contextual factors. Future research is needed on evaluation strategies that consider all the relevant facets and layers of aspects influencing a successful implementation of CDSSs. The influential factors identified within this overview of reviews have the potential to inform the development of an evaluation tool that allows for a low-barrier, efficient, but standardised assessment of human and contextual factors affecting the implementation and adoption of CDSSs.

## Supplementary information

Additional file 1. PRIOR checklist.

Additional file 2. Search strategy.

Additional file 3. Excluded articles with reasons.

Additional file 4. List of primary studies.

Additional file 5. Influencing factors: Mapping to the included reviews.

Additional file 6. Influencing factors: Manifestations as barriers and facilitators.

Additional file 7. Frequency of occurrence of influential factors (subgroup: low risk of bias).

## Data Availability

All data generated or analysed during this study are included in this published article and its supplementary information files.

## Abbreviations

AI: Artificial intelligence
AUC: Area under the curve
CAFQa: Charité Alarm Fatigue Questionnaire
CCA: Corrected covered area
CDSS: Computerised clinical decision support system
CPOE: Computerised Physician Order Entry
EHR: Electronic Health Record
EMRAM: Electronic Medical Record Adoption Model
HIMSS: Healthcare Information and Management Systems Society
EU: European Union
MDR: Medical device regulation
ML: Machine learning
NHL: National Health Service
PICOS: Population, Interventions, Comparators, Outcomes, Study types
PPV: Positive predictive value
PRIOR: Preferred Reporting Items for Overviews of Reviews
PRISMA: Preferred Reporting Items for Systematic reviews and Meta-Analyses
ROBIS: Risk Of Bias In Systematic reviews
SUS: System Usability Scale
US: United States
