# Supplementary material for: Barriers and facilitators to the implementation and adoption of computerised clinical decision support systems: an overview of reviews
Source: Syst Rev. 2026 May 13;15:166. doi: 10.1186/s13643-026-03200-2 (PMC13173960; doi:10.1186/s13643-026-03200-2)
Supplement: Supplementary file 2 — Additional file 2. Search strategy. [file 13643_2026_3200_MOESM2_ESM.pdf]

PubMed/MEDLINE

(decision support systems, clinical[MeSH Terms] OR “decision support system*”[Title/Abstract] OR “decision support tool*”[Title/Abstract] OR “computer* decision support”[Title/Abstract] OR “clinical decision support”[Title/Abstract] OR CDSS[Title/Abstract])

AND

(clinic*[Title/Abstract] OR medic*[Title/Abstract] OR healthcare[Title/Abstract])

AND

(barrier*[Title/Abstract] OR limit*[Title] OR hinder*[Title/Abstract] OR enabler*[Title/Abstract] OR acceptability[Title/Abstract] OR acceptance[Title/Abstract] OR adoption[Title/Abstract] OR uptake[Title/Abstract] OR facilitator*[Title/Abstract] OR attitude to computers[MeSH Terms] OR attitude of health personnel[MeSH Terms] OR human factor*[Title/Abstract] OR contextual factor*[Title/Abstract] OR avoidance[Title/Abstract])

AND

(“systematic review*”[Title/Abstract] OR “scoping review*”[Title/Abstract] OR (review*[Title/ Abstract] AND literature[Title/Abstract]) OR meta-analys*[Title/Abstract] OR “PRISMA”)

IEEE Xplore

((“All Metadata”:decision support) OR (“All Metadata”:CDSS) OR (“Mesh_Terms”:decision support systems, clinical))

AND

((“All Metadata”:clinic*) OR (“All Metadata”:medic*) OR (“All Metadata”:healthcare))

AND

((“All Metadata”:barrier*) OR (“Document Title”:limit*) OR (“All Metadata”:hinder*) OR (“All Metadata”:enabler) OR (“All Metadata”:acceptance) OR (“All Metadata”:adoption) OR (“All Metadata”:acceptability) OR (“All Metadata”:facilitator*) OR (“Mesh_Terms”:attitude to computers) OR (“Mesh_Terms”:attitude of health personnel) OR (“All Metadata”:“human factor*”) OR (“All Metadata”:“contextual factor”) OR (“All Metadata”:uptake) OR (“All Metadata”:avoidance))

AND

((“All Metadata”:review*) OR (“All Metadata”:meta-analys*) OR (“Full Text .AND. Metadata”:PRISMA))

Scopus

(TITLE-ABS-KEY(“decision support system*”) OR TITLE-ABS-KEY(“decision support tool*”) OR TITLE-ABS-KEY(“computer* decision support”) OR TITLE-ABS-KEY(“clinical decision support”) OR TITLE-ABS-KEY(CDSS))

AND

(TITLE-ABS-KEY(clinic*) OR TITLE-ABS-KEY(medic*) OR TITLE-ABS-KEY(healthcare))

AND

(TITLE-ABS-KEY(barrier*) OR TITLE(limit*) OR TITLE-ABS-KEY(hinder*) OR TITLE-ABS-KEY(enabler*) OR TITLE-ABS-KEY(acceptability) OR TITLE-ABS-KEY(acceptance) OR TI-TLE-ABS-KEY(adoption) OR TITLE-ABS-KEY(facilitator*) OR TITLE-ABS-KEY(“human factor*”) OR TITLE-ABS-KEY(“contextual factor*”) OR TITLE-ABS-KEY(uptake) OR TITLE-ABS-KEY(avoidance))

AND

((TITLE-ABS-KEY(review*) AND TITLE-ABS-KEY(literature)) OR TITLE-ABS-KEY(“systematic review*”) OR TITLE-ABS-KEY(“scoping review*”) OR TITLE-ABS-KEY(meta-analys*) OR ALL(“PRISMA”))

Web of Science

(TI=("decision support system*" OR "decision support tool*" OR "computer* decision support" OR "clinical decision support" OR CDSS) OR AB=("decision support system*" OR "decision support tool*" OR "computer* decision support" OR "clinical decision support" OR CDSS))

AND

(TI=(clinic* OR medic* OR healthcare) OR AB=(clinic* OR medic* OR healthcare))

AND

(TI=(barrier* OR limit* OR hinder* OR enabler* OR acceptability OR acceptance OR adoption OR uptake OR facilitator* OR "human factor*" OR "contextual factor*" OR avoidance) OR AB=(barrier* OR hinder* OR enabler* OR acceptability OR acceptance OR adoption OR uptake OR facilitator* OR "human factor*" OR "contextual factor*" OR avoidance))

AND

(TI=((review* AND literature) OR systematic review* OR scoping review* OR meta-analys*) OR AB=((review* AND literature) OR systematic review* OR scoping review* OR meta-analys*) OR ALL=(PRISMA))
