# Supplementary material for: Barriers and facilitators to the implementation and adoption of computerised clinical decision support systems: an overview of reviews
Source: Syst Rev. 2026 May 13;15:166. doi: 10.1186/s13643-026-03200-2 (PMC13173960; doi:10.1186/s13643-026-03200-2)
Supplement: Supplementary file 3 — Additional file 3. Excluded articles with reasons. [file 13643_2026_3200_MOESM3_ESM.pdf]

Excluded articles after full-text assessment

Reference	Reason for exclusion
Aakre CA, Maggio LA, Del Fiol G, Cook DA. Barriers and facilitators to clinical information seeking: a systematic review. J Am Med Inform Assoc. 2019;26:1129–40. doi:10.1093/jamia/ocz065.	No focus on CDSS
Adeloye D, Adigun T, Misra S, Omoregbe N. Assessing the Coverage of E-Health Services in Sub-Saharan Africa. A Systematic Review and Analysis. Methods Inf Med. 2017;56:189–99. doi:10.3414/ME16-05-0012.	No focus on identification of barriers and facilitators
Agarwal S, Glenton C, Tamrat T, Henschke N, Maayan N, Fønhus MS, et al. Decision-support tools via mobile devices to improve quality of care in primary healthcare settings. Cochrane Database Syst Rev. 2021;7:CD012944. doi:10.1002/14651858.CD012944.pub2.	No focus on identification of barriers and facilitators
Ahmadian L, van Engen-Verheul M, Bakhshi-Raiez F, Peek N, Cornet R, Keizer NF de. The role of standardized data and terminological systems in computerized clinical decision support systems: literature review and survey. Int J Med Inform. 2011;80:81–93. doi:10.1016/j.ijmedinf.2010.11.006.	No focus on identification of barriers and facilitators
Alasiri SF, Douiri A, Altukistani S, Porat T, Mousa O. The Role of Clinical Decision Support Systems in Preventing Stroke in Primary Care: A Systematic Review. Perspect Health Inf Manag. 2023;20:1d.	No focus on identification of barriers and facilitators
Aljarboa S, Miah SJ. Acceptance of a Clinical Decision Support System for improving Healthcare Services in Saudi Arabia. In: 2017 4th Asia-Pacific World Congress on Computer Science and Engineering (APWC on CSE); 2017. p. 144–148. doi:10.1109/APWConCSE.2017.00032.	Wrong study type
Aloufi MA. Effect of Clinical Decision Support Systems on Quality of Care by Nurses. International Journal for Quality Research. 2020;14:665–78. doi:10.24874/IJQR14.03-01.	Includes review article
Andargoli AE, Ulapane N, Nguyen TA, Shuakat N, Zelcer J, Wickramasinghe N. Intelligent decision support systems for dementia care: A scoping review. Artif Intell Med. 2024;150:102815. doi:10.1016/j.artmed.2024.102815.	No focus on identification of barriers and facilitators
Anderson JG. Social, ethical and legal barriers to e-health. Int J Med Inform. 2007;76:480–3. doi:10.1016/j.ijmedinf.2006.09.016.	Wrong study type
Angelis G de, Davies B, King J, McEwan J, Cavallo S, Loew L, et al. Information and Communication Technologies for the Dissemination of Clinical Practice Guidelines to Health Professionals: A Systematic Review. JMIR Med Educ. 2016;2:e16. doi:10.2196/mededu.6288.	No focus on identification of barriers and facilitators
Ayani S, Moulaei K, Jahanbakhsh M, Moulaei R. Survey and classification of functional characteristics in neural network technique for the diagnosis of ischemic heart disease: A systematic review. J. Isfahan Med. Sch. 2018;35:1830–9. doi:10.22122/jims.v35i460.8576.	No full-text available
Bamgboje-Ayodele A, Borg DN, McPhail SM, Baysari MT. Clinical decision support systems for chronic obstructive pulmonary disease (COPD) in hospitals: A systematic review. Digit Health. 2023;9:20552076231219107. doi:10.1177/20552076231219107.	No focus on identification of barriers and facilitators
Belard A, Buchman T, Forsberg J, Potter BK, Dente CJ, Kirk A, Elster E. Precision diagnosis: a view of the clinical decision support systems (CDSS) landscape through the lens of critical care. J Clin Monit Comput. 2017;31:261–71. doi:10.1007/s10877-016-9849-1.	Includes review article
Bittmann JA, Haefeli WE, Seidling HM. Modulators Influencing Medication Alert Acceptance: An Explorative Review. Appl Clin Inform. 2022;13:468–85. doi:10.1055/s-0042-1748146.	Includes review article
Borum C. Barriers for Hospital-Based Nurse Practitioners Utilizing Clinical Decision Support Systems: A Systematic Review. Comput Inform Nurs. 2018;36:177–82. doi:10.1097/CIN.0000000000000413.	Includes review article
Bradley PT, Hall N, Maniatopoulos G, Neal RD, Paleri V, Wilkes S. Factors shaping the implementation and use of Clinical Cancer Decision Tools by GPs in primary care: A qualitative framework synthesis. BMJ Open 2021. doi:10.1136/bmjopen-2020-043338.	No focus on CDSS
Brereton TA, Malik MM, Lifson M, Greenwood JD, Peterson KJ, Overgaard SM. The Role of Artificial Intelligence Model Documentation in Translational Science: Scoping Review. Interact J Med Res. 2023;12:e45903. doi:10.2196/45903.	Includes review article
Campion TR, JR, Waitman LR, May AK, Ozdas A, Lorenzi NM, Gadd CS. Social, organizational, and contextual characteristics of clinical decision support systems for intensive insulin therapy: a literature review and case study. Int J Med Inform. 2010;79:31–43. doi:10.1016/j.ijmedinf.2009.09.004.	Wrong study type
Carter J, Sandall J, Shennan AH, Tribe RM. Mobile phone apps for clinical decision support in pregnancy: a scoping review. BMC Med Inform Decis Mak. 2019;19:219. doi:10.1186/s12911-019-0954-1.	Includes review article
Carvalho É, Estrela M, Zapata-Cachafeiro M, Figueiras A, Roque F, Herdeiro MT. E-Health Tools to Improve Antibiotic Use and Resistances: A Systematic Review. Antibiotics (Basel) 2020. doi:10.3390/antibiotics9080505.	No focus on identification of barriers and facilitators

(continued)

Excluded articles after full-text assessment (continued)

Reference	Reason for exclusion
Chintala S. The Application of Deep Learning in Analysing Electronic Health Records for Improved Patient Outcomes. Internat. J. Intel. Syst. Appl. Eng. 2024;12:223–8.	Includes review article
Ciecierski-Holmes T, Singh R, Axt M, Brenner S, Barteit S. Artificial intelligence for strengthening healthcare systems in low- and middle-income countries: a systematic scoping review. NPJ Digit Med. 2022;5:162. doi:10.1038/s41746-022-00700-y.	No focus on identification of barriers and facilitators
Claeys A, van den Eynde R, Rex S. The use of cognitive aids in the operating room: a systematic review. Acta Anaesthesiol. Belg. 2022;73:129–48. doi:10.56126/73.3.18.	Not computerised
Clement J, Maldonado AQ. Augmenting the Transplant Team With Artificial Intelligence: Toward Meaningful AI Use in Solid Organ Transplant. Front Immunol. 2021;12:694222. doi:10.3389/fimmu.2021.694222.	Includes review article
Cresswell K, Mozaffar H, Shah S, Sheikh A. Approaches to promoting the appropriate use of antibiotics through hospital electronic prescribing systems: a scoping review. Int J Pharm Pract. 2017;25:5–17. doi:10.1111/ijpp.12274.	No focus on identification of barriers and facilitators
Deblois S, Chartrand-Lefebvre C, Toporowicz K, Chen Z, Lepanto L. Interventions to Reduce the Overuse of Imaging for Pulmonary Embolism: A Systematic Review. J Hosp Med. 2018;13:52–61. doi:10.12788/jhm.2902.	No focus on CDSS
Delourme S, Redjdal A, Bouaud J, Seroussi B. Measured Performance and Healthcare Professional Perception of Large Language Models Used as Clinical Decision Support Systems: A Scoping Review. Stud Health Technol Inform. 2024;316:841–5. doi:10.3233/SHTI240543.	No focus on identification of barriers and facilitators
Douthit BJ, McCoy AB, Nelson SD. The Impact of Clinical Decision Support on Health Disparities and the Digital Divide. Yearb Med Inform 2023. doi:10.1055/s-0043-1768722.	No focus on identification of barriers and facilitators
Dunsmore J, Duncan E, MacLennan S, N'Dow J, MacLennan S. Effectiveness of de-implementation strategies for low-value prescribing in secondary care: a systematic review. Implement Sci Commun. 2023;4:115. doi:10.1186/s43058-023-00498-0.	No focus on CDSS
Dwivedi R, Ghahramani F, Mahapatra R, editors. Mobile clinical decision support systems – A systematic review: Americas Conference on Information Systems; 2017.	No focus on CDSS
Elyan E, Hussain A, Sheikh A, Elmanama AA, Vuttipittayamongkol P, Hijazi K. Antimicrobial Resistance and Machine Learning: Challenges and Opportunities. IEEE Access. 2022;10:31561–77. doi:10.1109/ACCESS.2022.3160213.	Wrong study type
Evans RP, Bryant LD, Russell G, Absolom K. Trust and acceptability of data-driven clinical recommendations in everyday practice: A scoping review. Int J Med Inform. 2024;183:105342. doi:10.1016/j.ijmedinf.2024.105342.	No focus on identification of barriers and facilitators
Fasola G, Macerelli M, Follador A, Rihawi K, Aprile G, Della Mea V. Health information technology in oncology practice: a literature review. Cancer Inform. 2014;13:131–9. doi:10.4137/CIN.S12417.	Wrong study type
Fernando M, Abell B, Tyack Z, Donovan T, McPhail SM, Naicker S. Using Theories, Models, and Frameworks to Inform Implementation Cycles of Computerized Clinical Decision Support Systems in Tertiary Health Care Settings: Scoping Review. J Med Internet Res. 2023;25:e45163. doi:10.2196/45163.	No focus on identification of barriers and facilitators
Ferreira G, Oliveira E, Stamper J, Coelho A, Paredes H, Rodrigues NF. A Human-Computer Interaction Perspective on Clinical Decision Support Systems: A Systematic Review of Usability, Barriers, and Recommendations for Improvement. In: 2023 IEEE 11th International Conference on Serious Games and Applications for Health (SeGAH); 2023. p. 1–8. doi:10.1109/SeGAH57547.2023.10253790.	No focus on identification of barriers and facilitators
Fiks AG. Designing computerized decision support that works for clinicians and families. Curr Probl Pediatr Adolesc Health Care. 2011;41:60–88. doi:10.1016/j.cppeds.2010.10.006.	Wrong study type
G. BA, A. NH. What Factors Contribute to the Acceptance of Artificial Intelligence on Healthcare Sector: A Systematic Review. In: 2023 3rd International Conference on Intelligent Cybernetics Technology & Applications (ICICyTA); 2023. p. 477–482. doi:10.1109/ICICyTA60173.2023.10428744.	Includes review article
Gaitanou P, Garoufallou E, Balatsoukas P. The effectiveness of big data in health care: A systematic review. In: S C, Hochschule Karlsruhe Technik und Wirtschaft, Fakultät IMM, Amalienstr. 81-87, Karlsruhe, 76133, R S, E G, -A SM, University of Alcalá, Computer Science Department, Polytechnic School, Ctra. Barcelona km. 33.6, Alcalá de Henares, Madrid, 28871, editors. Communications in Computer and Information Science: Springer Verlag; 2014. p. 141–153. doi:10.1007/978-3-319-13674-5_14.	No focus on CDSS

(continued)

Excluded articles after full-text assessment (continued)

Reference	Reason for exclusion
Gearhart A, Gaffar S, Chang AC. A primer on artificial intelligence for the paediatric cardiologist. Cardiol. Young. 2020;30:934–45. doi:10.1017/S1047951120001493.	Wrong study type
Geduld C, Muller H, Saunders CJ. Factors which affect the application and implementation of a spinal motion restriction protocol by prehospital providers in a low resource setting: A scoping review. Afr. J. Emerg. Med. 2022;12:393–405. doi:10.1016/j.afjem.2022.08.005.	Not computerised
Gillam J, Davies N, Aworinde J, Yorganci E, Anderson JE, Evans C. Implementation of eHealth to Support Assessment and Decision-making for Residents With Dementia in Long-term Care: Systematic Review. J Med Internet Res. 2022;24:e29837. doi:10.2196/29837.	No focus on CDSS
Goddard K, Roudsari A, Wyatt JC. Automation bias: a systematic review of frequency, effect mediators, and mitigators. J Am Med Inform Assoc. 2012;19:121–7. doi:10.1136/amiajnl-2011-000089.	No focus on identification of barriers and facilitators
Hill A, Morrissey D, Marsh W. What characteristics of clinical decision support system implementations lead to adoption for regular use? A scoping review. BMJ Health Care Inform 2024. doi:10.1136/bmjhci-2024-101046.	No focus on identification of barriers and facilitators
Hoppe D, Karimi L, Khalil H. Mapping the research addressing prescription drug monitoring programs: A scoping review. Drug Alcohol Rev. 2022;41:803–17. doi:10.1111/dar.13431.	Includes review article
Horsky J, Schiff GD, Johnston D, Mercincavage L, Bell D, Middleton B. Interface design principles for usable decision support: A targeted review of best practices for clinical prescribing interventions. J Biomed Inform. 2012;45:1202–16. doi:10.1016/j.jbi.2012.09.002.	No focus on identification of barriers and facilitators
Hussain MI, Reynolds TL, Zheng K. Medication safety alert fatigue may be reduced via interaction design and clinical role tailoring: a systematic review. J Am Med Inform Assoc. 2019;26:1141–9. doi:10.1093/jamia/ocz095.	No focus on identification of barriers and facilitators
Kaiser C, Ackerhans S, Huynh T, Denzinger J, Riegler M, Dumitrescu D, et al. Challenges in the Implementation of Disruptive Innovations in Health Care Organizations. Stud Health Technol Inform. 2023;309:145–9. doi:10.3233/SHTI230763.	Wrong study type
Kalsi S, French H, Chhaya S, Madani H, Mir R, Anosova A, Dubash S. The Evolving Role of Artificial Intelligence in Radiotherapy Treatment Planning—A Literature Review. Clin. Oncol. 2024;36:596–605. doi:10.1016/j.clon.2024.06.005.	Wrong study type
Keitel K, D'Acremont V. Electronic clinical decision algorithms for the integrated primary care management of febrile children in low-resource settings: review of existing tools. Clin Microbiol Infect. 2018;24:845–55. doi:10.1016/j.cmi.2018.04.014.	No focus on identification of barriers and facilitators
Kennedy EE, Bowles KH. Human Factors Considerations in Transitions in Care Clinical Decision Support System Implementation Studies. AMIA Annu Symp Proc. 2021;2021:621–30.	No focus on identification of barriers and facilitators
Keyworth C, Hart J, Armitage CJ, Tully MP. What maximizes the effectiveness and implementation of technology-based interventions to support healthcare professional practice? A systematic literature review. BMC Med Inform Decis Mak 2018. doi:10.1186/s12911-018-0661-3.	No focus on CDSS
Khalifa M, Gallego B. Grading and assessment of clinical predictive tools for paediatric head injury: a new evidence-based approach. BMC Emerg. Med. 2019;19:35. doi:10.1186/s12873-019-0249-y.	No focus on identification of barriers and facilitators
Khalil C. The information systems in the delivery of health information and care services: A review of the literature. In: 19th Symposium of the Association Information and Management 2014, AIM 2014: Association Information and Management; 2014. p. 75–79.	No full-text available
Khan N, Nwafor Okoli C, Ekpin V, Attai K, Chukwudi N, Sabi H, et al. Adoption and utilization of medical decision support systems in the diagnosis of febrile Diseases: A systematic literature review. Expert Sys Appl 2023. doi:10.1016/j.eswa.2023.119638.	Includes patient-facing CDSS
Kilsdonk E, Peute LWP, Knijnenburg SL, Jaspers MWM. Factors known to influence acceptance of clinical decision support systems. Stud Health Technol Inform. 2011;169:150–4.	No focus on identification of barriers and facilitators
Knop M, Weber S, Mueller M, Niehaves B. Human Factors and Technological Characteristics Influencing the Interaction of Medical Professionals With Artificial Intelligence-Enabled Clinical Decision Support Systems: Literature Review. JMIR Hum Factors. 2022;9:e28639. doi:10.2196/28639.	Includes review article
Kramer HS, Drews FA. Checking the lists: A systematic review of electronic checklist use in health care. J Biomed Inform. 2017;71:S6-S12. doi:10.1016/j.jbi.2016.09.006.	No focus on identification of barriers and facilitators
Kux BR, Majeed RW, Ahlbrandt J, Röhrig R. Factors Influencing the Implementation and Distribution of Clinical Decision Support Systems (CDSS). Stud Health Technol Inform. 2017;243:127–31.	Wrong study type

(continued)

Excluded articles after full-text assessment (continued)

Reference	Reason for exclusion
Kyrimi E, Dube K, Fenton N, Fahmi A, Neves MR, Marsh W, McLachlan S. Bayesian networks in healthcare: What is preventing their adoption? Artif Intell Med. 2021;116:102079. doi:10.1016/j.artmed.2021.102079.	Includes patient-facing CDSS
Lai Y, Sukhwal PC, Kankanhalli A, editors. Explanations in clinical DSS: A review and research agenda: Association for Information Systems; 2020.	No focus on identification of barriers and facilitators
Lettieri E, Radaelli G, Masella C. Information systems and change management in healthcare: The (un)solved quest for changing physicians' behaviour. Int. J. Inf. Syst. Change Mange. 2010;4:226–45. doi:10.1504/IJISCM.2010.033077.	Includes review article
Lewkowicz D, Wohlbrandt A, Boettinger E. Economic impact of clinical decision support interventions based on electronic health records. BMC Health Serv Res 2020. doi:10.1186/s12913-020-05688-3.	No focus on identification of barriers and facilitators
Liu S, Reese TJ, Kawamoto K, Del Fiol G, Weir C. Toward Optimized Clinical Decision Support: A Theory-Based Approach. In: 2020 IEEE International Conference on Healthcare Informatics (ICHI); 2020. p. 1–2. doi:10.1109/ICHI48887.2020.9374346.	Wrong study type
Lluch M. Healthcare professionals' organisational barriers to health information technologies-a literature review. Int J Med Inform. 2011;80:849–62. doi:10.1016/j.ijmedinf.2011.09.005.	Includes review article
Marc DT, Khairat SS. Why do physicians have difficulty accepting clinical decision support systems? Stud Health Technol Inform. 2013;192:1202.	Wrong study type
Mecham ID, Vines C, Dean NC. Community-acquired pneumonia management and outcomes in the era of health information technology. Respirology. 2017;22:1529–35. doi:10.1111/resp.13132.	No focus on CDSS
Middleton B, Sittig DF, Wright A. Clinical Decision Support: a 25 Year Retrospective and a 25 Year Vision. Yearb Med Inform. 2016;Suppl 1:S103-16. doi:10.15265/IYS-2016-s034.	Wrong study type
Miller K, Mosby D, Capan M, Kowalski R, Ratwani R, Noaiseh Y, et al. Interface, information, interaction: a narrative review of design and functional requirements for clinical decision support. J Am Med Inform Assoc. 2018;25:585–92. doi:10.1093/jamia/ocx118.	Includes review article
Murthi S, Martini N, Falconer N, Scahill S. Evaluating EHR-Integrated Digital Technologies for Medication-Related Outcomes and Health Equity in Hospitalised Adults: A Scoping Review. J Med Syst. 2024;48:79. doi:10.1007/s10916-024-02097-5.	No focus on identification of barriers and facilitators
Newton N, Bamgboje-Ayodele A, Forsyth R, Tariq A, Baysari MT. Does Involving Clinicians in Decision Support Development Facilitate System Use Over Time? A Systematic Review. Stud Health Technol Inform. 2023;304:11–5. doi:10.3233/SHTI230359.	No focus on identification of barriers and facilitators
Newton N, Bamgboje-Ayodele A, Forsyth R, Tariq A, Baysari MT. How Are Clinicians' Acceptance and Use of Clinical Decision Support Systems Evaluated Over Time? A Systematic Review. Stud Health Technol Inform. 2024;310:259–63. doi:10.3233/SHTI230967.	No focus on identification of barriers and facilitators
Noteboom C, Zeng D, Sutrave K, Behrens A, Godasu R, Chauhan A. Data-Driven Clinical Decision Support Systems Theory and Research. In: Encyclopedia of Data Science and Machine Learning: IGI Global; 2022. p. 1373–1390. doi:10.4018/978-1-7998-9220-5.ch081.	No focus on identification of barriers and facilitators
Olakotan OO, Mohd Yusof M. The appropriateness of clinical decision support systems alerts in supporting clinical workflows: A systematic review. Health Informatics J. 2021;27:14604582211007536. doi:10.1177/14604582211007536.	No focus on identification of barriers and facilitators
Olakotan OO, Yusof MM. Evaluating the alert appropriateness of clinical decision support systems in supporting clinical workflow. J Biomed Inform. 2020;106:103453. doi:10.1016/j.jbi.2020.103453.	No focus on identification of barriers and facilitators
Rahim NR, Nordin S, Dom RM. Review on barriers and considerations of Clinical Decision Support System for medication prescribing. In: 2015 IEEE Student Conference on Research and Development (SCOReD); 2015. p. 489–494. doi:10.1109/SCORED.2015.7449384.	Wrong study type
Rajamani S, Hultman G, Bakker C, Melton GB. The role of organizational culture in health information technology implementations: A scoping review. Learn Health Syst. 2022;6:e10299. doi:10.1002/lrh2.10299.	No focus on CDSS
Roque AC, André T. Mammography and computerized decision systems: A review. In: Annals of the New York Academy of Sciences: New York Academy of Sciences; 2002. p. 83–94. doi:10.1111/j.1749-6632.2002.tb04890.x.	Wrong study type

(continued)

Excluded articles after full-text assessment (continued)

Reference	Reason for exclusion
Sellin J, Pantel JT, Börsch N, Conrad R, Mücke M. Short paths to diagnosis with artificial intelligence: systematic literature review on diagnostic decision support systems. [Kurze Wege zur Diagnose mit künstlicher Intelligenz – systematische Literaturrecherche zu „diagnostic decision support systems“]. Schmerz. 2024;38:19–27. doi:10.1007/s00482-023-00777-8.	No focus on identification of barriers and facilitators
Semple SJ, Roughead EE. Medication safety in acute care in Australia: where are we now? Part 2: a review of strategies and activities for improving medication safety 2002-2008. Aust New Zealand Health Policy. 2009;6:24. doi:10.1186/1743-8462-6-24.	Includes review article
Shahil Feroz A, Afzal N, Seto E. Exploring digital health interventions for pregnant women at high risk for pre-eclampsia and eclampsia in low-income and-middle-income countries: a scoping review. BMJ Open. 2022;12:e056130. doi:10.1136/bmjopen-2021-056130.	No focus on CDSS
Smith MY, Depue JD, Rini C. Computerized decision-support systems for chronic pain management in primary care. Pain Med. (USA). 2007;8:S155-S166. doi:10.1111/j.1526-4637.2007.00278.x.	No focus on identification of barriers and facilitators
Souza-Pereira L, Ouhbi S, Pombo N. Quality-in-use characteristics for clinical decision support system assessment. Comput Methods Programs Biomed. 2021;207:106169. doi:10.1016/j.cmpb.2021.106169.	No focus on identification of barriers and facilitators
Stilwell L, Golonka M, Ankoma-Sey K, Yancy M, Kaplan S, Terrell L, Gifford EJ. Electronic Health Record Tools to Identify Child Maltreatment: Scoping Literature Review and Key Informant Interviews. Acad Pediatr. 2022;22:718–28. doi:10.1016/j.acap.2022.01.017.	Wrong study type
Stipelman CH, Kukhareva PV, Trepman E, Nguyen Q-T, Valdez L, Kenost C, et al. Electronic Health Record-Integrated Clinical Decision Support for Clinicians Serving Populations Facing Health Care Disparities: Literature Review. Yearb Med Inform. 2022;31:184–98. doi:10.1055/s-0042-1742518.	No focus on identification of barriers and facilitators
Syed W, Alsufayan MA, AlRammah AA, Alsaleh SS, Samreen S, Almutairi AE, et al. Exploring Clinical Decision Support System in Health-Care Settings - Challenges and Barriers to Implementation - A Literature Review. ASIAN JOURNAL OF PHARMACEUTICS. 2022;16:402–6.	Wrong study type
Tai AMY, Kim JJ, Schmeckenbecher J, Kitchin V, Wang J, Kazemi A, et al. Clinical decision support systems in addiction and concurrent disorders: A systematic review and meta-analysis. J. Eval. Clin. Pract. 2024. doi:10.1111/jep.14069.	Includes patient-facing CDSS
Tran DM, Thwaites CL, van Nuil JI, McKnight J, Luu AP, Paton C. Digital Health Policy and Programs for Hospital Care in Vietnam: Scoping Review. J Med Internet Res. 2022;24:e32392. doi:10.2196/32392.	No focus on CDSS
Tucci V, Saary J, Doyle TE. Factors influencing trust in medical artificial intelligence for healthcare professionals: a narrative review. J. Med. Artif. Intell. 2022. doi:10.21037/jmai-21-25.	No focus on identification of barriers and facilitators
Ulapane N, Wickramasinghe N. Critical issues in mobile solution-based clinical decision support systems: A scoping review. In: Optimizing Health Monitoring Systems With Wireless Technology: IGI Global; 2020. p. 32–44. doi:10.4018/978-1-5225-6067-8.ch004.	Includes patient-facing CDSS
van de Burgt, Britt W M, Wasylewicz ATM, Dullemond B, Grouls RJE, Egberts TCG, Bouwman A, Korsten EMM. Combining text mining with clinical decision support in clinical practice: a scoping review. J Am Med Inform Assoc. 2023;30:588–603. doi:10.1093/jamia/ocac240.	No focus on identification of barriers and facilitators
White A, Thomas DSK, Ezeanochie N, Bull S. Health Worker mHealth Utilization: A Systematic Review. Comput Inform Nurs. 2016;34:206–13. doi:10.1097/CIN.0000000000000231.	No focus on CDSS
Wu HW, Davis PK, Bell DS. Advancing clinical decision support using lessons from outside of healthcare: an interdisciplinary systematic review. BMC Med Inform Decis Mak. 2012;12:90. doi:10.1186/1472-6947-12-90.	Not healthcare related
Yusof MM, Paul RJ, Stergioulas L. Health information systems evaluation: a focus on clinical decision supports system. Stud Health Technol Inform. 2005;116:855–60.	Wrong study type
