# Supplementary material for: Barriers and facilitators to the implementation and adoption of computerised clinical decision support systems: an overview of reviews
Source: Syst Rev. 2026 May 13;15:166. doi: 10.1186/s13643-026-03200-2 (PMC13173960; doi:10.1186/s13643-026-03200-2)
Supplement: Supplementary file 4 — Additional file 4. List of primary studies. [file 13643_2026_3200_MOESM4_ESM.pdf]

List of primary studies

Reference
Tierney WM, Miller ME, Overhage JM, McDonald CJ. Physician inpatient order writing on microcomputer workstations. Effects on resource utilization. JAMA. 1993;269:379–83. doi:10.1001/jama.1993.03500030077036.
Gardner RM, Lundsgaarde HP. Evaluation of User Acceptance of a Clinical Expert System. Journal of the American Medical Informatics Association : JAMIA. 1994;1:428–38. doi:10.1136/jamia.1994.95153432.
Sands DZ, Safran C. Closing the loop of patient care–a clinical trial of a computerized discharge medication program. Proceedings. Symposium on Computer Applications in Medical Care. 1994;NA.
Willson DF, Ashton C, Wingate N, Goff C, Horn SD, Davies M, Buxton R. Computerized support of pressure ulcer prevention and treatment protocols. Proceedings. Symposium on Computer Applications in Medical Care. 1995;NA:646–50.
Hobbs FDR, Delaney B, A C, Kenkre JE. A prospective controlled trial of computerized decision support for lipid management in primary care. Family practice. 1996;13:133–7. doi:10.1093/fampra/13.2.133.
Rotman BL, Sullivan AN, McDonald TW, Brown BW, DeSmedt P, Goodnature D, et al. A randomized controlled trial of a computer-based physician workstation in an outpatient setting: implementation barriers to outcome evaluation. Journal of the American Medical Informatics Association : JAMIA. 1996;3:340–8. doi:10.1136/jamia.1996.97035025.
Walton R, Gierl C, Yudkin P, Mistry H, Vessey MP, Fox J. Evaluation of computer support for prescribing (CAPSULE) using simulated cases. BMJ (Clinical research ed.). 1997;315:791–5. doi:10.1136/bmj.315.7111.791.
Zielstorff RD, Estey G, Vickery A, Hamilton G, Fitzmaurice JB, Barnett G. AMIA - Evaluation of a decision support system for pressure ulcer prevention and management: preliminary findings. Proceedings : a conference of the American Medical Informatics Association. AMIA Fall Symposium. 1997;NA:248–52.
Klercker T af, Zetraeus S. Dilemmas in introducing World Wide Web-based information technology in primary care: a focus group study. Fam Pract. 1998;15:205–10. doi:10.1093/fampra/15.3.205.
Sowerby Centre for Health Informatics at Newcastle. PRODIGY phase one summary report. R-38. 1998.
Grundmeier RW, Johnson K. AMIA - Housestaff attitudes toward computer-based clinical decision support. Proceedings. AMIA Symposium. 1999;NA:266–70.
Hetlevik I, others. Implementing clinical guidelines in the treatment of hypertension in general practice: evaluation of patient outcome related to implementation of a computer-based clinical decision support system. Scandinavian Journal of Primary Health Care. 1999;17:35–40.
Tai SS, Nazareth I, Donegan C, Haines A. Evaluation of general practice computer templates : Lessons from a pilot randomised controlled trial. Methods of information in medicine. 1999;38:177–81. doi:10.1055/s-0038-1634189.
Demakis JG, Beauchamp C, Cull WL, Denwood R, Eisen SA, Lofgren R, et al. Improving Residents' Compliance With Standards of Ambulatory CareResults From the VA Cooperative Study on Computerized Reminders. JAMA. 2000;284:1411–6. doi:10.1001/jama.284.11.1411.
Eccles MP, Grimshaw JM, Steen N, Parkin D, Purves I, McColl E, Rousseau N. The design and analysis of a randomized controlled trial to evaluate computerized decision support in primary care: the COGENT study. Family practice. 2000;17:180–6. doi:10.1093/fampra/17.2.180.
Gerbert B, Bronstone A, Maurer T, Hofmann R, Berger TG. Decision support software to help primary care physicians triage skin cancer: a pilot study. Archives of dermatology. 2000;136:187–92. doi:10.1001/archderm.136.2.187.
Hetlevik I, others. Implementing clinical guidelines in the treatment of diabetes mellitus in general practice: Evaluation of effort, process, and patient outcome related to implementation of a computer-based decision support system. International Journal of Technology Assessment in Health Care. 2000;16:210–27.
Neville RG, McCowan C, Ricketts IW, Fonseca J, Invernizzi G, Stey C. Guidelines into practice: An international pilot study of "Asthma Crystal Byte". Primary care respiratory journal : journal of the General Practice Airways Group. 2000;9:56–8. doi:10.1038/pcrj.2000.21.
Quaglini S, Grandi M, Baiardi P, Mazzoleni MC, Fassino C, Franchi G, Melino S. A computerised guideline for pressure ulcer prevention. International journal of medical informatics. 2000;58:207–17. doi:10.1016/s1386-5056(00)00088-5.
Shiffman RN, Freudigman NA, Brandt C, Liaw Y, Dd N. A Guideline Implementation System Using Handheld Computers for Office Management of Asthma: Effects on Adherence and Patient Outcomes. Pediatrics. 2000;105:767–73. doi:10.1542/peds.105.4.767.
Sokolove PE, Lee BS, Krawczyk JA, Banos PT, Gregson AL, Boyce DM, et al. Implementation of an emergency department triage procedure for the detection and isolation of patients with active pulmonary tuberculosis. Ann Emerg Med. 2000;35:327–36. doi:10.1016/s0196-0644(00)70050-3.
Chan SSW, Yuen EHY, Kew J, Cheung WL, Cocks RA. Community-acquired pneumonia—implementation of a prediction rule to guide selection of patients for outpatient treatment. Eur J Emerg Med. 2001;8:279–86.
Krall MA, Sittig DF. AMIA - Subjective assessment of usefulness and appropriate presentation mode of alerts and reminders in the outpatient setting. Proceedings. AMIA Symposium. 2001;NA:334–8.

(continued)

List of primary studies (continued)

Reference
McCowan C, Neville RG, Ricketts IW, Warner FC, Hoskins G, Thomas GE. Lessons from a randomized controlled trial designed to evaluate computer decision support software to improve the management of asthma. Medical informatics and the Internet in medicine. 2001;26:191–201. doi:10.1080/14639230110067890.
Mikulich VJ, Liu Y-CA, Steinfeldt J, Schriger DL. Implementation of clinical guidelines through an electronic medical record: physician usage, satisfaction and assessment. International journal of medical informatics. 2001;63:169–78. doi:10.1016/s1386-5056(01)00177-0.
Rocha BH, Christenson JC, Evans RS, Gardner RM. Clinicians' response to computerized detection of infections. Journal of the American Medical Informatics Association : JAMIA. 2001;8:117–25. doi:10.1136/jamia.2001.0080117.
van Wijk MA, van der Lei J, Mosseveld M, Bohnen AM, van Bemmel JH. Assessment of decision support for blood test ordering in primary care. a randomized trial. Ann Intern Med. 2001;134:274–81. doi:10.7326/0003-4819-134-4-200102200-00010.
Eccles MP, McColl E, Steen N, Rousseau N, Grimshaw JM, Parkin D, Purves I. Effect of computerised evidence based guidelines on management of asthma and angina in adults in primary care: cluster randomised controlled trial. BMJ (Clinical research ed.). 2002;325:941. doi:10.1136/bmj.325.7370.941.
Glassman PA, Simon B, Belperio P, Lanto AB. Improving recognition of drug interactions: benefits and barriers to using automated drug alerts. Medical care. 2002;40:1161–71. doi:10.1097/00005650-200212000-00004.
Knaup P, Wiedemann T, Bachert A, Creutzig U, Haux R, Schilling FH. Efficiency and safety of chemotherapy plans for children: CATIPO-a nationwide approach. Artificial intelligence in medicine. 2002;24:229–42. doi:10.1016/s0933-3657(01)00106-3.
Krall MA, Sittig DF. AMIA - Clinician's assessments of outpatient electronic medical record alert and reminder usability and usefulness requirements. Proceedings. AMIA Symposium. 2002;NA:400–4.
Logan ECM, Yates JM, Stewart RM, Fielding K, Kendrick D. Investigation and management of iron deficiency anaemia in general practice: a cluster randomised controlled trial of a simple management prompt. Postgraduate medical journal. 2002;78:533–7. doi:10.1136/pmj.78.923.533.
Magnus D, Rodgers S, Avery AJ. GPs' views on computerized drug interaction alerts: questionnaire survey. Journal of clinical pharmacy and therapeutics. 2002;27:377–82. doi:10.1046/j.1365-2710.2002.00434.x.
Payne TH, Nichol WP, Hoey P, Savarino J. AMIA - Characteristics and override rates of order checks in a practitioner order entry system. Proceedings. AMIA Symposium. 2002;NA:602–6.
Trelle S. Information management and reading habits of German diabetologists: a questionnaire survey. Diabetologia. 2002;45:764–74. doi:10.1007/s00125-002-0807-8.
Adams WG, Fuhlbrigge AL, Miller CW, Panek CG, Gi Y, Loane KC, et al. AMIA - TLC-Asthma: An Integrated Information System for Patient-centered Monitoring, Case Management, and Point-of-Care Decision Support. AMIA … Annual Symposium proceedings. AMIA Symposium. 2003;2003:1–5.
Ahearn MD, Kerr SJ. General practitioners' perceptions of the pharmaceutical decision-support tools in their prescribing software. The Medical journal of Australia. 2003;179:34–7. doi:10.5694/j.1326-5377.2003.tb05415.x.
Bindels R, Hasman A, Derickx M, van Wersch JWJ, Winkens R. User satisfaction with a real-time automated feedback system for general practitioners: a quantitative and qualitative study. International journal for quality in health care : journal of the International Society for Quality in Health Care. 2003;15:501–8. doi:10.1093/intqhc/mzg076.
Chang P, Tzeng Y-M, Wu S-C, Sang Y-Y, Chen S-S. Development and comparison of user acceptance of advanced comprehensive triage PDA support system with a traditional terminal alternative system. In: ; 2003. p. 140–144.
Colombet I, Dart T, Leneveut L, Zunino S, Ménard J, Chatellier G. A computer decision aid for medical prevention: a pilot qualitative study of the Personalized Estimate of Risks (EsPeR) system. BMC medical informatics and decision making. 2003;3:13. doi:10.1186/1472-6947-3-13.
Flottorp S, Håvelsrud K, Oxman AD. Process evaluation of a cluster randomized trial of tailored interventions to implement guidelines in primary care—why is it so hard to change practice? Family practice. 2003;20:333–9. doi:10.1093/fampra/cmg316.
Gosling AS, Westbrook JI, Coiera E. Variation in the use of online clinical evidence: a qualitative analysis. International journal of medical informatics. 2003;69:1–16. doi:10.1016/s1386-5056(02)00046-1.
Jazayeri D, Farmer P, Nevil P, Mukherjee JS, Leandre F, Fraser HSF. AMIA - An Electronic Medical Record system to support HIV treatment in rural Haiti. AMIA … Annual Symposium proceedings. AMIA Symposium. 2003;2003:878.
Kuperman GJ, Gandhi TK, Bates DW. Effective drug-allergy checking: methodological and operational issues. Journal of biomedical informatics. 2003;36:70–9. doi:10.1016/s1532-0464(03)00063-7.
Meigs JB, et al. A controlled trial of web-based diabetes disease management: the MGH diabetes primary care improvement project. Diabetes Care. 2003;26:750–7.
den Pain, Fielden K, Shibl R. Opinions on the use of clinical decision support systems for paediatric prescribing in a New Zealand hospital. Logistics Information Management. 2003;16:201–6. doi:10.1108/09576050310483790.

(continued)

List of primary studies (continued)

Reference
Robert C Burack, Phyllis A Gimotty, Michael Simon, Anita Moncrease, Peter Dews. The effect of adding Pap smear information to a mammography reminder system in an HMO: results of randomized controlled trial. Preventive Medicine. 2003;36:547–54. doi:10.1016/S0091-7435(02)00062-2.
Rousseau N, McColl E, Newton JN, Grimshaw JM, Eccles MP. Practice based, longitudinal, qualitative interview study of computerised evidence based guidelines in primary care. BMJ (Clinical research ed.). 2003;326:314. doi:10.1136/bmj.326.7384.314.
Schellhase KG, Koepsell TD, Norris TE. Providers' reactions to an automated health maintenance reminder system incorporated into the patient's electronic medical record. The Journal of the American Board of Family Practice. 2003;16:312–7. doi:10.3122/jabfm.16.4.312.
Short D, Frischer M, Bashford J. The development and evaluation of a computerised decision support system for primary care based upon 'patient profile decision analysis'. Informatics in primary care. 2003;11:195–202. doi:10.14236/jhi.v11i4.567.
Weingart SN, Toth M, Sands DZ, Aronson MD, Davis RB, Phillips RS. Physicians' Decisions to Override Computerized Drug Alerts in Primary Care. Archives of internal medicine. 2003;163:2625–31. doi:10.1001/archinte.163.21.2625.
Bury J, Hurt C, Roy A, Bradburn M, Cross S, Fox J, Saha V. A quantitative and qualitative evaluation of LISA, a decision support system for chemotherapy dosing in childhood Acute Lymphoblastic Leukaemia. In: MEDINFO 2004: IOS Press; 2004. p. 197–201. doi:10.3233/978-1-60750-949-3-197.
Chang P, Hsu Y-S, Tzeng Y-M, Sang Y-Y, Hou I-C, Kao W-F. The development of intelligent, triage-based, mass-gathering emergency medical service PDA support systems. J Nurs Res. 2004;12:227–36. doi:10.1097/01.JNR.0000387506.06502.90.
Christakis DA, Wright JA. Can Continuity of Care Be Improved? Results From a Randomized Pilot Study. Ambulatory Pediatrics. 2004;4:336–9. doi:10.1367/A03-166R.1.
Feldstein AC, Simon SR, Schneider JL, Krall MA, Laferriere D, Smith DH, et al. How to Design Computerized Alerts to Ensure Safe Prescribing Practices. Joint Commission journal on quality and safety. 2004;30:602–13. doi:10.1016/s1549-3741(04)30071-7.
Fraser HSF, Jazayeri D, Nevil P, Karacaoglu Y, Farmer PE, Lyon E, et al. An information system and medical record to support HIV treatment in rural Haiti. BMJ. 2004;329:1142–6. doi:10.1136/bmj.329.7475.1142.
Fung CH, Woods JN, Asch SM, Glassman PA, Doebbeling BN. Variation in Implementation and Use of Computerized Clinical Reminders in an Integrated Healthcare System. The American journal of managed care. 2004;10:878–85.
Hollander JE, Sease KL, Sparano DM, Sites FD, Shofer FS, Baxt WG. Effects of neural network feedback to physicians on admit/discharge decision for emergency department patients with chest pain. Ann Emerg Med. 2004;44:199–205. doi:10.1016/j.annemergmed.2004.02.037.
Horsky J, Kaufman DR, Patel VL. MedInfo - Computer-based drug ordering: evaluation of interaction with a decision-support system. Studies in health technology and informatics. 2004;107:1063–7.
Hwang H-G, Chang I-C, Hung W-F, Sung M-L, Yen DC. The design and evaluation of clinical decision support systems in the area of pharmacokinetics. Medical informatics and the Internet in medicine. 2004;29:239–51. doi:10.1080/14639230400009158.
Murray MD, Harris LE, Overhage JM, Zhou X-H, Eckert GJ, Smith FE, et al. Failure of computerized treatment suggestions to improve health outcomes of outpatients with uncomplicated hypertension: results of a randomized controlled trial. Pharmacotherapy. 2004;24:324–37. doi:10.1592/phco.24.4.324.33173.
Patterson ES, Nguyen AD, Halloran JP, Asch SM. Human Factors Barriers to the Effective Use of Ten HIV Clinical Reminders. Journal of the American Medical Informatics Association : JAMIA. 2004;11:50–9. doi:10.1197/jamia.m1364.
Rahmner PB, Andersén-Karlsson E, Arnhjort T, Eliasson M, Gustafsson LL, Jacobsson L, et al. Physicians' perceptions of possibilities and obstacles prior to implementing a computerised drug prescribing support system. International journal of health care quality assurance incorporating Leadership in health services. 2004;17:173–9. doi:10.1108/09526860410541487.
Rosenbloom ST, Talbert DA, Aronsky D. Clinicians' perceptions of clinical decision support integrated into computerized provider order entry. International journal of medical informatics. 2004;73:433–41. doi:10.1016/j.ijmedinf.2004.04.001.
Short D, Frischer M, Bashford J. Barriers to the adoption of computerised decision support systems in general practice consultations: a qualitative study of GPs' perspectives. International journal of medical informatics. 2004;73:357–62. doi:10.1016/j.ijmedinf.2004.02.001.
Taylor L, Tamblyn R. MedInfo - Reasons for physician non-adherence to electronic drug alerts. Studies in health technology and informatics. 2004;107:1101–5.
Twiggs JE, Fifield J, Jackson E, Cushman RA, Apter AJ. Treating asthma by the guidelines: developing a medication management information system for use in primary care. Disease management : DM. 2004;7:244–60. doi:10.1089/dis.2004.7.244.
Warburton RN, Parke B, Church W, McCusker J. Identification of seniors at risk: process evaluation of a screening and referral program for patients aged ≥ 75 in a community hospital emergency department. Int J Health Care Qual Assur Inc Leadersh Health Serv. 2004;17:339–48. doi:10.1108/09526860410557598.
Williams J, Cheung W-Y, Price DE, Tansey R, Russell IT, Duane PD, et al. Clinical guidelines online: do they improve compliance? Postgraduate medical journal. 2004;80:415–9. doi:10.1136/pgmj.2003.015974.

(continued)

List of primary studies (continued)

Reference
Young AS, Mintz J, Cohen AN, Chinman M. A Network-Based System to Improve Care for Schizophrenia: The Medical Informatics Network Tool (MINT). Journal of the American Medical Informatics Association : JAMIA. 2004;11:358–67. doi:10.1197/jamia.m1492.
Apkon M, Mattera JA, Lin Z, Herrin J, Bradley EH, Carbone M, et al. A randomized outpatient trial of a decision-support information technology tool. Archives of internal medicine. 2005;165:2388–94. doi:10.1001/archinte.165.20.2388.
Avery AJ, Savelyich BSP, Sheikh A, Ja C, Morris C, Fernando B, et al. Identifying and establishing consensus on the most important safety features of GP computer systems: e-Delphi study. Informatics in primary care. 2005;13:3–11. doi:10.14236/jhi.v13i1.575.
Bloomfield HE, Nelson DB, van Ryn M, Neil BJ, Koets NJ, Basile JN, et al. A trial of education, prompts, and opinion leaders to improve prescription of lipid modifying therapy by primary care physicians for patients with ischemic heart disease. Quality & safety in health care. 2005;14:258–63. doi:10.1136/qshc.2004.012617.
Bury J, Hurt CN, Roy A, Cheesman L, Bradburn M, Cross SS, et al. LISA: a web-based decision-support system for trial management of childhood acute lymphoblastic leukaemia. British journal of haematology. 2005;129:746–54. doi:10.1111/j.1365-2141.2005.05541.x.
Clarke HF, Bradley C, Whytock S, Handfield S, van der Wal R, Gundry S. Pressure ulcers: implementation of evidence-based nursing practice. Journal of advanced nursing. 2005;49:578–90. doi:10.1111/j.1365-2648.2004.03333.x.
Cobos A, Vilaseca J, Asenjo C, Pedro-Botet J, Sánchez E, Val A, et al. Cost Effectiveness of a Clinical Decision Support System Based on the Recommendations of the European Society of Cardiology and Other Societies for the Management of Hypercholesterolemia. Disease Management and Health Outcomes. 2005;13:421–32. doi:10.2165/00115677-200513060-00007.
Derose SF, Dudl JR, Benson VM, Contreras R, Nakahiro RK, Ziel FH. Point-of-Service reminders for prescribing cardiovascular medications. Am J Manag Care. 2005;11:298–304.
Feldstein AC, Smith DH, Robertson NR, Kovach CA, Soumerai SB, Simon SR, et al. Decision Support System Design and Implementation for Outpatient Prescribing: The Safety in Prescribing Study. NA. 2005;NA.
Graber ML, Franklin N, Gordon R. Diagnostic error in internal medicine. Arch Intern Med. 2005;165:1493–9.
Kenealy T, Arroll B, Petrie KJ. Patients and computers as reminders to screen for diabetes in family practice. Randomized-controlled trial. Journal of general internal medicine. 2005;20:916–21. doi:10.1111/j.1525-1497.2005.0197.x.
Koppel R, Metlay JP, Cohen A, Abaluck B, Localio AR, Kimmel SE, Strom BL. Role of computerized physician order entry systems in facilitating medication errors. JAMA. 2005;293:1197–203. doi:10.1001/jama.293.10.1197.
Marcy TW, Skelly J, Shiffman RN, Flynn BS. Facilitating adherence to the tobacco use treatment guideline with computer-mediated decision support systems: physician and clinic office manager perspectives. Preventive Medicine. 2005;41:479–87. doi:10.1016/j.ypmed.2004.11.026.
Patterson ES, Doebbeling BN, Fung CH, Militello LG, Anders S, Asch SM. Identifying barriers to the effective use of clinical reminders: bootstrapping multiple methods. Journal of biomedical informatics. 2005;38:189–99. doi:10.1016/j.jbi.2004.11.015.
Rosenbloom ST, et al. Effect of CPOE user interface design on user-initiated access to educational and patient information during clinical care. Journal of the American Medical Informatics Association. 2005;12:458–73.
Rossille D, Laurent J-F, Burgun A. Modelling a decision-support system for oncology using rule-based and case-based reasoning methodologies. International journal of medical informatics. 2005;74:299–306. doi:10.1016/j.ijmedinf.2004.06.005.
Saleem JJ, Patterson ES, Militello LG, Render ML, Orshansky G, Asch SM. Exploring barriers and facilitators to the use of computerized clinical reminders. Journal of the American Medical Informatics Association : JAMIA. 2005;12:438–47. doi:10.1197/jamia.m1777.
Samore MH, et al. Clinical decision support and appropriateness of antimicrobial prescribing: a randomized trial. JAMA. 2005;294:2305–14.
Sequist TD, Gandhi TK, Karson AS, Fiskio JM, Bugbee D, Sperling MR, et al. A Randomized Trial of Electronic Clinical Reminders to Improve Quality of Care for Diabetes and Coronary Artery Disease. Journal of the American Medical Informatics Association : JAMIA. 2005;12:431–7. doi:10.1197/jamia.m1788.
Siika A, Rotich JK, Simiyu CJ, Kigotho EM, Smith FE, Sidle JE, et al. An electronic medical record system for ambulatory care of HIV-infected patients in Kenya. International journal of medical informatics. 2005;74:345–55. doi:10.1016/j.ijmedinf.2005.03.002.
Terraz O, Wietlisbach V, Jeannot J-G, Burnand B, Froehlich F, Gonvers J-J, et al. The EPAGE Internet Guideline as a Decision Support Tool for Determining the Appropriateness of Colonoscopy. Digestion. 2005;71:72–7. doi:10.1159/000084522.
Tierney WM, Overhage JM, Murray MD, Harris LE, Zhou X-H, Eckert GJ, et al. Can computer-generated evidence-based care suggestions enhance evidence-based management of asthma and chronic obstructive pulmonary disease? A randomized, controlled trial. Health services research. 2005;40:477–97. doi:10.1111/j.1475-6773.2005.0t369.x.
Warburton RN. Preliminary outcomes and cost-benefit analysis of a community hospital emergency department screening and referral program for patients aged 75 or more. Int J Health Care Qual Assur Inc Leadersh Health Serv. 2005;18:474–84. doi:10.1108/09526860510619453.
Westbrook JI, Gosling A, Westbrook MT. Use of point-of-care online clinical evidence by junior and senior doctors in New South Wales public hospitals. Internal medicine journal. 2005;35:399–404. doi:10.1111/j.1445-5994.2005.00836.x.

(continued)

List of primary studies (continued)

**Reference**
Zheng K, Padman R, Johnson MP, Diamond HS. Understanding technology adoption in clinical care: Clinician adoption behavior of a point-of-care reminder system. International journal of medical informatics. 2005;74:535–43. doi:10.1016/j.ijmedinf.2005.03.007.
Abarca J, Malone DC, Skrepnek GH, Rehfeld RA, Murphy JE, Grizzle AJ, et al. Community pharmacy managers' perception of computerized drug-drug interaction alerts. Journal of the American Pharmacists Association : JAPhA. 2006;46:148–53. doi:10.1331/154434506776180676.
Allen C, Manyika P, Jazayeri D, Rich M, Lesh N, Fraser HSF. AMIA - Rapid deployment of electronic medical records for ARV rollout in rural Rwanda. AMIA … Annual Symposium proceedings. AMIA Symposium. 2006;2006:840.
Bomba D, Land T. The feasibility of implementing an electronic prescribing decision support system: a case study of an Australian public hospital. Australian health review : a publication of the Australian Hospital Association. 2006;30:380–8. doi:10.1071/ah060380.
Bussmann H, Wester CW, Ndwapi N, Vanderwarker C, Gaolathe T, Tirelo G, et al. Hybrid data capture for monitoring patients on highly active antiretroviral therapy (HAART) in urban Botswana. Bulletin of the World Health Organization. 2006;84:127–31. doi:10.2471/blt.04.019307.
Campbell EM, Sittig DF, Ash JS, Guappone KP, Dykstra RH. Types of Unintended Consequences Related to Computerized Provider Order Entry. Journal of the American Medical Informatics Association : JAMIA. 2006;13:547–56. doi:10.1197/jamia.m2042.
Eliasson M, Bastholm P, Forsberg P, Henriksson K, Jacobson L, Nilsson A, Gustafsson LL. Janus computerised prescribing system provides pharmacological knowledge at point of care - design, development and proof of concept. European journal of clinical pharmacology. 2006;62:251–8. doi:10.1007/s00228-006-0114-2.
Epstein RJ, Leung TW, Mak J, Cheung PSY. Utility of a Web-Based Breast Cancer Predictive Algorithm for Adjuvant Chemotherapeutic Decision Making in a Multidisciplinary Oncology Center. Cancer investigation. 2006;24:367–73. doi:10.1080/07357900600705292.
Feldstein AC, Smith DH, Perrin N, Yang X, Rix MM, Raebel MA, et al. Improved Therapeutic Monitoring With Several Interventions: A Randomized Trial. Archives of internal medicine. 2006;166:1848–54. doi:10.1001/archinte.166.17.1848.
Feldstein AC, Smith DH, Perrin N, Yang X, Simon SR, Krall MA, et al. Reducing warfarin medication interactions: an interrupted time series evaluation. Archives of internal medicine. 2006;166:1009–15. doi:10.1001/archinte.166.9.1009.
Glassman PA, Belperio P, Simon B, Lanto AB, Lee M. Exposure to automated drug alerts over time: effects on clinicians' knowledge and perceptions. Medical care. 2006;44:250–6. doi:10.1097/01.mlr.0000199849.08389.91.
Goergen S, Fong C, Dalziel K, Fennessy G. Can an evidence-based guideline reduce unnecessary imaging of road trauma patients with cervical spine injury in the emergency department? Australasian radiology. 2006;50:563–9. doi:10.1111/j.1440-1673.2006.01655.x.
Hegney D, Buikstra E, Chamberlain C, March J, McKay M, Cope G, et al. Nurse discharge planning in the emergency department: a Toowoomba, Australia, study. J Clin Nurs. 2006;15:1033–44. doi:10.1111/j.1365-2702.2006.01405.x.
Im EO, Chee W. Nurses' acceptance of the decision support computer program for cancer pain management. Comput Inform Nurs. 2006;24:95–104.
Jd M, van der Aa A, B P, van der Weijden T, Winkens R, Jl S. MIE - Design and evaluation of a computer reminder system to improve prescribing behaviour of GPs. Studies in health technology and informatics. 2006;124:617–23.
Jiwa M, Skinner P, Coker AO, Shaw LN, Campbell MJ, Thompson J. Implementing referral guidelines: lessons from a negative outcome cluster randomised factorial trial in general practice. BMC family practice. 2006;7:65. doi:10.1186/1471-2296-7-65.
Ko Y, Abarca J, Malone DC, Dare DC, Geraets D, Houranieh A, et al. Practitioners' Views on Computerized Drug—Drug Interaction Alerts in the VA System. Journal of the American Medical Informatics Association : JAMIA. 2006;14:56–64. doi:10.1197/jamia.m2224.
Kuilboer MM, van Wijk MAM, Mosseveld M, van der Does E, Jongste JC de, Overbeek SE, et al. Computed critiquing integrated into daily clinical practice affects physicians' behavior–a randomized clinical trial with AsthmaCritic. Methods of information in medicine. 2006;45:447–54. doi:10.1055/s-0038-1634103.
Lai F, Macmillan J, Daudelin DH, Kent DM. The potential of training to increase acceptance and use of computerized decision support systems for medical diagnosis. Hum Factors. 2006;48:95–108. doi:10.1518/001872006776412306.
Leslie SJ, Hartswood M, Meurig C, McKee SP, Slack R, Procter R, Denvir MA. Clinical decision support software for management of chronic heart failure: Development and evaluation. Computers in biology and medicine. 2006;36:495–506. doi:10.1016/j.compbiomed.2005.02.002.
Lester WT, et al. Randomized controlled trial of an informatics-based intervention to increase statin prescription for secondary prevention of coronary disease. Journal of general internal medicine. 2006;21:22–9.
Lin ND, Martins SB, Chan AS, Coleman RW, Bosworth HB, Oddone EZ, et al. AMIA - Identifying Barriers to Hypertension Guideline Adherence Using Clinician Feedback at the Point of Care. AMIA … Annual Symposium proceedings. AMIA Symposium. 2006;2006:494–8.
MacLean CD, Littenberg B, Gagnon M. Diabetes Decision Support: Initial Experience With the Vermont Diabetes Information System. American Journal of Public Health. 2006;96:593–5. doi:10.2105/AJPH.2005.065391.
Roumie CL, Elasy TA, Greevy R, Griffin MR, Liu X, Stone WJ, et al. Improving Blood Pressure Control through Provider Education, Provider Alerts, and Patient Education. Ann Intern Med. 2006;145:165–75. doi:10.7326/0003-4819-145-3-200608010-00004.

(continued)

List of primary studies (continued)

Reference
Rubin M, Bateman K, Donnelly S, Stoddard GJ, Stevenson KB, Gardner RM, Samore MH. Use of a personal digital assistant for managing antibiotic prescribing for outpatient respiratory tract infections in rural communities. Journal of the American Medical Informatics Association : JAMIA. 2006;13:627–34. doi:10.1197/jamia.m2029.
Shah NR, Seger AC, Seger DL, Fiskio JM, Kuperman GJ, Blumenfeld BH, et al. Improving Acceptance of Computerized Prescribing Alerts in Ambulatory Care. Journal of the American Medical Informatics Association : JAMIA. 2006;13:5–11. doi:10.1197/jamia.m1868.
Shegog R, Bartholomew LK, Sockrider M, Czyzewski DI, Pilney S, Mullen PD, Abramson SL. Computer-based decision support for pediatric asthma management: description and feasibility of the Stop Asthma Clinical System. Health informatics journal. 2006;12:259–73. doi:10.1177/1460458206069761.
Simon SR, Smith DH, Feldstein AC, Perrin N, Yang X, Zhou Y, et al. Computerized Prescribing Alerts and Group Academic Detailing to Reduce the Use of Potentially Inappropriate Medications in Older People. Journal of the American Geriatrics Society. 2006;54:963–8. doi:10.1111/j.1532-5415.2006.00734.x.
Sittig DF, Krall MA, Dykstra RH, Russell A, Chin HL. A survey of factors affecting clinician acceptance of clinical decision support. BMC medical informatics and decision making. 2006;6:6. doi:10.1186/1472-6947-6-6.
Thomas RE, Croal BL, Ramsay C, Eccles M, Grimshaw J. Effect of enhanced feedback and brief educational reminder messages on laboratory test requesting in primary care: a cluster randomised trial. Lancet (London, England). 2006;367:1990–6. doi:10.1016/S0140-6736(06)68888-0.
Ziemer DC, Doyle JP, Barnes CS, Branch WT, Cook CB, El-Kebbi IM, et al. An Intervention to Overcome Clinical Inertia and Improve Diabetes Mellitus Control in a Primary Care Setting: Improving Primary Care of African Americans With Diabetes (IPCAAD) 8. Archives of internal medicine. 2006;166:507–13. doi:10.1001/archinte.166.5.507.
Allen C, Jazayeri D, Miranda J, Biondich PG, Mamlin BW, Wolfe BA, et al. MedInfo - Experience in implementing the openMRS medical record system to support hiv treatment in Rwanda. Studies in health technology and informatics. 2007;129:382–6.
Ash JS, Sittig DF, Dykstra RH, Guappone KP, Carpenter JD, Seshadri V. Categorizing the unintended sociotechnical consequences of computerized provider order entry. International journal of medical informatics. 2007;76:21–7. doi:10.1016/j.ijmedinf.2006.05.017.
Avery AJ, Savelyich BSP, Sheikh A, Morris C, Bowler I, Teasdale S. Improving general practice computer systems for patient safety: qualitative study of key stakeholders. Quality & safety in health care. 2007;16:28–33. doi:10.1136/qshc.2006.018192.
Cleveringa FG, Gorter KJ, van den Donk M, Pijman PL, Rutten GE. Task Delegation and Computerized Decision Support Reduce Coronary Heart Disease Risk Factors in Type 2 Diabetes Patients in Primary Care. Diabetes Technology & Therapeutics. 2007;9:473–81. doi:10.1089/dia.2007.0210.
Leslie SJ, Denvir MA. Clinical decision support software for chronic heart failure. Critical pathways in cardiology. 2007;6:121–6. doi:10.1097/hpc.0b013e31812da7cc.
Martens JD, van der Weijden T, Severens JL, Clercq PA de, Bruijn DP de, Kester ADM, Winkens R. The effect of computer reminders on GPs' prescribing behaviour: a cluster-randomised trial. International journal of medical informatics. 2007;76:S403-16. doi:10.1016/j.ijmedinf.2007.04.005.
Roumie CL, Elasy TA, Wallston KA, Pratt S, Greevy RA, Liu X, et al. Clinical inertia: a common barrier to changing provider prescribing behavior. Joint Commission journal on quality and patient safety. 2007;33:277–85. doi:10.1016/s1553-7250(07)33032-8.
Saleem JJ, Patterson ES, Militello LG, Anders S, Falciglia M, Wissman J, et al. Impact of clinical reminder redesign on learnability, efficiency, usability, and workload for ambulatory clinic nurses. Journal of the American Medical Informatics Association : JAMIA. 2007;14:632–40. doi:10.1197/jamia.m2163.
Séroussi B, Bouaud J, Gligorov J, Uzan S. Supporting multidisciplinary staff meetings for guideline-based breast cancer management: a study with OncoDoc2. AMIA Annu Symp Proc. 2007;2007:656–60.
Subramanian S, Hoover S, Gilman B, Field TS, Mutter R, Gurwitz JH. Computerized Physician Order Entry with Clinical Decision Support in Long-Term Care Facilities: Costs and Benefits to Stakeholders. Journal of the American Geriatrics Society. 2007;55:1451–7. doi:10.1111/j.1532-5415.2007.01304.x.
Tierney WM, Rotich JK, Hannan TJ, Siika A, Biondich PG, Mamlin BW, et al. MedInfo - The AMPATH medical record system: Creating, implementing, and sustaining an electronic medical record system to support Hiv/AIDS care in western Kenya. Studies in health technology and informatics. 2007;129:372–6.
Wilson A, Duszynski A, Turnbull D, Beilby J. Investigating patients' and general practitioners' views of computerised decision support software for the assessment and management of cardiovascular risk. Inform Prim Care. 2007;15:33–44. doi:10.14236/jhi.v15i1.642.
Wright M-O, Knobloch MJ, Pecher CA, Mejicano GC, Hall MC. Clinical decision support systems use in Wisconsin. WMJ. 2007;106:126–9.
Agostini JV, Concato J, Inouye SK. Improving Sedative-Hypnotic Prescribing in Older Hospitalized Patients: Provider-Perceived Benefits and Barriers of a Computer-Based Reminder. Journal of general internal medicine. 2008;23:32–6. doi:10.1007/s11606-007-0238-9.

(continued)

List of primary studies (continued)

**Reference**
Boudreaux ED, Bedek KL, Gilles D, Baumann BM, Hollenberg SM, Lord SA, Grissom G. The Dynamic Assessment and Referral System for Substance Abuse (DARSSA): Development, functionality, and end-user satisfaction. Drug and alcohol dependence. 2008;99:37–46. doi:10.1016/j.drugalcdep.2008.06.015.
Chused AE, Kuperman GJ, Stetson PD. AMIA - Alert override reasons: a failure to communicate. AMIA ... Annual Symposium proceedings. AMIA Symposium. 2008;2008:111–5.
Del Fiol G, Haug PJ, Cimino JJ, Narus SP, Norlin C, Mitchell JA. Effectiveness of topic-specific infobuttons: a randomized controlled trial. J Am Med Inform Assoc. 2008;15:752–9. doi:10.1197/jamia.M2725.
DeRenzi B, Lesh N, Parikh TS, Sims C, Maokla W, Chemba M, et al. CHI - E-imci: improving pediatric health care in low-income countries. Proceedings of the SIGCHI Conference on Human Factors in Computing Systems 2008. doi:10.1145/1357054.1357174.
Dunsmuir D, Daniels J, Brouse C, Ford S, Ansermino JM. A knowledge authoring tool for clinical decision support. J Clin Monit Comput. 2008;22:189–98.
Egger Halbeis CB, Epstein RH, Macario A, Pearl RG, Grunwald Z. Adoption of Anesthesia Information Management Systems by Academic Departments in the United States. Anesthesia & Analgesia. 2008;107.
Graham TAD, Bullard MJ, Kushniruk AW, Holroyd BR, Rowe BH. Assessing the Sensibility of Two Clinical Decision Support Systems. Journal of medical systems. 2008;32:361–8. doi:10.1007/s10916-008-9141-0.
Graham TA, Kushniruk AW, Bullard MJ, Holroyd BR, Meurer DP, Rowe BH. How Usability of a Web-Based Clinical Decision Support System Has the Potential to Contribute to Adverse Medical Events. AMIA Annu Symp Proc. 2008;2008:257–61.
Javitt JC, Rebitzer JB, Reisman L. Information technology and medical missteps: Evidence from a randomized trial. Journal of Health Economics. 2008;27:585–602. doi:10.1016/j.jhealeco.2007.10.008.
Lapane KL, Waring ME, Schneider KL, Dubé C, Quilliam BJ. A Mixed Method Study of the Merits of E-Prescribing Drug Alerts in Primary Care. Journal of general internal medicine. 2008;23:442–6. doi:10.1007/s11606-008-0505-4.
Marcy TW, Kaplan B, Connolly S, Michel G, Shiffman RN, Flynn BS. Developing a decision support system for tobacco use counselling using primary care physicians. Informatics in primary care. 2008;16:101–9. doi:10.14236/jhi.v16i2.681.
Martens JD, van der Weijden T, Winkens R, Kester ADM, Geerts P, Evers, Silvia M. A. A., Severens JL. Feasibility and acceptability of a computerised system with automated reminders for prescribing behaviour in primary care. International journal of medical informatics. 2008;77:199–207. doi:10.1016/j.ijmedinf.2007.05.013.
O'Reilly DA, Chaudhari MA, Ballal MS, Ghaneh P, Wu A, Poston GJ. The Oncosurge strategy for the management of colorectal liver metastases - an external validation study. European journal of surgical oncology : the journal of the European Society of Surgical Oncology and the British Association of Surgical Oncology. 2008;34:538–40. doi:10.1016/j.ejso.2007.04.013.
Sard BE, Walsh KE, Doros G, Hannon M, Moschetti W, Bauchner H. Retrospective evaluation of a computerized physician order entry adaptation to prevent prescribing errors in a pediatric emergency department. Pediatrics. 2008;122:782–7. doi:10.1542/peds.2007-3064.
Tamblyn R, Huang A, Taylor L, Kawasumi Y, Bartlett G, Grad R, et al. A Randomized Trial of the Effectiveness of On-demand versus Computer-triggered Drug Decision Support in Primary Care. Journal of the American Medical Informatics Association. 2008;15:430–8. doi:10.1197/jamia.M2606.
Toth-Pal E, Wårdh I, Strender L-E, Nilsson G. A guideline-based computerised decision support system (CDSS) to influence general practitioners management of chronic heart failure. Informatics in primary care. 2008;16:29–39. doi:10.14236/jhi.v16i1.672.
Toth-Pal E, Wårdh I, Strender L-E, Nilsson G. Implementing a clinical decision-support system in practice: a qualitative analysis of influencing attitudes and characteristics among general practitioners. Informatics for health & social care. 2008;33:39–54. doi:10.1080/17538150801956754.
van der Sijs H, Aarts J, van Gelder T, Berg M, Vulto AG. Turning Off Frequently Overridden Drug Alerts: Limited Opportunities for Doing It Safely. Journal of the American Medical Informatics Association : JAMIA. 2008;15:439–48. doi:10.1197/jamia.m2311.
van Wyk JT, van Wijk, Marc A M, Sturkenboom, Miriam C J M, Mosseveld M, Moorman PW, van der Lei J. Electronic alerts versus on-demand decision support to improve dyslipidemia treatment: a cluster randomized controlled trial. Circulation. 2008;117:371–8. doi:10.1161/CIRCULATIONAHA.107.697201.
Varonen H, Kortteisto T, Kaila M. What may help or hinder the implementation of computerized decision support systems (CDSSs): a focus group study with physicians. Family practice. 2008;25:162–7. doi:10.1093/fampra/cmn020.
Adshead N, Thomson R. Use of a paediatric early warning system in emergency departments. Emergency Nurse. 2009;17.
Ar HH, Anhøj J, Hellebek A, J E, Bjørn B, Lilja B. Computerised Physician Order Entry (CPOE). Studies in health technology and informatics. 2009;148:159–62.
Bosworth HB, Olsen MK, Dudley TK, Orr M, Goldstein MK, Datta SK, et al. Patient education and provider decision support to control blood pressure in primary care: A cluster randomized trial. American heart journal. 2009;157:450–6. doi:10.1016/j.ahj.2008.11.003.

(continued)

List of primary studies (continued)

Reference
Dowding D, Mitchell N, Randell R, Foster RR, Lattimer V, Thompson C. Nurses' use of computerised clinical decision support systems: a case site analysis. Journal of clinical nursing. 2009;18:1159–67. doi:10.1111/j.1365-2702.2008.02607.x.
Fortuna R, Ross-Degnan D, Campion F, Finkelstein J, Kotch J, Feldstein A, et al. Reducing the Prescribing of Heavily Marketed Medications: A Randomized Controlled Trial. Journal of general internal medicine. 2009;24:897–903. doi:10.1007/s11606-009-1013-x.
Gleeson M, Kellett J, Cowan M, Casey M. An assessment tool for acutely ill medical patients. Br J Nurs. 2009;18:546–50. doi:10.12968/bjon.2009.18.9.42257.
Harrison JD, Masya L, Butow P, Solomon M, Young J, Salkeld G, Whelan T. Implementing patient decision support tools: Moving beyond academia? Patient Education and Counseling. 2009;76:120–5. doi:10.1016/j.pec.2008.12.013.
Kazemi A, Ellenius J, Tofighi S, Salehi A, Eghbalian F, Fors U. CPOE in Iran-A viable prospect? Physicians' opinions on using CPOE in an Iranian teaching hospital. International journal of medical informatics. 2009;78:199–207. doi:10.1016/j.ijmedinf.2008.07.004.
Khajouei R, Jongh D de, Jaspers MWM. MIE - Usability evaluation of a computerized physician order entry for medication ordering. Studies in health technology and informatics. 2009;150:532–6.
Kofoed K, Zalounina A, Andersen O, Lisby G, Paul M, Leibovici L, et al. Performance of the TREAT decision support system in an environment with a low prevalence of resistant pathogens. J Antimicrob Chemother. 2009;63:400–4. doi:10.1093/jac/dkn490.
Lee NJ, et al. The effect of a mobile clinical decision support system on the diagnosis of obesity and overweight in acute and primary care encounters. Advances in Nursing Science. 2009;32:211–21.
Linder JA, et al. An electronic health record–based intervention to improve tobacco treatment in primary care: a cluster-randomized controlled trial. Archives of internal medicine. 2009;169:781–7.
Linder JA, et al. Documentation-based clinical decision support to improve antibiotic prescribing for acute respiratory infections in primary care: a cluster randomized controlled trial. Journal of Innovation in Health Informatics. 2009;17:231–40.
Peiris D, Joshi R, Webster R, Groenestein P, Usherwood T, Heeley E, et al. An Electronic Clinical Decision Support Tool to Assist Primary Care Providers in Cardiovascular Disease Risk Management: Development and Mixed Methods Evaluation. Journal of medical Internet research. 2009;11:1–17. doi:10.2196/jmir.1258.
Peleg M, Shachak A, Wang D, Karnieli E. Using multi-perspective methodologies to study users' interactions with the prototype front end of a guideline-based decision support system for diabetic foot care. International journal of medical informatics. 2009;78:482–93. doi:10.1016/j.ijmedinf.2009.02.008.
Russ AL, Saleem JJ, McManus MS, Zillich AJ, Doebbeling BN. Computerized Medication Alerts and Prescriber Mental Models: Observing Routine Patient Care. Proceedings of the Human Factors and Ergonomics Society Annual Meeting. 2009;53:655–9. doi:10.1177/154193120905301105.
Russ AL, Zillich AJ, McManus MS, Doebbeling BN, Saleem JJ. AMIA - A human factors investigation of medication alerts: barriers to prescriber decision-making and clinical workflow. AMIA ... Annual Symposium proceedings. AMIA Symposium. 2009;2009:548–52.
Sheehan B, Kaufman DR, Stetson PD, Currie LM. AMIA - Cognitive Analysis of Decision Support for Antibiotic Prescribing at the Point of Ordering in a Neonatal Intensive Care Unit. AMIA ... Annual Symposium proceedings. AMIA Symposium. 2009;2009:584–8.
Trivedi MH, Daly EJ, Kern JK, Grannemann BD, Sunderajan P, Claassen CA. Barriers to implementation of a computerized decision support system for depression: An observational report on lessons learned in "real world" clinical settings. BMC medical informatics and decision making. 2009;9:6. doi:10.1186/1472-6947-9-6.
Weber S, Crago EA, Sherwood PR, Smith T. Practitioner approaches to the integration of clinical decision support system technology in critical care. J Nurs Adm. 2009;39:465–9. doi:10.1097/NNA.0b013e3181bd5fc2.
Weingart SN, Massagli MP, Cyrulik A, Isaac T, Morway L, Sands DZ, Weissman JS. Assessing the value of electronic prescribing in ambulatory care: a focus group study. International journal of medical informatics. 2009;78:571–8. doi:10.1016/j.ijmedinf.2009.03.007.
Weingart SN, Simchowitz B, Shiman L, Brouillard D, Cyrulik A, Davis RB, et al. Clinicians' assessments of electronic medication safety alerts in ambulatory care. Archives of internal medicine. 2009;169:1627–32. doi:10.1001/archinternmed.2009.300.
Wilson A, Opolski M. Identifying barriers to implementing a cardiovascular computerised decision support system (CDSS): a Delphi survey. Inform Prim Care. 2009;17:23–33. doi:10.14236/jhi.v17i1.711.
Amoroso C, Akimana B, Wise B, Fraser HSF. MedInfo - Using electronic medical records for HIV care in rural Rwanda. Studies in health technology and informatics. 2010;160:337–41.
Bell LM, Grundmeier RW, Localio R, Zorc JJ, Fiks AG, Zhang X, et al. Electronic health record-based decision support to improve asthma care: a cluster-randomized trial. Pediatrics. 2010;125:e770-7. doi:10.1542/peds.2009-1385.
Bourgeois FC, others. Impact of a computerized template on antibiotic prescribing for acute respiratory infections in children and adolescents. Clinical pediatrics. 2010;49:976–83.

(continued)

List of primary studies (continued)

Reference
Cho SH, Jeong JW, Park H-W, Pyun B-Y, Chang S-I, Moon H-B, et al. Effectiveness of A Computer-Assisted Asthma Management Program on Physician Adherence to Guidelines. The Journal of asthma : official journal of the Association for the Care of Asthma. 2010;47:680–6. doi:10.3109/02770903.2010.481342.
Co JP, others. Electronic health record decision support and quality of care for children with ADHD. Pediatrics. 2010;126:239–46.
Davis AM, Cannon M, Ables AZ, Bendyk H. Using the Electronic Medical Record to Improve Asthma Severity Documentation and Treatment Among Family Medicine Residents. Family medicine. 2010;42:334–7.
Drescher F, Chandrika S, Weir ID, Weintraub JT, Berman L, Lee R, et al. Effectiveness and acceptability of a computerized decision support system using modified Wells criteria for evaluation of suspected pulmonary embolism. Annals of emergency medicine. 2010;57:613–21. doi:10.1016/j.annemergmed.2010.09.018.
Goud R, van Engen-Verheul M, Keizer NF de, Bal R, Hasman A, Hellemans IM, et al. The effect of computerized decision support on barriers to guideline implementation: a qualitative study in outpatient cardiac rehabilitation. Int J Med Inform (Shannon, Ireland). 2010;79:430–7. doi:10.1016/j.ijmedinf.2010.03.001.
Holden RJ. Physicians' beliefs about using EMR and CPOE: In pursuit of a contextualized understanding of health IT use behavior. International journal of medical informatics. 2010;79:71–80. doi:10.1016/j.ijmedinf.2009.12.003.
Hor CP, O'Donnell JM, Murphy AW, O'Brien T, Kropmans T. General practitioners' attitudes and preparedness towards Clinical Decision Support in e-Prescribing (CDS-eP) adoption in the West of Ireland: a cross sectional study. BMC medical informatics and decision making. 2010;10:2. doi:10.1186/1472-6947-10-2.
Horn SD, Sharkey SS, Hudak S, Gassaway J, James R, Spector WD. Pressure ulcer prevention in long-term-care facilities: a pilot study implementing standardized nurse aide documentation and feedback reports. Advances in skin & wound care. 2010;23:120–31. doi:10.1097/01.asw.0000363516.47512.67.
Khajouei R, Peek N, Wierenga PC, Kersten MJ, Jaspers MWM. Effect of predefined order sets and usability problems on efficiency of computerized medication ordering. International journal of medical informatics. 2010;79:690–8. doi:10.1016/j.ijmedinf.2010.08.001.
Kim H, Choi J, Thompson SB, Meeker L, Dykes PC, Goldsmith D, Ohno-Machado L. Automating pressure ulcer risk assessment using documented patient data. International journal of medical informatics. 2010;79:840–8. doi:10.1016/j.ijmedinf.2010.08.005.
McDermott L, Yardley L, Little P, Ashworth M, Gulliford M. Developing a computer delivered, theory based intervention for guideline implementation in general practice. BMC family practice. 2010;11:90. doi:10.1186/1471-2296-11-90.
McLaughlin D, et al. Office-based interventions for recognizing abnormal pediatric blood pressures. Clinical pediatrics. 2010;49:355–62.
Noormohammad SF, Mamlin BW, Biondich PG, McKown B, Kimaiyo S, Were MC. Changing course to make clinical decision support work in an HIV clinic in Kenya. International journal of medical informatics. 2010;79:204–10. doi:10.1016/j.ijmedinf.2010.01.002.
Russ AL, Saleem JJ, McManus MS, Frankel RM, Zillich AJ. The Workflow of Computerized Medication Ordering in Primary Care is Not Prescriptive. Proceedings of the Human Factors and Ergonomics Society Annual Meeting. 2010;54:840–4. doi:10.1177/154193121005401207.
Schnipper JL, et al. Effects of documentation-based decision support on chronic disease management. Am J Manag Care. 2010;16:SP72-81.
Svoronos T, Mjungu D, Dhadialla P, Luk R, Zue C, Jackson J, Lesh N. CommCare: Automated Quality Improvement To Strengthen Community-Based Health. 2010.
Trafton JA, Martins SB, Michel M, Lewis ET, Wang D, Combs A, et al. Evaluation of the Acceptability and Usability of a Decision Support System to Encourage Safe and Effective Use of Opioid Therapy for Chronic, Noncancer Pain by Primary Care Providers. Pain medicine (Malden, Mass.). 2010;11:575–85. doi:10.1111/j.1526-4637.2010.00818.x.
van der Sijs H, van Gelder T, Vulto AG, Berg M, Aarts J. Understanding handling of drug safety alerts: a simulation study. International journal of medical informatics. 2010;79:361–9. doi:10.1016/j.ijmedinf.2010.01.008.
Venkat A, Hunter R, Hegde GG, Chan-Tompkins NH, Chuirazzi DM, Szczesiul JM. Perceptions of Participating Emergency Nurses Regarding an ED Seasonal Influenza Vaccination Program. Journal of emergency nursing. 2010;38:22–9. doi:10.1016/j.jen.2010.08.015.
Were MC, Shen C, Bwana M, Emenyonu N, Musinguzi N, Nkuyahaga F, et al. Creation and evaluation of EMR-based paper clinical summaries to support HIV-care in Uganda, Africa. International journal of medical informatics. 2010;79:90–6. doi:10.1016/j.ijmedinf.2009.11.006.
Williams LK, et al. A cluster-randomized trial to provide clinicians inhaled corticosteroid adherence information for their patients with asthma. J Allergy Clin Immunol. 2010;126:225-231, 31.e1-31.e4.
Alamo S, Wagner GJ, Sunday P, Wanyenze RK, Ouma J, Kamya MR, et al. Electronic Medical Records and Same Day Patient Tracing Improves Clinic Efficiency and Adherence to Appointments in a Community Based HIV/AIDS Care Program, in Uganda. AIDS and behavior. 2011;16:368–74. doi:10.1007/s10461-011-9996-9.
Armstrong KA, Liu F, Seymour AK, Mazhani L, Littman-Quinn R, Fontelo P, Kovarik CL. Evaluation of txt2MEDLINE and Development of Short Messaging Service–Optimized, Clinical Practice Guidelines in Botswana. Telemedicine journal and e-health : the official journal of the American Telemedicine Association. 2011;18:14–7. doi:10.1089/tmj.2011.0014.

(continued)

List of primary studies (continued)

Reference
Ash JS, Sittig DF, Wright A, et al. Clinical decision support in small community practice settings: a case study. J Am Med Inform Assoc. 2011;18:879–82. doi:10.1136/amiajnl-2010-000013.
Atlas SJ, others. A cluster-randomized trial of a primary care informatics-based system for breast cancer screening. Journal of general internal medicine. 2011;26:154–61.
Baysari MT, Westbrook JI, Richardson KL, Day RO. The influence of computerized decision support on prescribing during ward-rounds: are the decision-makers targeted? Journal of the American Medical Informatics Association : JAMIA. 2011;18:754–9. doi:10.1136/amiajnl-2011-000135.
Bouaud J, Séroussi B. Revisiting the EBM decision model to formalize non-compliance with computerized CPGs: results in the management of breast cancer with OncoDoc2. AMIA ... Annual Symposium proceedings / AMIA Symposium. AMIA Symposium. 2011;2011:125–34.
Bowen S, Johnson K, Reed MH, Zhang L, Curry L. The Effect Of Incorporating Guidelines Into a Computerized Order Entry System for Diagnostic Imaging. Journal of the American College of Radiology : JACR. 2011;8:251–8. doi:10.1016/j.jacr.2010.11.020.
Caldon LJ, Collins KA, Reed MW, Sivell S, Austoker J, Clements AM, et al. Clinicians' concerns about decision support interventions for patients facing breast cancer surgery options: understanding the challenge of implementing shared decision-making. Health Expectations. 2011;14:133–46. doi:10.1111/j.1369-7625.2010.00633.x.
Campion TR, May AK, Waitman LR, Ozdas A, Lorenzi NM, Gadd CS. Characteristics and effects of nurse dosing over-rides on computer-based intensive insulin therapy protocol performance. Journal of the American Medical Informatics Association : JAMIA. 2011;18:251–8. doi:10.1136/amiajnl-2011-000129.
Campion TR, Waitman LR, Lorenzi NM, May AK, Gadd CS. Barriers and Facilitators to the Use of Computer-based Intensive Insulin Therapy. International journal of medical informatics. 2011;80:863–71. doi:10.1016/j.ijmedinf.2011.10.003.
Carman MJ, Phipps J, Raley J, Li S, Thornlow DK. Use of a Clinical Decision Support Tool to improve guideline adherence for the treatment of methicillin-resistant Staphylococcus aureus: Skin and Soft Tissue Infections. Advanced emergency nursing journal. 2011;33:252–66. doi:10.1097/tme.0b013e31822610d1.
Chan J, Shojania KG, Easty AC, Etchells E. Usability evaluation of order sets in a computerised provider order entry system. BMJ quality & safety. 2011;20:932–40. doi:10.1136/bmjqs.2010.050021.
Curry L, Reed MH. Electronic decision support for diagnostic imaging in a primary care setting. Journal of the American Medical Informatics Association : JAMIA. 2011;18:267–70. doi:10.1136/amiajnl-2011-000049.
Day RO, Roffe D, Richardson KL, Baysari MT, Brennan N, Beveridge S, et al. Implementing electronic medication management at an Australian teaching hospital. The Medical journal of Australia. 2011;195:498–502. doi:10.5694/mja11.10451.
Duke J, Bolchini D. A successful model and visual design for creating context-aware drug-drug interaction alerts. AMIA ... Annual Symposium proceedings. AMIA Symposium. 2011;2011:339–48.
Fossum M, Alexander GL, Ehnfors M, Ehrenberg A. Effects of a computerized decision support system on pressure ulcers and malnutrition in nursing homes for the elderly. International journal of medical informatics. 2011;80:607–17. doi:10.1016/j.ijmedinf.2011.06.009.
Fossum M, Ehnfors M, Fruhling AL, Ehrenberg A. An Evaluation of the Usability of a Computerized Decision Support System for Nursing Homes. Applied clinical informatics. 2011;2:420–36. doi:10.4338/aci-2011-07-ra-0043.
Hoeksema LJ, Bazzy-Asaad A, Lomotan EA, Edmonds D, Ramirez-Garnica G, Shiffman RN, Horwitz LI. Accuracy of a computerized clinical decision-support system for asthma assessment and management. Journal of the American Medical Informatics Association : JAMIA. 2011;18:243–50. doi:10.1136/amiajnl-2010-000063.
Lapane KL, Rosen RK, Dubé C. Perceptions of e-prescribing efficiencies and inefficiencies in ambulatory care. International journal of medical informatics. 2011;80:39–46. doi:10.1016/j.ijmedinf.2010.10.018.
Lesselroth BJ, Yang J, McConnachie J, Brenk TM, Winterbottom L. Addressing the sociotechnical drivers of quality improvement: a case study of post-operative DVT prophylaxis computerised decision support. BMJ quality & safety. 2011;20:381–9. doi:10.1136/bmjqs.2010.042689.
Loo TS, Davis RB, Lipsitz LA, Irish J, Bates CK, Agarwal K, et al. Electronic Medical Record Reminders and Panel Management to Improve Primary Care of Elderly Patients. Archives of internal medicine. 2011;171:1552–8. doi:10.1001/archinternmed.2011.394.
Millery M, Shelley D, Wu D, Ferrari P, Tseng TY, Kopal H. Qualitative evaluation to explain success of multifaceted technology-driven hypertension intervention. The American journal of managed care. 2011;17:SP95-102.
O'Connor PJ, et al. Impact of electronic health record clinical decision support on diabetes care: a randomized trial. Ann Fam Med. 2011;9:12–21.
O'Sullivan DM, Doyle JS, Michalowski WJ, Wilk SA, Farion KJ, Kuziemsky CE. Assessing the Motivation of MDs to use Computer-based Support at the Point-of-Care in the Emergency Department. AMIA Annu Symp Proc. 2011;2011:1045–54.

(continued)

List of primary studies (continued)

Reference
Peek N, Goud R, Keizer N de, van Engen-Verheul M, Kemps H, Hasman A. CARDSS: Development and Evaluation of a Guideline Based Decision Support System for Cardiac Rehabilitation. In: Peleg M, Lavrač N, Combi C, editors; 2011; Berlin, Heidelberg. Berlin, Heidelberg: Springer Berlin Heidelberg; 2011. p. 109–118.
Peiris D, Usherwood T, Weeramanthri T, Cass A, Patel A. New tools for an old trade: a socio-technical appraisal of how electronic decision support is used by primary care practitioners. Sociology of health & illness. 2011;33:1002–18. doi:10.1111/j.1467-9566.2011.01361.x.
Peleg M, Lavrač N, Combi C, editors. Artificial Intelligence in Medicine. Berlin, Heidelberg: Springer Berlin Heidelberg; 2011.
Pirnejad H, Niazkhani Z, Aarts J, Bal R. MIE - What Makes an Information System More Preferable for Clinicians? a Qualitative Comparison of Two Systems. Studies in health technology and informatics. 2011;169:392–6.
Robertson J, Moxey AJ, Newby D, Gillies MB, Williamson M, Pearson S-A. Electronic information and clinical decision support for prescribing: state of play in Australian general practice. Family practice. 2011;28:93–101. doi:10.1093/fampra/cmq031.
Shapiro A, Gracy D, Quinones W, Applebaum J, Sarmiento A. Putting guidelines into practice: improving documentation of pediatric asthma management using a decision-making tool. Archives of pediatrics & adolescent medicine. 2011;165:412–8. doi:10.1001/archpediatrics.2011.49.
Were MC, Shen C, Tierney WM, Mamlin JJ, Biondich PG, Li X, et al. Evaluation of computer-generated reminders to improve CD4 laboratory monitoring in sub-Saharan Africa: a prospective comparative study. Journal of the American Medical Informatics Association. 2011;18:150–5. doi:10.1136/jamia.2010.005520.
Wess ML, Saleem JJ, Tsevat J, Luckhaupt SE, Johnston JA, Wise RE, et al. Usability of an Atrial Fibrillation Anticoagulation Decision-Support Tool. Journal of primary care & community health. 2011;2:100–6. doi:10.1177/2150131910387608.
Wipfli R, Bétrancourt M, Guardia A, Lovis C. MIE - A qualitative analysis of prescription activity and alert usage in a computerized physician order entry system. Studies in health technology and informatics. 2011;169:940–4.
Yu K, Sweidan M, Williamson M, Fraser A. Drug interaction alerts in software–what do general practitioners and pharmacists want? The Medical journal of Australia. 2011;195:676–80. doi:10.5694/mja11.10206.
Zachariah M, Phansalkar S, Seidling HM, Neri PM, Cresswell K, Duke J, et al. Development and preliminary evidence for the validity of an instrument assessing implementation of human-factors principles in medication-related decision-support systems–I-MeDeSA. Journal of the American Medical Informatics Association : JAMIA. 2011;18:62–72. doi:10.1136/amiajnl-2011-000362.
Zurovac D, Sudoi RK, Akhwale W, Ndiritu M, Hamer DH, Rowe AK, Snow RW. The effect of mobile phone text-message reminders on Kenyan health workers' adherence to malaria treatment guidelines: a cluster randomised trial. Lancet (London, England). 2011;378:795–803. doi:10.1016/s0140-6736(11)60783-6.
Abramson EL, Patel V, Malhotra S, Pfoh ER, Osorio SN, Cheriff AD, et al. Physician experiences transitioning between an older versus newer electronic health record for electronic prescribing. International journal of medical informatics. 2012;81:539–48. doi:10.1016/j.ijmedinf.2012.02.010.
Ash JS, McCormack JL, Sittig DF, Wright A, McMullen CK, Bates DW. Standard practices for computerized clinical decision support in community hospitals: a national survey. Journal of the American Medical Informatics Association : JAMIA. 2012;19:980–7. doi:10.1136/amiajnl-2011-000705.
Ash JS, Sittig DF, Guappone KP, Dykstra RH, Richardson J, Wright A, et al. Recommended practices for computerized clinical decision support and knowledge management in community settings: a qualitative study. BMC Med Inform Decis Mak. 2012;12:6. doi:10.1186/1472-6947-12-6.
Austen C, Patterson C, Poots A, Green S, Weldring T, Bell D. Using a Local Early Warning Scoring System as a Model for the Introduction of a National System. Acute Medicine Journal. 2012;11:66–73. doi:10.52964/amja.0548.
Bajorek BV, Masood N, Krass I. Development of a computerised antithrombotic risk assessment tool (CARAT) to optimise therapy in older persons with atrial fibrillation. Australas J Ageing. 2012;31:102–9.
Bouaud J, Messai N, Laouénan C, Mentré F, Séroussi B. Elicitating patient patterns of physician non-compliance with breast cancer guidelines using formal concept analysis. Studies in health technology and informatics. 2012;180:477–81.
Boudreaux ED, Bedek KL, Byrne NJ, Baumann BM, Lord SA, Grissom G. The Computer-Assisted Brief Intervention for Tobacco (CABIT) program: a pilot study. Journal of medical Internet research. 2012;14:e163-NA. doi:10.2196/jmir.2074.
Collins IM, Breathnach O, Felle P. Electronic clinical decision support systems attitudes and barriers to use in the oncology setting. Ir J Med Sci. 2012;181:521–5. doi:10.1007/s11845-012-0809-6.
Crosson JC, Schueth AJ, Isaacson N, Bell DS. Early Adopters of Electronic Prescribing Struggle to Make Meaningful Use of Formulary Checks and Medication History Documentation. Journal of the American Board of Family Medicine : JABFM. 2012;25:24–32. doi:10.3122/jabfm.2012.01.100297.
Gupta S, Wan FT, Hall S, Straus SE. An asthma action plan created by physician, educator and patient online collaboration with usability and visual design optimization. Respiration; international review of thoracic diseases. 2012;84:406–15. doi:10.1159/000338112.

(continued)

List of primary studies (continued)

**Reference**
Heselmans A, Aertgeerts B, Donceel P, Geens S, van de Velde S, Ramaekers D. Family Physicians' Perceptions and Use of Electronic Clinical Decision Support During the First Year of Implementation. Journal of medical systems. 2012;36:3677–84. doi:10.1007/s10916-012-9841-3.
Jones C, Wasunna B, Sudoi RK, Githinji S, Snow RW, Zurovac D. "Even if You Know Everything You Can Forget": Health Worker Perceptions of Mobile Phone Text-Messaging to Improve Malaria Case-Management in Kenya. PloS one. 2012;7:e38636-NA. doi:10.1371/journal.pone.0038636.
Kim J, Chae YM, Kim S, Ho SH, Kim HH, Park CB. A Study on User Satisfaction regarding the Clinical Decision Support System (CDSS) for Medication. Healthc Inform Res. 2012;18:35–43. doi:10.4258/hir.2012.18.1.35.
Kortteisto T, Komulainen J, Mäkelä M, Kunnamo I, Kaila M. Clinical decision support must be useful, functional is not enough: a qualitative study of computer-based clinical decision support in primary care. BMC health services research. 2012;12:349. doi:10.1186/1472-6963-12-349.
Kunisch JM. Improving emergency department triage classification with computerized clinical decision support at a pediatric hospital; 2012.
Lee G, Murray A, Bushnell R, Niggemeyer LE. Challenges developing evidence-based algorithms for the trauma reception and resuscitation project. International emergency nursing. 2012;21:129–35. doi:10.1016/j.ienj.2012.01.005.
Li AC, Kannry JL, Kushniruk A, Chrimes D, McGinn T, Edonyabo D, Mann DM. Integrating usability testing and think-aloud protocol analysis with "near-live" clinical simulations in evaluating clinical decision support. International journal of medical informatics. 2012;81:761–72. doi:10.1016/j.ijmedinf.2012.02.009.
Litvin CB, Ornstein SM, Wessell AM, Nemeth LS, Nietert PJ. Adoption of a clinical decision support system to promote judicious use of antibiotics for acute respiratory infections in primary care. International journal of medical informatics. 2012;81:521–6. doi:10.1016/j.ijmedinf.2012.03.002.
Lomotan EA, Hoeksema LJ, Edmonds D, Ramirez-Garnica G, Shiffman RN, Horwitz LI. Evaluating the use of a computerized clinical decision support system for asthma by pediatric pulmonologists. International journal of medical informatics. 2012;81:157–65. doi:10.1016/j.ijmedinf.2011.11.004.
Malo C, Neveu X, Archambault PM, Émond M, Gagnon M-P. Exploring Nurses' Intention to Use a Computerized Platform in the Resuscitation Unit: Development and Validation of a Questionnaire Based on the Theory of Planned Behavior. Interactive Journal of Medical Research. 2012;1:e5-NA. doi:10.2196/ijmr.2150.
Mitchell M, Getchell M, Nkaka M, Msellemu D, van Esch J, Hedt-Gauthier B. Perceived improvement in integrated management of childhood illness implementation through use of mobile technology: qualitative evidence from a pilot study in Tanzania. Journal of health communication. 2012;17:118–27. doi:10.1080/10810730.2011.649105.
Robbins GK, et al. Efficacy of a clinical decision-support system in an HIV practice: a randomized trial. Ann Intern Med. 2012;157:757–66.
Russ AL, Zillich AJ, McManus MS, Doebbeling BN, Saleem JJ. Prescribers' interactions with medication alerts at the point of prescribing: A multi-method, in situ investigation of the human–computer interaction. International journal of medical informatics. 2012;81:232–43. doi:10.1016/j.ijmedinf.2012.01.002.
Schwarz EB, et al. Clinical decision support to promote safe prescribing to women of reproductive age: a cluster-randomized trial. Journal of general internal medicine. 2012;27:831–8.
Séroussi B, Soulet A, Messai N, Laouénan C, Mentré F, Bouaud J. Patient clinical profiles associated with physician non-compliance despite the use of a guideline-based decision support system: a case study with OncoDoc2 using data mining techniques. AMIA Annu Symp Proc. 2012;2012:828–37.
Stevens N, Giannareas AR, Kern V, Viesca A, Fortino-Mullen M, King A, et al. Smart alarms: multivariate medical alarm integration for post CABG surgery patients. In: ; 2012. p. 533–542. doi:10.1145/2110363.2110426.
Tamblyn R, Reidel K, Patel V. Physicians' response to computerised alerts for psychotropic drugs in older persons: a multilevel analysis of the associated alert, patient and physician characteristics. BMJ open 2012. doi:10.1136/bmjopen-2012-001384.
Tang JW, et al. Electronic tools to assist with identification and counseling for overweight patients: a randomized controlled trial. Journal of general internal medicine. 2012;27:933–9.
Walter FM, Morris HC, Humphrys E, Hall PN, Prevost AT, Burrows N, et al. Effect of adding a diagnostic aid to best practice to manage suspicious pigmented lesions in primary care: Randomised controlled trial. BMJ (Clinical research ed.). 2012;345:364–5. doi:10.1136/bmj.e4110.
Wan Q, Makeham M, Zwar N, Petche S. Qualitative evaluation of a diabetes electronic decision support tool: views of users. BMC medical informatics and decision making. 2012;12:61. doi:10.1186/1472-6947-12-61.
Wright A, et al. Improving completeness of electronic problem lists through clinical decision support: a randomized, controlled trial. J Am Med Inform Assoc. 2012;19:555–61.
Zafar N. USABILITY EVALUATION OF A VENDOR'S EMERGENCY DEPARTMENT COMPUTERIZED PROVIDER ORDER ENTRY APPLICATION AT THE; 2012.
Zaidi STR, Marriott JL. Barriers and facilitators to adoption of a web-based antibiotic decision support system. South Med Rev. 2012;5:42–9.

(continued)

List of primary studies (continued)

Reference
Zurovac D, Larson BA, Sudoi RK, Snow RW. Costs and Cost-Effectiveness of a Mobile Phone Text-Message Reminder Programmes to Improve Health Workers' Adherence to Malaria Guidelines in Kenya. PloS one. 2012;7:e52045-NA. doi:10.1371/journal.pone.0052045.
Ballard DW, Rauchwerger AS, Reed ME, Vinson DR, Mark DG, Offerman SR, et al. Emergency physicians' knowledge and attitudes of clinical decision support in the electronic health record: a survey-based study. Academic emergency medicine : official journal of the Society for Academic Emergency Medicine. 2013;20:352–60. doi:10.1111/acem.12109.
Baysari MT, Lehnbom EC, Richardson K, O'Reilly BA, Heywood M, Melocco T. Electronic medication information sources: Understanding the needs and preferences of health professionals. Journal of Pharmacy Practice and Research. 2013;43:288–91. doi:10.1002/j.2055-2335.2013.tb00277.x.
Baysari MT, Oliver K, Egan B, Li L, Richardson KL, Sandaradura I, et al. Audit and feedback of antibiotic use: utilising electronic prescription data. Applied clinical informatics. 2013;4:583–95. doi:10.4338/aci-2013-08-ra-0063.
Baysari MT, Westbrook JI, Egan B, Day RO. MedInfo - Identification of strategies to reduce computerized alerts in an electronic prescribing system using a Delphi approach. Studies in health technology and informatics. 2013;192:8–12.
Beeckman D, Clays E, van Hecke A, Vanderwee K, Schoonhoven L, Verhaeghe S. A multi-faceted tailored strategy to implement an electronic clinical decision support system for pressure ulcer prevention in nursing homes: A two-armed randomized controlled trial. International journal of nursing studies. 2013;50:475–86. doi:10.1016/j.ijnurstu.2012.09.007.
Blank A, Prytherch H, Kaltschmidt J, Krings A, Sukums F, Mensah N, et al. Quality of prenatal and maternal care: Bridging the know-do gap (QUALMAT study): an electronic clinical decision support system for rural Sub-Saharan Africa. BMC medical informatics and decision making. 2013;13:44. doi:10.1186/1472-6947-13-44.
Boutis K, others. Effect of the Low Risk Ankle Rule on the frequency of radiography in children with ankle injuries. CMAJ. 2013;185:E731-8.
Bright TJ. Transforming user needs into functional requirements for an antibiotic clinical decision support system: explicating content analysis for system design. Applied clinical informatics. 2013;4:618–35. doi:10.4338/aci-2013-08-ra-0058.
Buenestado D, Elorz J, Pérez-Yarza EG, Iruetaguena A, Segundo U, Barrena R, Pikatza JM. Evaluating acceptance and user experience of a guideline-based clinical decision support system execution platform. Journal of medical systems. 2013;37:9910. doi:10.1007/s10916-012-9910-7.
Cho I, Park I-S, Kim E, Lee E, Bates DW. Using EHR data to predict hospital-acquired pressure ulcers: A prospective study of a Bayesian Network model. International journal of medical informatics. 2013;82:1059–67. doi:10.1016/j.ijmedinf.2013.06.012.
Choi J, Kim H. Enhancement of decision rules to increase generalizability and performance of the rule-based system assessing risk for pressure ulcer. Applied clinical informatics. 2013;4:251–66. doi:10.4338/aci-2012-12-ra-0056.
Dixon BE, Simonaitis L, Goldberg HS, et al. A pilot study of distributed knowledge management and clinical decision support in the cloud. Artif Intell Med. 2013;59:45–53. doi:10.1016/j.artmed.2013.03.004.
Duke JD, Li X, Dexter P. Adherence to drug-drug interaction alerts in high-risk patients: a trial of context-enhanced alerting. J Am Med Inform Assoc. 2013;20:494–8. doi:10.1136/amiajnl-2012-001073.
Feldstein AC, Schneider JL, Unitan R, et al. Health care worker perspectives inform optimization of patient panel-support tools: a qualitative study. Popul Health Manag. 2013;16:107–19. doi:10.1089/pop.2012.0065.
Forrest CB, others. Improving adherence to otitis media guidelines with clinical decision support and physician feedback. Pediatrics. 2013;131:e1071-81.
Fossum M, Ehnfors M, Svensson E, Hansen LM, Ehrenberg A. Effects of a computerized decision support system on care planning for pressure ulcers and malnutrition in nursing homes: An intervention study. International journal of medical informatics. 2013;82:911–21. doi:10.1016/j.ijmedinf.2013.05.009.
Gonzales R, others. A cluster randomized trial of decision support strategies for reducing antibiotic use in acute bronchitis. JAMA Internal Medicine. 2013;173:267–73.
Horner V, Wet Rautenbach PG de, Mbananga N, Mashamba T, Kwinda H. An e-Health Decision Support System for Improving Compliance of Health Workers to the Maternity Care Protocols in South Africa. Applied clinical informatics. 2013;4:25–36. doi:10.4338/aci-2012-10-ra-0044.
Jung M, Hoerbst A, Hackl WO, Kirrane F, Borbolla D, Jaspers MWM, et al. Attitude of physicians towards automatic alerting in computerized physician order entry systems. A comparative international survey. Methods of information in medicine. 2013;52:99–108. doi:10.3414/me12-02-0007.
Kealey E, Leckman-Westin E, Finnerty M. Impact of four training conditions on physician use of a web-based clinical decision support system. Artificial intelligence in medicine. 2013;59:39–44. doi:10.1016/j.artmed.2013.03.003.
Kline JA, Jones AE, Shapiro NI, Hernandez J, Hogg MM, Troyer JL, Nelson RD. Multicenter, Randomized Trial of Quantitative Pretest Probability to Reduce Unnecessary Medical Radiation Exposure in Emergency Department Patients With Chest Pain and Dyspnea. Circulation. Cardiovascular imaging. 2013;7:66–73. doi:10.1161/circimaging.113.001080.

(continued)

List of primary studies (continued)

**Reference**
Langton JM, Blanch B, Pesa N, Park JM, Pearson S-A. How do medical doctors use a web-based oncology protocol system? A comparison of Australian doctors at different levels of medical training using logfile analysis and an online survey. BMC medical informatics and decision making. 2013;13:82. doi:10.1186/1472-6947-13-82.
McGinn TG, et al. Efficacy of an evidence-based clinical decision support in primary care practices: a randomized clinical trial. JAMA Intern Med. 2013;173:1584–91.
Meulendijk M, Spruit M, Maanen CD, Numans ME, Brinkkemper S, Jansen P. General practitioners' attitudes towards decision-supported prescribing: an analysis of the Dutch primary care sector. Health informatics journal. 2013;19:247–63. doi:10.1177/1460458212472333.
Mitchell M, Hedt-Gauthier B, Msellemu D, Nkaka M, Lesh N. Using electronic technology to improve clinical care – results from a before-after cluster trial to evaluate assessment and classification of sick children according to Integrated Management of Childhood Illness (IMCI) protocol in Tanzania. BMC medical informatics and decision making. 2013;13:95. doi:10.1186/1472-6947-13-95.
Montini T, Schenkel AB, Shelley DR. Feasibility of a computerized clinical decision support system for treating tobacco use in dental clinics. J Dent Educ. 2013;77:458–62. doi:10.1002/j.0022-0337.2013.77.4.tb05491.x.
Orlando LA, Buchanan AH, Hahn SE, Christianson CA, Powell KP, Skinner CS, et al. Development and Validation of a Primary Care-Based Family Health History and Decision Support Program (MeTree). North Carolina medical journal. 2013;74:287–96. doi:10.18043/ncm.74.4.287.
Patapovas A, Dormann H, Sedlmayr B, Kirchner M, Sonst A, Müller F, et al. Medication safety and knowledge-based functions: a stepwise approach against information overload. British journal of clinical pharmacology. 2013;76:14–24. doi:10.1111/bcp.12190.
Rindal DB, et al. Computer-assisted guidance for dental office tobacco-cessation counseling: a randomized controlled trial. Am J Prev Med. 2013;44:260–4.
Scheepers-Hoeks A-MJ, Grouls RJE, Neef C, Ackerman EW, Korsten E. Physicians' responses to clinical decision support on an intensive care unit-Comparison of four different alerting methods. Artificial intelligence in medicine. 2013;59:33–8. doi:10.1016/j.artmed.2013.05.002.
Sedlmayr B, Patapovas A, Kirchner M, Sonst A, Müller F, Pfistermeister B, et al. Comparative evaluation of different medication safety measures for the emergency department: physicians' usage and acceptance of training, poster, checklist and computerized decision support. BMC medical informatics and decision making. 2013;13:79. doi:10.1186/1472-6947-13-79.
Séroussi B, Soulet A, Spano J-P, Lefranc J-P, Cojean-Zelek I, Blaszka-Jaulerry B, et al. Which patients may benefit from the use of a decision support system to improve compliance of physician decisions with clinical practice guidelines: A case study with breast cancer involving data mining. In: Studies in Health Technology and Informatics; 2013. p. 534–538. doi:10.3233/978-1-61499-289-9-534.
Séroussi B, Laouénan C, Gligorov J, Uzan S, Mentré F, Bouaud J. Which breast cancer decisions remain non-compliant with guidelines despite the use of computerised decision support? British journal of cancer. 2013;109:1147–56. doi:10.1038/bjc.2013.453.
Sharkey SS, Hudak S, Horn SD, Barrett R, Spector WD, Limcangco R. Exploratory study of nursing home factors associated with successful implementation of clinical decision support tools for pressure ulcer prevention. Advances in skin & wound care. 2013;26:83–92. doi:10.1097/01.asw.0000426718.59326.bb.
Sheehan B, Nigrovic LE, Dayan PS, Kuppermann N, Ballard DW, Alessandrini EA, et al. Informing the design of clinical decision support services for evaluation of children with minor blunt head trauma in the emergency department. Journal of biomedical informatics. 2013;46:905–13. doi:10.1016/j.jbi.2013.07.005.
Simon SR, Keohane CA, Amato M, Coffey M, Cadet B, Zimlichman E, Bates DW. Lessons learned from implementation of computerized provider order entry in 5 community hospitals: a qualitative study. BMC medical informatics and decision making. 2013;13:67. doi:10.1186/1472-6947-13-67.
Vries AE de, van der Wal MHL, Nieuwenhuis MMW, Jong RM de, van Dijk RB, Jaarsma T, et al. Perceived barriers of heart failure nurses and cardiologists in using clinical decision support systems in the treatment of heart failure patients. BMC medical informatics and decision making. 2013;13:54. doi:10.1186/1472-6947-13-54.
Wilson ECF, Emery J, Kinmonth AL, Prevost AT, Morris HC, Humphrys E, et al. The cost-effectiveness of a novel SIAscopic diagnostic aid for the management of pigmented skin lesions in primary care: a decision-analytic model. Value in health : the journal of the International Society for Pharmacoeconomics and Outcomes Research. 2013;16:356–66. doi:10.1016/j.jval.2012.12.008.
Wilson SJ, Wong D, Clifton D, Fleming S, Way R, Pullinger R, et al. Track and trigger in an emergency department: an observational evaluation study. Emerg Med J. 2013;30:186–91. doi:10.1136/emermed-2011-200499.
Yuan MJ, Finley GM, Long J, et al. Evaluation of user interface and workflow design of a bedside nursing clinical decision support system. Interact J Med Res. 2013;2:e4. doi:10.2196/ijmr.2402.
Zaidi STR, Thursky KA. Using formative evaluation to improve uptake of a web-based tool to support antimicrobial stewardship. Journal of clinical pharmacy and therapeutics. 2013;38:490–7. doi:10.1111/jcpt.12093.
Ahn EK, Cho SY, Shin D, Jang C, Park RW. Differences of reasons for alert overrides on contraindicated co-prescriptions by admitting department. Healthc Inform Res. 2014;20:280–7.

(continued)

List of primary studies (continued)

Reference

Arboe B, Laub RR, Kronborg G, Knudsen JD. Evaluation of the decision support system for antimicrobial treatment, TREAT, in an acute medical ward of a university hospital. Int J Infect Dis. 2014;29:156–61. doi:10.1016/j.ijid.2014.01.004.

Asomaning N, Loftus C. Identification of seniors at risk (ISAR) screening tool in the emergency department: implementation using the plan-do-study-act model and validation results. J Emerg Nurs. 2014;40:357-364 e1. doi:10.1016/j.jen.2013.08.014.

Atlas SJ, others. The medication metronome: A health IT system to improve medication management and laboratory monitoring for chronic diseases. Journal of general internal medicine. 2014;29:S230.

Baysari MT, Westbrook JI, Richardson KL, Day RO. HIC - Optimising computerised alerts within electronic medication management systems: A synthesis of four years of research. Studies in health technology and informatics. 2014;204:1–6.

Bouaud J, Blaszka-Jaulerry B, Zelek L, Spano J-P, Lefranc J-P, Cojean-Zelek I, et al. Health information technology: use it well, or don't! Findings from the use of a decision support system for breast cancer management. AMIA ... Annual Symposium proceedings / AMIA Symposium. AMIA Symposium. 2014;2014:315–24.

Catalani C, Green EP, Owiti P, Keny A, Diero L, Yeung A, et al. A clinical decision support system for integrating tuberculosis and HIV care in Kenya: a human-centered design approach. PloS one. 2014;9:e103205-NA. doi:10.1371/journal.pone.0103205.

Cho I, Slight SP, Nanji KC, Seger DL, Maniam N, Dykes PC, Bates DW. Understanding physicians' behavior toward alerts about nephrotoxic medications in outpatients: a cross-sectional analysis. BMC nephrology. 2014;15:200. doi:10.1186/1471-2369-15-200.

Cornu P, Steurbaut S, Gentens K, van de Velde R, Dupont AG. Pilot evaluation of an optimized context-specific drug-drug interaction alerting system: a controlled pre-post study. Int J Med Inform. 2014;84:617–29.

Cornu P, Steurbaut S, De. Beukeleer M, Putman K, van de. Velde R, Dupont A. Physician's expectations regarding prescribing clinical decision support systems in a Belgian hospital. Acta clinica Belgica. 2014;69:157–64. doi:10.1179/2295333714y.0000000015.

Cresswell K, Bates DW, Williams R, Morrison Z, Slee A, Coleman JJ, et al. Evaluation of medium-term consequences of implementing commercial computerized physician order entry and clinical decision support prescribing systems in two 'early adopter' hospitals. Journal of the American Medical Informatics Association : JAMIA. 2014;21:e194-202. doi:10.1136/amiajnl-2013-002252.

Dalaba MA, Akweongo P, Williams J, Saronga HP, Tonchev P, Sauerborn R, et al. Costs associated with implementation of computer-assisted clinical decision support system for antenatal and delivery care: case study of Kassena-Nankana district of northern Ghana. PloS one. 2014;9:e106416. doi:10.1371/journal.pone.0106416.

Demonchy E, Dufour J-C, Gaudart J, Cervetti E, Michelet P, Poussard N, et al. Impact of a computerized decision support system on compliance with guidelines on antibiotics prescribed for urinary tract infections in emergency departments: a multicentre prospective before-and-after controlled interventional study. The Journal of antimicrobial chemotherapy. 2014;69:2857–63. doi:10.1093/jac/dku191.

Dexheimer JW, Abramo TJ, Arnold DH, Johnson K, Shyr Y, Ye F, et al. Implementation and evaluation of an integrated computerized asthma management system in a pediatric emergency department: a randomized clinical trial. Int J Med Inform. 2014;83:805–13. doi:10.1016/j.ijmedinf.2014.08.007.

Doerr M, Edelman E, Gabitzsch E, Eng C, Teng K. Formative evaluation of clinician experience with integrating family history-based clinical decision support into clinical practice. J Pers Med. 2014;4:115–36. doi:10.3390/jpm4020115.

Eccher C, Seyfang A, Ferro A. Implementation and evaluation of an Asbru-based decision support system for adjuvant treatment in breast cancer. Computer methods and programs in biomedicine. 2014;117:308–21. doi:10.1016/j.cmpb.2014.06.021.

Edelman EA, Lin BK, Doksum T, et al. Evaluation of a novel electronic genetic screening and clinical decision support tool in prenatal clinical settings. Matern Child Health J. 2014;18:1233–45. doi:10.1007/s10995-013-1358-y.

Fiks AG, Mayne SL, Karavite D, DeBartolo E, Grundmeier RW. A shared e-decision support portal for pediatric asthma. The Journal of ambulatory care management. 2014;37:120–6. doi:10.1097/jac.0000000000000025.

Fowler S, Yaeger LH, Yu FB, Doerhoff DE, Schoening P, Kelly B. Electronic health record: integrating evidence- based information at the point of clinical decision making. Journal of the Medical Library Association : JMLA. 2014;102:52–5. doi:10.3163/1536-5050.102.1.010.

Gold DL, Mihalov LK, Cohen DM. Evaluating the Pediatric Early Warning Score (PEWS) system for admitted patients in the pediatric emergency department. Acad Emerg Med. 2014;21:1249–56. doi:10.1111/acem.12514.

Griffey RT, Jeffe DB, Bailey TC. Emergency Physicians' Attitudes and Preferences Regarding Computed Tomography, Radiation Exposure, and Imaging Decision Support. Academic emergency medicine : official journal of the Society for Academic Emergency Medicine. 2014;21:768–77. doi:10.1111/acem.12410.

Hum RS, Cato K, Sheehan B, Patel SJ, Duchon J, DeLaMora P, et al. Developing clinical decision support within a commercial electronic health record system to improve antimicrobial prescribing in the neonatal ICU. Applied clinical informatics. 2014;5:368–87. doi:10.4338/aci-2013-09-ra-0069.

Jacobs J, Weir C, Evans RS, Staes C. Assessment of readiness for clinical decision support to aid laboratory monitoring of immunosuppressive care at U.S. liver transplant centers. Appl Clin Inform. 2014;5:988–1004. doi:10.4338/ACI-2014-08-RA-0060.

(continued)

List of primary studies (continued)

**Reference**
Olsho LE, Spector WD, Williams CS, Rhodes W, Fink RV, Limcangco R, Hurd D. Evaluation of AHRQ's on-time pressure ulcer prevention program: a facilitator-assisted clinical decision support intervention for nursing homes. Medical care. 2014;52:258–66. doi:10.1097/mlr.0000000000000080.
O'Reilly D, Bowen JM, Sebaldt RJ, Petrie A, Hopkins R, Assasi N, et al. Evaluation of a chronic disease management system for the treatment and management of diabetes in primary health care practices in Ontario: an observational study. Ontario health technology assessment series. 2014;14:1–37.
O'Sullivan D, Doyle J, Michalowski W, Wilk S, Thomas R, Farion KJ. Expanding usability analysis with intrinsic motivation concepts to learn about CDSS adoption: a case study. Health Policy and Technology. 2014;3:113–25. doi:10.1016/j.hlpt.2014.02.001.
Peiris D, Williams C, Holbrook R, et al. A web-based clinical decision support tool for primary health care management of back pain: development and mixed methods evaluation. JMIR Res Protoc. 2014;3:e17. doi:10.2196/resprot.3071.
Praveen D, Patel A, Raghu A, Clifford GD, Maulik PK, Abdul AM, et al. SMARTHealth India: Development and Field Evaluation of a Mobile Clinical Decision Support System for Cardiovascular Diseases in Rural India. JMIR mHealth and uHealth. 2014;2:e54-NA. doi:10.2196/mhealth.3568.
Santos M, Tygesen H, Eriksson H, Herlitz J. Clinical decision support system (CDSS)–effects on care quality. International journal of health care quality assurance. 2014;27:707–18. doi:10.1108/ijhcqa-01-2014-0010.
Sesen MB, Peake MD, Banares-Alcantara R, Tse D, Kadir T, Stanley R, et al. Lung Cancer Assistant: a hybrid clinical decision support application for lung cancer care. Journal of The Royal Society Interface. 2014;11:20140534. doi:10.1098/rsif.2014.0534.
Snooks HA, et al. Support and Assessment for Fall Emergency Referrals (SAFER 1): cluster randomised trial of computerised clinical decision support for paramedics. PloS one. 2014;9:e106436.
Tsopra R, Jais J-P, Venot A, Duclos C. Comparison of two kinds of interface, based on guided navigation or usability principles, for improving the adoption of computerized decision support systems: application to the prescription of antibiotics. Journal of the American Medical Informatics Association : JAMIA. 2014;21:e107-16. doi:10.1136/amiajnl-2013-002042.
Zakane SA, Gustafsson LL, Tomson G, Loukanova S, Sié A, Nasiell J, Bastholm-Rahmner P. Guidelines for maternal and neonatal "point of care": Needs of and attitudes towards a computerized clinical decision support system in rural Burkina Faso. International journal of medical informatics. 2014;83:459–69. doi:10.1016/j.ijmedinf.2014.01.013.
Anchala R, others. Evaluation of effectiveness and cost-effectiveness of a clinical decision support system in managing hypertension in resource constrained primary health care settings: results from a cluster randomized trial. J Am Heart Assoc. 2015;4:e001213.
Ash JS, Sittig DF, McMullen CK, Wright A, Bunce A, Mohan V, et al. Multiple perspectives on clinical decision support: a qualitative study of fifteen clinical and vendor organizations clinical decision-making, knowledge support systems, and theory. BMC Med Inform Decis Mak. 2015;15.
Barnes J, Duffy A, Hamnett N, et al. The mersey burns app: evolving a model of validation. Emerg Med J. 2015;32:637–41. doi:10.1136/emermed-2013-203416.
Bouaud J, Spano J-P, Lefranc J-P, Cojean-Zelek I, Blaszka-Jaulerry B, Zelek L, et al. Physicians' Attitudes Towards the Advice of a Guideline-Based Decision Support System: A Case Study with OncoDoc2 in the Management of Breast Cancer Patients. In: Studies in Health Technology and Informatics; 2015. p. 264–269. doi:10.3233/978-1-61499-564-7-264.
Chiang P-C, Glance D, Walker J, Walter FM, Emery J. Implementing a QCancer risk tool into general practice consultations: an exploratory study using simulated consultations with Australian general practitioners. British journal of cancer. 2015;112:S77-83. doi:10.1038/bjc.2015.46.
Chow A, Lye DC, Arah OA. Psychosocial determinants of physicians' acceptance of recommendations by antibiotic computerised decision support systems: A mixed methods study. International journal of antimicrobial agents. 2015;45:295–304. doi:10.1016/j.ijantimicag.2014.10.009.
Cresswell KM, Lee L, Slee A, Coleman J, Bates DW, Sheikh A. Qualitative analysis of vendor discussions on the procurement of Computerised Physician Order Entry and Clinical Decision Support systems in hospitals. BMJ open. 2015;5:e008313. doi:10.1136/bmjopen-2015-008313.
Dalaba MA, Akweongo P, Aborigo RA, Saronga HP, Williams J, Blank A, et al. Cost-effectiveness of clinical decision support system in improving maternal health care in Ghana. PloS one. 2015;10:e0125920-NA. doi:10.1371/journal.pone.0125920.
Dikomitis L, Green T, Macleod U. Embedding electronic decision-support tools for suspected cancer in primary care : a qualitative study of GPs' experiences. Primary health care research & development. 2015;16:548–55. doi:10.1017/s1463423615000109.
Fiks AG, Mayne SL, Karavite D, Suh A, O'Hara R, Localio AR, et al. Parent-reported outcomes of a shared decision-making portal in asthma: a practice-based RCT. Pediatrics. 2015;135:e965-73. doi:10.1542/peds.2014-3167.
Flynn D, Nesbitt DJ, Ford GA, McMeekin P, Rodgers H, Price C, et al. Development of a computerised decision aid for thrombolysis in acute stroke care. BMC Med Inform Decis Mak. 2015;15:1–15. doi:10.1186/s12911-014-0127-1.
Frandes M, Timar B, Tole A, et al. Mobile technology support for clinical decision in diabetic keto-acidosis emergency. In: ; 2015. p. 316–320.

(continued)

List of primary studies (continued)

**Reference**
Ginsburg AS, Delarosa J, Brunette W, Levari S, Sundt M, Larson C, et al. mPneumonia: Development of an Innovative mHealth Application for Diagnosing and Treating Childhood Pneumonia and Other Childhood Illnesses in Low-Resource Settings. PloS one. 2015;10:e0139625-NA. doi:10.1371/journal.pone.0139625.
Heiden K, Sinha M, Böckmann B. Virtual Oncological Networks-IT Support for an Evidence-based, Oncological Health Care Management. In: Studies in Health Technology and Informatics; 2015. p. 953. doi:10.3233/978-1-61499-564-7-953.
Helldén A, Al-Aieshy F, Bastholm-Rahmner P, Bergman U, Gustafsson LL, Höök H, et al. Development of a computerised decisions support system for renal risk drugs targeting primary healthcare. BMJ open. 2015;5:e006775-NA. doi:10.1136/bmjopen-2014-006775.
Hendrix KS, others. Pediatricians' responses to printed clinical reminders: does highlighting prompts improve responsiveness? Academic Pediatrics. 2015;15:158–64.
Johnson R, Evans M, Cramer H, Bennert K, Morris RW, Eldridge S, et al. Feasibility and impact of a computerised clinical decision support system on investigation and initial management of new onset chest pain: a mixed methods study. BMC medical informatics and decision making. 2015;15:71. doi:10.1186/s12911-015-0189-8.
Khan S, McCullagh L, Press A, Kharche M, Schachter A, Pardo S, McGinn T. Formative assessment and design of a complex clinical decision support tool for pulmonary embolism. Evidence-based medicine. 2015;21:7–13. doi:10.1136/ebmed-2015-110214.
Khong PCB, Hoi SY, Holroyd E, Wang W. Nurses' Clinical Decision Making on Adopting a Wound Clinical Decision Support System. Computers, informatics, nursing : CIN. 2015;33:295–305. doi:10.1097/cin.0000000000000164.
Kidney E, Berkman L, Macherianakis A, Morton D, Dowswell G, Hamilton W, et al. Preliminary results of a feasibility study of the use of information technology for identification of suspected colorectal cancer in primary care: the CREDIBLE study. British journal of cancer. 2015;112:S70-6. doi:10.1038/bjc.2015.45.
Kuhn L, Reeves K, Taylor YJ, Tapp H, McWilliams A, Gunter A, et al. Planning for Action: The Impact of an Asthma Action Plan Decision Support Tool Integrated into an Electronic Health Record (EHR) at a Large Health Care System. Journal of the American Board of Family Medicine : JABFM. 2015;28:382–93. doi:10.3122/jabfm.2015.03.140248.
Lugtenberg M, Weenink JW, van der Weijden T, Westert GP, Kool RB. Implementation of multiple-domain covering computerized decision support systems in primary care: a focus group study on perceived barriers. BMC Med Inform Decis Mak. 2015;15:82. doi:10.1186/s12911-015-0205-z.
Lugtenberg M, Pasveer D, van der Weijden T, Westert GP, Kool RB. Exposure to and experiences with a computerized decision support intervention in primary care: results from a process evaluation. BMC family practice. 2015;16:141. doi:10.1186/s12875-015-0364-0.
McNabb M, Chukwu E, Ojo O, Shekhar N, Gill CJ, Salami H, Jega F. Assessment of the quality of antenatal care services provided by health workers using a mobile phone decision support application in northern Nigeria: a pre/post-intervention study. PloS one. 2015;10:e0123940-NA. doi:10.1371/journal.pone.0123940.
Mensah N, Sukums F, Awine T, Meid AD, Williams J, Akweongo P, et al. Impact of an electronic clinical decision support system on workflow in antenatal care: the QUALMAT eCDSS in rural health care facilities in Ghana and Tanzania. Global health action. 2015;8:25756. doi:10.3402/gha.v8.25756.
Murphy DR, Wu L, Thomas EJ, Forjuoh SN, Meyer AND, Singh H. Electronic Trigger-Based Intervention to Reduce Delays in Diagnostic Evaluation for Cancer: A Cluster Randomized Controlled Trial. Journal of clinical oncology : official journal of the American Society of Clinical Oncology. 2015;33:3560–7. doi:10.1200/jco.2015.61.1301.
Payne TH, Hines LE, Chan RC, Hartman S, Kapusnik-Uner J, Russ AL, et al. Recommendations to improve the usability of drug-drug interaction clinical decision support alerts. J Am Med Inform Assoc. 2015;22:1243–50.
Press A, McCullagh L, Khan S, Schachter A, Pardo S, McGinn T. Usability Testing of a Complex Clinical Decision Support Tool in the Emergency Department: Lessons Learned. JMIR Hum Factors. 2015;2:e14. doi:10.2196/humanfactors.4537.
Raghu A, Praveen D, Peiris D, Tarassenko L, Clifford GD. Engineering a mobile health tool for resource-poor settings to assess and manage cardiovascular disease risk: SMARThealth study. BMC medical informatics and decision making. 2015;15:36. doi:10.1186/s12911-015-0148-4.
Saronga HP, Dalaba MA, Dong H, Leshabari MT, Sauerborn R, Sukums F, et al. Cost of installing and operating an electronic clinical decision support system for maternal health care: case of Tanzania rural primary health centres. BMC health services research. 2015;15:132. doi:10.1186/s12913-015-0780-9.
Semler MW, et al. An electronic tool for the evaluation and treatment of sepsis in the ICU: a randomized controlled trial. Critical Care Medicine. 2015;43:1595.
Shalom E, Shahar Y, Parmet Y, Lunenfeld E. A multiple-scenario assessment of the effect of a continuous-care, guideline-based decision support system on clinicians' compliance to clinical guidelines. International journal of medical informatics. 2015;84:248–62. doi:10.1016/j.ijmedinf.2015.01.004.

(continued)

List of primary studies (continued)

**Reference**
Shao AF, Rambaud-Althaus C, Samaka J, Faustine AF, Perri-Moore S, Swai N, et al. New Algorithm for Managing Childhood Illness Using Mobile Technology (ALMANACH): A Controlled Non-Inferiority Study on Clinical Outcome and Antibiotic Use in Tanzania. PloS one. 2015;10:e0132316-NA. doi:10.1371/journal.pone.0132316.
Shao AF, Rambaud-Althaus C, Swai N, Kahama-Maro J, Genton B, D'Acremont V, Pfeiffer C. Can smartphones and tablets improve the management of childhood illness in Tanzania? A qualitative study from a primary health care worker's perspective. BMC health services research. 2015;15:135. doi:10.1186/s12913-015-0805-4.
Shemeikka T, Bastholm-Rahmner P, Elinder C-G, Vég A, Törnqvist E, Cornelius B, Korkmaz S. A health record integrated clinical decision support system to support prescriptions of pharmaceutical drugs in patients with reduced renal function: design, development and proof of concept. International journal of medical informatics. 2015;84:387–95. doi:10.1016/j.ijmedinf.2015.02.005.
Stockwell MS, et al. Registry-linked electronic influenza vaccine provider reminders: a cluster-crossover trial. Pediatrics. 2015;135:e75-82.
Sukums F, Mensah N, Mpembeni R, et al. Promising adoption of an electronic clinical decision support system for antenatal and intrapartum care in rural primary healthcare facilities in sub-Saharan Africa: the QUALMAT experience. Int J Med Inform. 2015;84:647–57. doi:10.1016/j.ijmedinf.2015.05.002.
Tamblyn R, Ernst P, Winslade N, Huang A, Grad R, Platt RW, et al. Evaluating the impact of an integrated computer-based decision support with person-centered analytics for the management of asthma in primary care: a randomized controlled trial. Journal of the American Medical Informatics Association : JAMIA. 2015;22:773–83. doi:10.1093/jamia/ocu009.
Vedanthan R, Blank E, Tuikong N, Kamano JH, Misoi L, Tulienge D, et al. Usability and feasibility of a tablet-based Decision-Support and Integrated Record-keeping (DESIRE) tool in the nurse management of hypertension in rural western Kenya. International journal of medical informatics. 2015;84:207–19. doi:10.1016/j.ijmedinf.2014.12.005.
Winkelmann RR, Yoo J, Tucker N, White R, Rigel DS. Impact of Guidance Provided by a Multispectral Digital Skin Lesion Analysis Device Following Dermoscopy on Decisions to Biopsy Atypical Melanocytic Lesions. The Journal of clinical and aesthetic dermatology. 2015;8:21–4.
Yadav K, Chamberlain JM, Lewis VR, Abts N, Chawla S, Hernandez A, et al. Designing Real-time Decision Support for Trauma Resuscitations. Academic emergency medicine : official journal of the Society for Academic Emergency Medicine. 2015;22:1076–84. doi:10.1111/acem.12747.
Alagiakrishnan K, Wilson P, Sadowski C, et al. Physicians' use of computerized clinical decision supports to improve medication management in the elderly; the Seniors Medication Alert and Review Technology intervention. Clin Interv Aging. 2016;11:73–81. doi:10.2147/CIA.S94126.
Ballard DW, Vemula R, Chettipally UK, Kene MV, Mark DG, Elms AK, et al. Optimizing clinical decision support in the electronic health record: clinical characteristics associated with the use of a decision tool for disposition of ED patients with pulmonary embolism. Appl Clin Inform. 2016;7:883–98. doi:10.4338/ACI-2016-05-RA-0073.
Beaudoin M, Kabanza F, Nault V, Valiquette L. Evaluation of a machine learning capability for a clinical decision support system to enhance antimicrobial stewardship programs. Artif Intell Med. 2016;68:29–36. doi:10.1016/j.artmed.2016.01.002.
Delgado-Hurtado JJ, Berger A, Bansal AB. Emergency department Modified Early Warning Score association with admission, admission disposition, mortality, and length of stay. J Community Hosp Intern Med Perspect. 2016;6:31456. doi:10.3402/jchimp.v6.31456.
Dixon BE, Alzeer AH, Phillips EO, Marrero DG. Integration of Provider, Pharmacy, and Patient-Reported Data to Improve Medication Adherence for Type 2 Diabetes: A Controlled Before-After Pilot Study. JMIR medical informatics. 2016;4:e4-NA. doi:10.2196/medinform.4739.
Duysburgh E, Temmerman M, Yé M, Williams A, Massawe S, Williams JW, et al. Quality of antenatal and childbirth care in rural health facilities in Burkina Faso, Ghana and Tanzania: an intervention study. Tropical medicine & international health : TM & IH. 2016;21:70–83. doi:10.1111/tmi.12627.
Eckman MH, others. Impact of an atrial fibrillation decision support tool on thromboprophylaxis for atrial fibrillation. American heart journal. 2016;176:17–27.
Freitas DalBen M de, Mendes ET, Moura ML, Rahman DA, Peixoto D, Dos Santos SA, et al. A model-based strategy to control the spread of carbapenem-resistant Enterobacteriaceae: simulate and implement. Infect Control Hosp Epidemiol. 2016;37:1315–22. doi:10.1017/ice.2016.166.
Ginsburg AS, Tawiah Agyemang C, Ambler G, et al. mPneumonia, an innovation for diagnosing and treating childhood pneumonia in low-resource settings: a feasibility, usability and acceptability study in Ghana. PloS one. 2016;11:e0165201. doi:10.1371/journal.pone.0165201.
Jenssen BP, Bryant-Stephens T, Leone FT, Grundmeier RW, Fiks AG. Clinical decision support tool for parental tobacco treatment in primary care. Pediatrics. 2016;137:e20154185. doi:10.1542/peds.2015-4185.
Kanagasundaram NS, Bevan M, Sims A, Heed A, Price D, Sheerin NS. Computerized clinical decision support for the early recognition and management of acute kidney injury: a qualitative evaluation of end-user experience. Clinical kidney journal. 2016;9:57–62. doi:10.1093/ckj/sfv130.
Kappen TH, van Loon K, Kappen MA, van Wolfswinkel L, Vergouwe Y, van Klei WA, et al. Barriers and facilitators perceived by physicians when using prediction models in practice. Journal of clinical epidemiology. 2016;70:136–45. doi:10.1016/j.jclinepi.2015.09.008.

(continued)

List of primary studies (continued)

Reference
Koskela T, Sandström S, Mäkinen J, Liira H. User perspectives on an electronic decision-support tool performing comprehensive medication reviews - a focus group study with physicians and nurses. BMC Med Inform Decis Mak. 2016;16:6. doi:10.1186/s12911-016-0245-z.
Lee J, Gogo A, Tancredi D, Fernandez Y Garcia E, Shaikh U. Improving asthma care in a pediatric resident clinic. BMJ Qual Improv Rep 2016. doi:10.1136/bmjquality.u214746.w6381.
Lee L, Williams R, Sheikh A. How does joint procurement affect the design, customisation and usability of a hospital ePrescribing system? Health Informatics J. 2016;22:828–38. doi:10.1177/1460458215592915.
Lin F, Pokorny A, Teng C, Dear RF, Epstein RJ. Computational prediction of multidisciplinary team decision-making for adjuvant breast cancer drug therapies: a machine learning approach. BMC cancer. 2016;16:929. doi:10.1186/s12885-016-2972-z.
Litvin CB, Hyer JM, Ornstein SM. Use of Clinical Decision Support to Improve Primary Care Identification and Management of Chronic Kidney Disease (CKD). Journal of the American Board of Family Medicine : JABFM. 2016;29:604–12. doi:10.3122/jabfm.2016.05.160020.
Maia JX, Sousa LAP de, Marcolino MS, et al. The impact of a clinical decision support system in diabetes primary care patients in a developing country. Diabetes Technol Ther. 2016;18:258–63. doi:10.1089/dia.2015.0253.
Matiz LA, Robbins-Milne L, Krause MC, Peretz PJ, Rausch JC. Evaluating the Impact of Information Technology Tools to Support the Asthma Medical Home. Clinical pediatrics. 2016;55:165–70. doi:10.1177/0009922815596070.
Mazzaglia G, et al. Effects of a computerized decision support system in improving pharmacological management in high-risk cardiovascular patients: A cluster-randomized open-label controlled trial. Health informatics journal. 2016;22:232–47.
McDonald MV, et al. Outcomes of clinical decision support (CDSS) and correlates of CDSS use for home care patients with high medication regimen complexity: a randomized trial. J Eval Clin Pract. 2016;22:10–9.
Meeker D, Linder JA, Fox CR, Friedberg MW, Persell SD, Goldstein NJ, et al. Effect of Behavioral Interventions on Inappropriate Antibiotic Prescribing Among Primary Care Practices: A Randomized Clinical Trial. JAMA. 2016;315:562–70. doi:10.1001/jama.2016.0275.
Meyer AND, Murphy DR, Singh H. Communicating Findings of Delayed Diagnostic Evaluation to Primary Care Providers. Journal of the American Board of Family Medicine : JABFM. 2016;29:469–73. doi:10.3122/jabfm.2016.04.150363.
Mozaffar H, Cresswell KM, Lee L, Williams R, Sheikh A, On behalf of the NIHR ePrescribing Programme Team. Taxonomy of delays in the implementation of hospital computerized physician order entry and clinical decision support systems for prescribing: a longitudinal qualitative study. BMC medical informatics and decision making. 2016;16:25. doi:10.1186/s12911-016-0263-x.
Mozaffar H, Williams R, Cresswell K, Morrison Z, Bates DW, Sheikh A. The evolution of the market for commercial computerized physician order entry and computerized decision support systems for prescribing. J Am Med Inform Assoc. 2016;23:349–55. doi:10.1093/jamia/ocv095.
Penkalski MR, Kenneally M. Provider Adherence to Evidence-Based Asthma Guidelines in a Community Health Center. Journal of doctoral nursing practice. 2016;9:128–38. doi:10.1891/2380-9418.9.1.128.
Ramanathan R, Lee N, Duane TM, Gu Z, Nguyen N, Potter TG, et al. Correlation of venous thromboembolism prophylaxis and electronic medical record alerts with incidence among surgical patients. Surgery. 2016;160:1202–10. doi:10.1016/j.surg.2016.04.029.
Santucci W, Day RO, Baysari MT. Evaluation of hospital-wide computerised decision support in an intensive care unit: an observational study. Anaesthesia and intensive care. 2016;44:507–12. doi:10.1177/0310057x1604400403.
Schoemans H, Goris K, Durm RV, Vanhoof J, Wolff D, Greinix H, et al. Development, preliminary usability and accuracy testing of the EBMT 'eGVHD App' to support GvHD assessment according to NIH criteria-a proof of concept. Bone Marrow Transplant. 2016;51:1062–5. doi:10.1038/bmt.2016.26.
Seidling HM, Stützle M, Hoppe-Tichy T, Allenet B, Bedouch P, Bonnabry P, et al. Best practice strategies to safeguard drug prescribing and drug administration: an anthology of expert views and opinions. International journal of clinical pharmacy. 2016;38:362–73. doi:10.1007/s11096-016-0253-1.
Silbernagel G, et al. Electronic Alert System for Improving Stroke Prevention Among Hospitalized Oral-Anticoagulation-Naïve Patients With Atrial Fibrillation: a Randomized Trial. Journal of the American Heart Association. 2016;5:e003776.
Williams PA, Furberg RD, Bagwell JE, LaBresh KA. Usability testing and adaptation of the pediatric cardiovascular risk reduction clinical decision support tool. JMIR Hum Factors. 2016;3:e17. doi:10.2196/humanfactors.5440.
Yang Q, Zimmerman J, Steinfeld A, Carey L, Antaki JF. Investigating the heart pump implant decision process: opportunities for decision support tools to help. In: ; 2016. p. 4477–4488. doi:10.1145/2858036.2858253.
Arts DL, others. Effectiveness and usage of a decision support system to improve stroke prevention in general practice: a cluster randomized controlled trial. PloS one. 2017;12:e0170974.
Ash JS, Chase DA, Wiesen J, Murphy E, Marovich S. AMIA - Studying Readiness for Clinical Decision Support for Worker Health Using the Rapid Assessment Process and Mixed Methods Interviews. AMIA ... Annual Symposium proceedings. AMIA Symposium. 2017;2016:285–94.

(continued)

List of primary studies (continued)

Reference
Ballard ACY, Kessler ME, Scheitel MR, Montori VM, Chaudhry R. Exploring differences in the use of the statin choice decision aid and diabetes medication choice decision aid in primary care. BMC medical informatics and decision making. 2017;17:118. doi:10.1186/s12911-017-0514-5.
Bamidis PD, Konstantinidis ST, Rodrigues PP. Design and Development of a Mobile Decision Support System: Guiding Clinicians Regarding Law in the Practice of Psychiatry in Emergency Department. In: Thessaloniki, Greece: Institute of Electrical and Electronics Engineers Inc; 2017. p. 67–72.
Baysari MT, Del Gigante J, Moran M, Sandaradura I, Li L, Richardson KL, et al. Redesign of computerized decision support to improve antimicrobial prescribing. A controlled before-and-after study. Applied clinical informatics. 2017;8:949–63. doi:10.4338/aci2017040069.
Burdick H, Pino E, Gabel-Comeau D, Gu C, Huang H, Lynn-Palevsky A, et al. Evaluating a sepsis prediction machine learning algorithm in the emergency department and intensive care unit: a before and after comparative study. BioRxiv. 2017:224014. doi:10.1101/224014.
Caballero-Ruiz E, García-Sáez G, Rigla M, Villaplana M, Pons B, Hernando ME, et al. A web-based clinical decision support system for gestational diabetes: Automatic diet prescription and detection of insulin needs. Int. J. Med. Inform. 2017;102:35–49. doi:10.1016/j.ijmedinf.2017.02.014.
Chiang JI, Furler J, Boyle D, Clark M, Manski-Nankervis J-A. Electronic clinical decision support tool for the evaluation of cardiovascular risk in general practice: A pilot study. Australian family physician. 2017;46:764–8.
Chung P, Scandlyn J, Dayan PS, Mistry RD. Working at the intersection of context, culture, and technology: Provider perspectives on antimicrobial stewardship in the emergency department using electronic health record clinical decision support. American journal of infection control. 2017;45:1198–202. doi:10.1016/j.ajic.2017.06.005.
Cresswell KM, Lee L, Mozaffar H, Williams R, Sheikh A, Robertson A, et al. Sustained user engagement in health information technology: the long road from implementation to system optimization of computerized physician order entry and clinical decision support systems for prescribing in hospitals in England. Health Serv Res. 2017;52:1928–57. doi:10.1111/1475-6773.12581.
Cypko MA, Stoehr M, Kozniewski M, Druzdzel MJ, Dietz A, Berliner L, Lemke HU. Validation workflow for a clinical Bayesian network model in multidisciplinary decision making in head and neck oncology treatment. International journal of computer assisted radiology and surgery. 2017;12:1959–70. doi:10.1007/s11548-017-1531-7.
English D, Ankem K, English K. Acceptance of clinical decision support surveillance technology in the clinical pharmacy. Inform Health Soc Care. 2017;42:135–52. doi:10.3109/17538157.2015.1113415.
Gaudioso C, Elkin P. Considerations of human factors in the design and implementation of clinical decision support systems for tumor boards. In: Studies in Health Technology and Informatics; 2017. p. 1324. doi:10.3233/978-1-61499-830-3-1324.
Henshall C, Marzano L, Smith K, Attenburrow MJ, Puntis S, Zlodre J, et al. A web-based clinical decision tool to support treatment decision-making in psychiatry: a pilot focus group study with clinicians, patients and carers. BMC psychiatry. 2017;17:265. doi:10.1186/s12888-017-1406-z.
Kahan NR, others. Large-scale, community-based trial of a personalized drug-related problem rectification system. American Journal of Pharmacy. 2017;9:41–6.
Kempe A, Hurley LP, Cardemil CV, et al. Use of immunization information systems in primary care. Am J Prev Med. 2017;52:173–82. doi:10.1016/j.amepre.2016.07.029.
Khong PCB, Lee LN, Dawang AI. Modeling the Construct of an Expert Evidence-Adaptive Knowledge Base for a Pressure Injury Clinical Decision Support System. Informatics. 2017;4:20-NA. doi:10.3390/informatics4030020.
Kidney E, Greenfield S, Berkman L, Dowswell G, Hamilton W, Wood S, Marshall T. Cancer suspicion in general practice, urgent referral, and time to diagnosis: a population-based GP survey nested within a feasibility study using information technology to flag-up patients with symptoms of colorectal cancer. BJGP open. 2017;1:bjgpopen17X101109-NA. doi:10.3399/bjgpopen17x101109.
Leung S, Zheng WY, Sandhu A, Day RO, Li L, Baysari MT. HIC - Feedback and Training to Improve Use of an Electronic Prescribing System: A Randomised Controlled Trial. Studies in health technology and informatics. 2017;239:63–9.
Liberati E, Ruggiero F, Galuppo L, Gorli M, González-Lorenzo M, Maraldi M, et al. What hinders the uptake of computerized decision support systems in hospitals? A qualitative study and framework for implementation. Implementation science : IS. 2017;12:113. doi:10.1186/s13012-017-0644-2.
Omar A, Ellenius J, Lindemalm S. ITCH - Evaluation of Electronic Prescribing Decision Support System at a Tertiary Care Pediatric Hospital: The User Acceptance Perspective. Studies in health technology and informatics. 2017;234:256–61.
Price M, Davies I, Rusk R, Lesperance M, Weber J. Applying STOPP guidelines in primary care through electronic medical record decision support: randomized control trial highlighting the importance of data quality. JMIR Med Inform. 2017;5:e15. doi:10.2196/medinform.6226.
Pullinger R, Wilson S, Way R, Santos M, Wong D, Clifton D, et al. Implementing an electronic observation and early warning score chart in the emergency department: a feasibility study. Eur J Emerg Med. 2017;24:e11-e16. doi:10.1097/MEJ.0000000000000371.

(continued)

List of primary studies (continued)

**Reference**
Regan ME. Implementing an evidence-based clinical decision support tool to improve the detection, evaluation, and referral patterns of adult chronic kidney disease patients in primary care. Journal of the American Association of Nurse Practitioners. 2017;29:741–53. doi:10.1002/2327-6924.12505.
Séroussi B, Guézennec G, Lamy J-B, Muro N, Larburu N, Sekar BD, et al. Reconciliation of multiple guidelines for decision support: a case study on the multidisciplinary management of breast cancer within the DESIREE project. AMIA Annu Symp Proc. 2017;2017:1527–36.
Spirk D, et al. Electronic alert system for improving appropriate thromboprophylaxis in hospitalized medical patients: a randomized controlled trial. Journal of Thrombosis and Haemostasis. 2017;15:2138–46.
van den Wijngaart LS, Roukema J, Boehmer AL, Brouwer ML, Hugen CAC, Niers LEM, et al. A virtual asthma clinic for children: fewer routine outpatient visits, same asthma control. The European respiratory journal. 2017;50:1700471-NA. doi:10.1183/13993003.00471-2017.
Vandenberg AE, Vaughan CP, Stevens MB, Hastings SN, Powers JS, Markland AD, et al. Improving geriatric prescribing in the ED: a qualitative study of facilitators and barriers to clinical decision support tool use. International journal for quality in health care : journal of the International Society for Quality in Health Care. 2017;29:117–23. doi:10.1093/intqhc/mzw129.
Wannheden C, Hvitfeldt-Forsberg H, Eftimovska E, Westling K, Ellenius J. Boosting Quality Registries with Clinical Decision Support Functionality*. User Acceptance of a Prototype Applied to HIV/TB Drug Therapy. Methods of information in medicine. 2017;56:339–43. doi:10.3414/me16-02-0030.
Yılmaz AA, Ozdemir L. Development and Implementation of the clinical decision support system for patients with cancer and nurses' experiences regarding the system. Int J Nurs Knowl. 2017;28:4–12. doi:10.1111/2047-3095.12099.
Andruchow JE, others. A randomized controlled trial of electronic clinical decision support to reduce unnecessary CT imaging for patients with suspected pulmonary embolism. Canadian Journal of Emergency Medicine. 2018;20:S32-3.
Arts DL, Medlock SK, van Weert HCPM, Wyatt JC, Abu-Hanna A. Acceptance and barriers pertaining to a general practice decision support system for multiple clinical conditions: a mixed methods evaluation. PloS one. 2018;13:e0193187. doi:10.1371/journal.pone.0193187.
Baysari MT, Hardie R-A, Lake R, Richardson L, McCullagh C, Gardo A, Westbrook JI. Longitudinal study of user experiences of a CPOE system in a pediatric hospital. International journal of medical informatics. 2018;109:5–14. doi:10.1016/j.ijmedinf.2017.10.018.
Blanco N, O'Hara LM, Robinson GL, Brown J, Heil E, Brown CH, et al. Health care worker perceptions toward computerized clinical decision support tools for Clostridium difficile infection reduction: a qualitative study at 2 hospitals. Am J Infect Control. 2018;46:1160–6. doi:10.1016/j.ajic.2018.04.204.
Böttiger Y, Laine K, Korhonen T, Lähdesmäki J, Shemeikka T, Julander M, et al. Development and pilot testing of PHARAO-a decision support system for pharmacological risk assessment in the elderly. European journal of clinical pharmacology. 2018;74:365–71. doi:10.1007/s00228-017-2391-3.
Carroll JK, Pulver G, Dickinson LM, Pace WD, Vassalotti JA, Kimminau KS, et al. Effect of 2 Clinical Decision Support Strategies on Chronic Kidney Disease Outcomes in Primary Care: A Cluster Randomized Trial. JAMA Netw Open. 2018;1:e183377. doi:10.1001/jamanetworkopen.2018.3377.
Chua A, Tang SS, Lee LW, Yii DYC, Kong ST, Lee W, et al. Psychosocial determinants of physician acceptance toward an antimicrobial stewardship program and its computerized decision support system in an acute care tertiary hospital. JACCP: JOURNAL OF THE AMERICAN COLLEGE OF CLINICAL PHARMACY. 2018;1:NA-NA. doi:10.1002/jac5.1028.
Conway N, Adamson K, Cunningham S, Smith AE, Nyberg P, Smith BH, et al. Decision Support for Diabetes in Scotland: Implementation and Evaluation of a Clinical Decision Support System. Journal of diabetes science and technology. 2018;12:381–8. doi:10.1177/1932296817729489.
Dagliati A, Sacchi L, Tibollo V, Cogni G, Teliti M, Martinez-Millana A, et al. A dashboard-based system for supporting diabetes care. Journal of the American Medical Informatics Association : JAMIA. 2018;25:538–47. doi:10.1093/jamia/ocx159.
Diaz MC, others. A Provider-Focused Intervention to Promote Optimal Care of Pediatric Patients With Suspected Elbow Fracture. Pediatric Emergency Care. 2018.
Giuliano CA, Binienda J, Kale-Pradhan PB, Fakih MG. "I never would have caught that before": pharmacist perceptions of using clinical decision support for antimicrobial stewardship in the United States. Qual Health Res. 2018;28:745–55. doi:10.1177/1049732317750863.
Hasnie AA, Kumbamu A, Safarova MS, Caraballo PJ, Kullo IJ. A clinical decision support tool for familial hypercholesterolemia based on physician input. Mayo Clin Proc Innov Qual Outcomes. 2018;2:103–12. doi:10.1016/j.mayocpiqo.2018.03.006.
Hirsch T, Soma C, Merced K, et al. It's hard to argue with a computer: investigating psychotherapists' attitudes towards automated evaluation. 2018:559–71.
Holt T, Dalton A, Kirkpatrick S, Hislop J, Marshall T, Fay M, et al. Barriers to a software reminder system for risk assessment of stroke in atrial fibrillation: a process evaluation of a cluster randomised trial in general practice. The British journal of general practice : the journal of the Royal College of General Practitioners. 2018;68:844–51. doi:10.3399/bjgp18x699809.

(continued)

List of primary studies (continued)

**Reference**
Hoonlor A, Charoensawan V, Srisuma S. The clinical decision support system for the snake envenomation in Thailand. In: ; 2018. p. 1–6. doi:10.1109/JCSSE.2018.8683369.
Jindal D, Gupta P, Jha D, Ajay VS, Goenka S, Jacob P, et al. Development of mWellcare: an mHealth intervention for integrated management of hypertension and diabetes in low-resource settings. Global health action. 2018;11:1517930. doi:10.1080/16549716.2018.1517930.
Kharbanda EO, others. Evaluation of an electronic clinical decision support tool for incident elevated BP in adolescents. Academic Pediatrics. 2018;18.
Kim B, Kim K, Lee J, Kim J, Jo YH, Lee JH, et al. Impact of bacteremia prediction rule in CAP: Before and after study. Am J Emerg Med. 2018;36:758–62. doi:10.1016/j.ajem.2017.10.005.
Klingberg A, Wallis LA, Hasselberg M, et al. Teleconsultation using mobile phones for diagnosis and acute care of burn injuries among emergency physicians: mixed-methods study. JMIR Mhealth Uhealth. 2018;6:e11076. doi:10.2196/11076.
Kumar SS, Woodward-Kron R, Frank O, Knieriemen A, Lau P. Patient-directed reminders to improve preventive care in general practice for patients with type 2 diabetes: A proof of concept. Australian journal of general practice. 2018;47:383–8. doi:10.31128/ajgp-10-17-4353.
Lee W-S, Ahn SM, Chung J-W, Kim KO, Kwon K an, Kim Y, et al. Assessing Concordance With Watson for Oncology, a Cognitive Computing Decision Support System for Colon Cancer Treatment in Korea. JCO Clinical Cancer Informatics. 2018:1–8. doi:10.1200/CCI.17.00109.
Liu C, Liu X, Wu F, Xie M, Feng Y, Hu C. Using Artificial Intelligence (Watson for Oncology) for Treatment Recommendations Amongst Chinese Patients with Lung Cancer: Feasibility Study. Journal of medical Internet research. 2018;20:e11087-NA. doi:10.2196/11087.
Masterson Creber RM, Dayan PS, Kuppermann N, Ballard DW, Tzimenatos L, Alessandrini E, et al. Applying the RE-AIM Framework for the evaluation of a clinical decision support tool for pediatric head trauma: a mixed-methods study. Appl Clin Inform. 2018;9:693–703. doi:10.1055/s-0038-1669460.
Meador M, Osheroff JA, Reisler B. Improving Identification and Diagnosis of Hypertensive Patients Hiding in Plain Sight (HIPS) in Health Centers. Joint Commission journal on quality and patient safety. 2018;44:117–29. doi:10.1016/j.jcjq.2017.09.003.
Mullen S, Quinn-Scoggins HD, Nuttall D, Kemp AM. Qualitative analysis of clinician experience in utilising the BuRN Tool (Burns Risk assessment for Neglect or abuse Tool) in clinical practice. Burns. 2018;44:1759–66. doi:10.1016/j.burns.2018.03.013.
Nanji KC, Seger DL, Slight SP, Amato MG, Beeler PE, Her QL, et al. Medication-related clinical decision support alert overrides in inpatients. J Am Med Inform Assoc. 2018;25:476–81.
Paradis M, Stiell I, Atkinson KM, et al. Acceptability of a mobile clinical decision tool among emergency department clinicians: development and evaluation of the Ottawa rules app. JMIR Mhealth Uhealth. 2018;6:e10263. doi:10.2196/10263.
Patel B, Usherwood T, Harris M, Patel A, Panaretto KS, Zwar N, Peiris D. What drives adoption of a computerised, multifaceted quality improvement intervention for cardiovascular disease management in primary healthcare settings? A mixed methods analysis using normalisation process theory. Implementation science : IS. 2018;13:140. doi:10.1186/s13012-018-0830-x.
Prebet C, Bouaud J, Guézennec G, Seroussi B. Taking into account the complementarity of contemporary breast cancer guidelines to leverage decision support in the DESIREE project. In: Studies in Health Technology and Informatics; 2018. p. 755–759. doi:10.3233/978-1-61499-852-5-755.
Rapoport MJ, Zucchero Sarracini C, Kiss A, Lee L, Byszewski A, Seitz DP, et al. Computer-Based Driving in Dementia Decision Tool With Mail Support: Cluster Randomized Controlled Trial. J Med Internet Res. 2018;20:e194. doi:10.2196/jmir.9126.
Reed H, et al. Impact of a Best Practice Alert Linking Clostridium difficile Infection Test Results to a Severity-Based Treatment Order Set. Open Forum Infectious Diseases. 2018;5:S79.
Ridgway JP, Almirol EA, Bender A, Richardson A, Schmitt J, Friedman E, et al. Which Patients in the Emergency Department Should Receive Preexposure Prophylaxis? Implementation of a Predictive Analytics Approach. AIDS Patient Care STDS. 2018;32:202–7. doi:10.1089/apc.2018.0011.
Rieckert A, Sommerauer C, Krumeich A, Sönnichsen A. Reduction of inappropriate medication in older populations by electronic decision support (the PRIMA-eDS study): a qualitative study of practical implementation in primary care. BMC family practice. 2018;19:110. doi:10.1186/s12875-018-0789-3.
Schoemans HM, Goris K, van Durm R, et al. Accuracy and usability of the eGVHD app in assessing the severity of graft-versus-host disease at the 2017 EBMT annual congress. Bone Marrow Transplant. 2018;53:490–4. doi:10.1038/s41409-017-0017-0.
Schoemans HM, Goris K, van Durm R, et al. The eGVHD app has the potential to improve the accuracy of graft-versus-host disease assessment: a multicenter randomized controlled trial. Haematologica. 2018;103:1698–707. doi:10.3324/haematol.2018.190777.
Sheibani R, et al. The Effect of a Clinical Decision Support System on Improving Adherence to Guideline in the Treatment of Atrial Fibrillation: An Interrupted Time Series Study. Journal of medical systems. 2018;42:26.

(continued)

List of primary studies (continued)

**Reference**
Singh K, Johnson L, Devarajan R, Shivashankar R, Sharma P, Kondal D, et al. Acceptability of a decision-support electronic health record system and its impact on diabetes care goals in South Asia: a mixed-methods evaluation of the CARRS trial. Diabetic medicine : a journal of the British Diabetic Association. 2018;35:1644–54. doi:10.1111/dme.13804.
Smith JC, Chen Q, Denny JC, Roden DM, Johnson KB, Miller RA. Evaluation of a novel system to enhance clinicians' recognition of preadmission adverse drug reactions. Appl Clin Inform. 2018;9:313–25. doi:10.1055/s-0038-1666697.
Somashekhar SP, Sepúlveda M-J, Puglielli S, Norden AD, Shortliffe EH, Kumar CR, et al. Watson for Oncology and breast cancer treatment recommendations: agreement with an expert multidisciplinary tumor board. Annals of oncology : official journal of the European Society for Medical Oncology. 2018;29:418–23. doi:10.1093/annonc/mdx781.
Sperl-Hillen JM, Crain AL, Margolis KL, Ekstrom HL, Appana D, Amundson G, et al. Clinical decision support directed to primary care patients and providers reduces cardiovascular risk: a randomized trial. Journal of the American Medical Informatics Association : JAMIA. 2018;25:1137–46. doi:10.1093/jamia/ocy085.
Suresh S, Saladino RA, Fromkin J, Heineman E, McGinn T, Richichi R, Berger RP. Integration of physical abuse clinical decision support into the electronic health record at a Tertiary Care Children's Hospital. J Am Med Inform Assoc. 2018;25:833–40. doi:10.1093/jamia/ocy025.
Thomas K, Kisely S, Urrego F. Electronic Heath Record Prompts May Increase Screening for Secondhand Smoke Exposure. Clinical pediatrics. 2018;57:27–30. doi:10.1177/0009922816688261.
van den Wijngaart LS, Geense WW, Boehmer AL, Brouwer ML, Hugen CAC, van Ewijk BE, et al. Barriers and Facilitators When Implementing Web-Based Disease Monitoring and Management as a Substitution for Regular Outpatient Care in Pediatric Asthma: Qualitative Survey Study. Journal of medical Internet research. 2018;20:e284-NA. doi:10.2196/jmir.9245.
Zimet G, Dixon BE, Xiao S, Tu W, Kulkarni A, Dugan T, et al. Simple and Elaborated Clinician Reminder Prompts for Human Papillomavirus Vaccination: A Randomized Clinical Trial. Academic Pediatrics. 2018;18:S66-S71. doi:10.1016/j.acap.2017.11.002.
Abimbola S, Patel B, Peiris D, Patel A, Harris M, Usherwood T, Greenhalgh T. The NASSS framework for ex post theorisation of technology-supported change in healthcare: worked example of the TORPEDO programme. BMC medicine. 2019;17:1–17. doi:10.1186/s12916-019-1463-x.
Al-Kalaldeh M, Suleiman K, Abu-Shahroor L, et al. The impact of introducing the Modified Early Warning Score 'MEWS' on emergency nurses' perceived role and self-efficacy: a quasi-experimental study. Int Emerg Nurs. 2019;45:25–30.
Bandong AN, Mackey M, Leaver A, et al. An interactive website for whiplash management (My Whiplash Navigator): process evaluation of design and implementation. JMIR Form Res. 2019;3:e12216. doi:10.2196/12216.
Bessat C, Zonon NA, D'Acremont V. Large-scale implementation of electronic Integrated Management of Childhood Illness (eIMCI) at the primary care level in Burkina Faso: a qualitative study on health worker perception of its medical content, usability and impact on antibiotic prescription and resistance. BMC Public Health. 2019;19:449. doi:10.1186/s12889-019-6692-6.
Bigham BL, Chan T, Skitch S, et al. Attitudes of emergency department physicians and nurses toward implementation of an early warning score to identify critically ill patients: qualitative explanations for failed implementation. CJEM. 2019;21:269–73.
Blecker S, others. Interrupting providers with clinical decision support to improve care for heart failure. International journal of medical informatics. 2019;131:103956.
Cai CJ, Reif E, Hegde N, Hipp J, Kim B, Smilkov D, et al. Human-centered tools for coping with imperfect algorithms during medical decision-making. In: ; 2019. p. 1–14. doi:10.1145/3293663.3293673.
Cai CJ, Winter S, Steiner D, Wilcox L. "Hello AI": uncovering the onboarding needs of medical practitioners for human-AI collaborative decision-making. Proc. ACM Hum. Comput. Interact. 2019;3:1–24. doi:10.1145/3359206.
Choi YI, Chung J-W, Kim KO, Kwon K an, Kim YJ, Park DK, et al. Concordance Rate between Clinicians and Watson for Oncology among Patients with Advanced Gastric Cancer: Early, Real-World Experience in Korea. Canadian journal of gastroenterology & hepatology. 2019;2019:8072928. doi:10.1155/2019/8072928.
Clebone A, Strupp KM, Whitney G, et al. Development and usability testing of the society for pediatric anesthesia pedi crisis mobile application. Anesth Analg. 2019;129:1635–44. doi:10.1213/ANE.0000000000003935.
Davis SE, Greevy RA, Fonnesbeck C, Lasko TA, Walsh CG, Matheny ME. A nonparametric updating method to correct clinical prediction model drift. J Am Med Inform Assoc. 2019;26:1448–57. doi:10.1093/jamia/ocz127.
Denton E, Hore-Lacy F, Radhakrishna N, Gilbert A, Tay TR, Lee J, et al. Severe Asthma Global Evaluation (SAGE): An Electronic Platform for Severe Asthma. The journal of allergy and clinical immunology. In practice. 2019;7:1440–9. doi:10.1016/j.jaip.2019.02.042.
Dodson CH, Baker EW, Bost K. Thematic analysis of nurse practitioners use of clinical decision support tools and clinical mobile apps for prescriptive purposes. Journal of the American Association of Nurse Practitioners. 2019;31:522–6. doi:10.1097/jxx.0000000000000170.
Ebker-White A, Bein KJ, Berendsen Russell S, Dinh MM. The Sydney triage to admission risk tool (START) to improve patient flow in an emergency department: a model of care implementation pilot study. BMC Emerg Med. 2019;19:79. doi:10.1186/s12873-019-0290-x.

(continued)

List of primary studies (continued)

**Reference**
Ekstrom HL, Kharbanda EO, Ballard DW, Vinson DR, Vazquez-Benitez G, Chettipally UK, et al. Development of a Clinical Decision Support System for Pediatric Abdominal Pain in Emergency Department Settings Across Two Health Systems Within the HCSRN. EGEMS (Wash DC). 2019;7:15. doi:10.5334/egems.282.
Fico G, Hernanzez L, Cancela J, Dagliati A, Sacchi L, Martinez-Millana A, et al. What do healthcare professionals need to turn risk models for type 2 diabetes into usable computerized clinical decision support systems? Lessons learned from the MOSAIC project. BMC medical informatics and decision making. 2019;19:1–16. doi:10.1186/s12911-019-0887-8.
Gill J, Kucharski K, Turk B, Pan C, Wei W. Using Electronic Clinical Decision Support in Patient-Centered Medical Homes to Improve Management of Diabetes in Primary Care: The DECIDE Study. The Journal of ambulatory care management. 2019;42:105–15. doi:10.1097/jac.0000000000000267.
Ginestra JC, Giannini HM, Schweickert WD, Meadows L, Lynch MJ, Pavan K, et al. Clinician perception of a machine learning-based early warning system designed to predict severe sepsis and septic shock. Crit Care Med. 2019;47:1477. doi:10.1097/CCM.0000000000003964.
Gold R, Bunce A, Cowburn S, Davis JV, Nelson JC, Nelson CC, et al. Does increased implementation support improve community clinics' guideline-concordant care? Results of a mixed methods, pragmatic comparative effectiveness trial. Implementation science : IS. 2019;14:100. doi:10.1186/s13012-019-0948-5.
Goodspeed A, Kostman N, Kriete TE, Longtine JW, Smith S, Marshall P, et al. Leveraging the utility of pharmacogenomics in psychiatry through clinical decision support: a focus group study. Annals of general psychiatry. 2019;18:13. doi:10.1186/s12991-019-0237-3.
Grau LE, Weiss J, O'Leary TK, Camenga D, Bernstein SL. Electronic decision support for treatment of hospitalized smokers: a qualitative analysis of physicians' knowledge, attitudes, and practices. Drug Alcohol Depend. 2019;194:296–301. doi:10.1016/j.drugalcdep.2018.10.006.
Green TA, Whitt S, Belden JL, Erdelez S, Shyu C-R. Medical calculators: prevalence, and barriers to use. Comput Methods Programs Biomed. 2019;179:105002. doi:10.1016/j.cmpb.2019.105002.
Gruendner J, Schwachhofer T, Sippl P, Wolf N, Erpenbeck M, Gulden C, et al. KETOS: clinical decision support and machine learning as a service - a training and deployment platform based on Docker, OMOP-CDM, and FHIR Web Services. PloS one. 2019;14:e0223010. doi:10.1371/journal.pone.0223010.
Guenter D, Abouzahra M, Schabort I, et al. Design process and utilization of a novel clinical decision support system for neuropathic pain in primary care: mixed methods observational study. JMIR Med Inform. 2019;7:e14141. doi:10.2196/14141.
Gupta S, Price C, Agarwal G, Chan D, Goel S, Boulet L-P, et al. The Electronic Asthma Management System (eAMS) improves primary care asthma management. The European respiratory journal. 2019;53:1802241-NA. doi:10.1183/13993003.02241-2018.
Gutenstein M, Pickering JW, Than M. Development of a digital clinical pathway for emergency medicine: lessons from usability testing and implementation failure. Health Informatics J. 2019;25:1563–71. doi:10.1177/1460458218779099.
Hendriks MP, Verbeek XA, van Vegchel T, van der Sangen MJ, Strobbe LJ, Merkus JW, et al. Transformation of the National Breast Cancer Guideline Into Data-Driven Clinical Decision Trees. JCO Clinical Cancer Informatics. 2019:1–14. doi:10.1200/CCI.18.00150.
Jensen C, McKerrow NH, Wills G. Acceptability and uptake of an electronic decision-making tool to support the implementation of IMCI in primary healthcare facilities in KwaZulu-Natal, South Africa. Paediatr Int Child Health. 2019;40:215–26. doi:10.1080/20469047.2019.1697573.
Johansson-Pajala R-M. Conditions for the Successful Implementation of Computer-Aided Drug Monitoring from Registered Nurses' Perspective : A Case Site Analysis. Computers, informatics, nursing : CIN. 2019;37:196–202. doi:10.1097/cin.0000000000000496.
Kercsmar CM, Sorkness CA, Calatroni A, Gergen PJ, Bloomberg GR, Gruchalla RS, et al. A computerized decision support tool to implement asthma guidelines for children and adolescents. The Journal of allergy and clinical immunology. 2019;143:1760–8. doi:10.1016/j.jaci.2018.10.060.
Kim EJ, Woo HS, Cho JH, Sym SJ, Baek J-H, Lee W-S, et al. Early experience with Watson for oncology in Korean patients with colorectal cancer. PloS one 2019. doi:10.1371/journal.pone.0213640.
Lopez PM, Divney A, Goldfeld K, Zanowiak J, Gore R, Kumar R, et al. Feasibility and Outcomes of an Electronic Health Record Intervention to Improve Hypertension Management in Immigrant-serving Primary Care Practices. Medical care. 2019;57:S164-S171. doi:10.1097/mlr.0000000000000994.
Majka DS, Lee JY, Peprah YA, Lipiszko D, Friesema EM, Ruderman E, Persell SD. Changes in Care After Implementing a Multifaceted Intervention to Improve Preventive Cardiology Practice in Rheumatoid Arthritis. American journal of medical quality : the official journal of the American College of Medical Quality. 2019;34:276–83. doi:10.1177/1062860618798719.
McCabe C, O'Brien M, Quirke MB. The introduction of the Early Warning Score in the Emergency Department: A retrospective cohort study. Int Emerg Nurs. 2019;45:31–5. doi:10.1016/j.ienj.2019.03.002.
McCarthy DM, Curtis LM, Courtney DM, Cameron KA, Lank PM, Kim HS, et al. A Multifaceted Intervention to Improve Patient Knowledge and Safe Use of Opioids: Results of the ED EMC2 Randomized Controlled Trial. Academic emergency medicine : official journal of the Society for Academic Emergency Medicine. 2019;26:1311–25. doi:10.1111/acem.13860.

(continued)

List of primary studies (continued)

**Reference**
McElroy T, Swartz EN, Hassani K, Waibel S, Tuff Y, Marshall C, et al. Implementation study of a 5-component pediatric early warning system (PEWS) in an emergency department in British Columbia, Canada, to inform provincial scale up. BMC Emerg Med. 2019;19:74. doi:10.1186/s12873-019-0287-5.
Melnick ER, Holland WC, Ahmed OM, Ma AK, Michael SS, Goldberg HS, et al. An integrated web application for decision support and automation of EHR workflow: a case study of current challenges to standards-based messaging and scalability from the EMBED trial. JAMIA open. 2019;2:434–9. doi:10.1093/jamiaopen/ooz053.
Miller MK, Mollen C, Behr K, Dowd MD, Miller E, Satterwhite CL, et al. Development of a novel computerized clinical decision support system to improve adolescent sexual health care provision. Acad Emerg Med. 2019;26:420–33. doi:10.1111/acem.13570.
Moschonis G, Michalopoulou M, Tsoutsoulopoulou K, Vlachopapadopoulou E, Michalacos S, Charmandari E, et al. Assessment of the effectiveness of a computerised decision-support tool for health professionals for the prevention and treatment of childhood obesity. Results from a randomised controlled trial. Nutrients. 2019;11:706. doi:10.3390/nu11030706.
Orchard J, Li J, Gallagher R, Freedman B, Lowres N, Neubeck L. Uptake of a primary care atrial fibrillation screening program (AF-SMART): a realist evaluation of implementation in metropolitan and rural general practice. BMC family practice. 2019;20:1–13. doi:10.1186/s12875-019-1058-9.
Pan J, Ding S, Wu D, Yang S, Yang J. Exploring behavioural intentions toward smart healthcare services among medical practitioners: a technology transfer perspective. International Journal of Production Research. 2019;57:5801–20. doi:10.1080/00207543.2018.1550272.
Pannebakker MM, Mills K, Johnson M, Emery JD, Walter FM. Understanding implementation and usefulness of electronic clinical decision support (eCDS) for melanoma in English primary care: a qualitative investigation. BJGP open. 2019;3:X101635. doi:10.3399/bjgpopen18X101635.
Reynolds TL, DeLucia PR, Esquibel KA, Gage T, Wheeler NJ, Randell JA, et al. Evaluating a handheld decision support device in pediatric intensive care settings. JAMIA open. 2019;2:49–61. doi:10.1093/jamiaopen/ooy055.
Richardson S, Feldstein D, McGinn T, et al. Live usability testing of two complex clinical decision support tools: observational study. JMIR Hum Factors. 2019;6:e12471. doi:10.2196/12471.
Rieckert A, Teichmann A-L, Drewelow E, Kriechmayr C, Piccoliori G, Woodham A, Sönnichsen A. Reduction of inappropriate medication in older populations by electronic decision support (the PRIMA-eDS project): a survey of general practitioners' experiences. J Am Med Inform Assoc. 2019;26:1323–32. doi:10.1093/jamia/ocz104.
Ritter C. User-Based Barriers to the Adoption of Artificial Intelligence in Healthcare: Capella University; 2019.
Segal G, Segev A, Brom A, Lifshitz Y, Wasserstrum Y, Zimlichman E. Reducing drug prescription errors and adverse drug events by application of a probabilistic, machine-learning based clinical decision support system in an inpatient setting. J Am Med Inform Assoc. 2019;26:1560–5. doi:10.1093/jamia/ocz129.
Silveira DV, Marcolino MS, Machado EL, et al. Development and evaluation of a mobile decision support system for hypertension management in the primary care setting in Brazil: mixed-methods field study on usability, feasibility, and utility. JMIR Mhealth Uhealth. 2019;7:e9869. doi:10.2196/mhealth.9869.
Trinkley KE, Blakeslee WW, Matlock DD, Kao DP, van Matre AG, Harrison R, et al. Clinician preferences for computerised clinical decision support for medications in primary care: a focus group study. BMJ health & care informatics. 2019;26:0. doi:10.1136/bmjhci-2019-000015.
Werk LN, Diaz MCG, Cadilla A, Franciosi JP, Hossain J. Promoting Adherence to Influenza Vaccination Recommendations in Pediatric Practice. Journal of primary care & community health. 2019;10:2150132719853061-NA. doi:10.1177/2150132719853061.
Wright A, McEvoy DS, Aaron S, McCoy AB, Amato MG, Kim H, et al. Structured override reasons for drug-drug interaction alerts in electronic health records. J Am Med Inform Assoc. 2019;26:934–44.
Yang Q, Steinfeld A, Zimmerman J. Unremarkable AI: fitting intelligent decision support into critical, clinical decision-making processes. In: ; 2019. p. 1–11. doi:10.1145/3293663.3293674.
Zhou N, Zhang C, Lv H, Hao C-X, Li T, Zhu J, et al. Concordance Study Between IBM Watson for Oncology and Clinical Practice for Patients with Cancer in China. The oncologist. 2019;24:812–9. doi:10.1634/theoncologist.2018-0255.
Bailey S, Hunt C, Brisley A, Howard S, Sykes L, Blakeman T. Implementation of clinical decision support to manage acute kidney injury in secondary care: an ethnographic study. BMJ Quality & Safety. 2020;29:382. doi:10.1136/bmjqs-2019-009932.
Baxter SL, Bass JS, Sitapati AM. Barriers to implementing an artificial intelligence model for unplanned readmissions. ACI Open. 2020;4:e108-e113. doi:10.1055/s-0040-1716748.
Baysari MT, Zheng WY, van Dort BA, Reid-Anderson H, Gronski M, Kenny E. A Late Attempt to Involve End Users in the Design of Medication-Related Alerts: Survey Study. Journal of medical Internet research. 2020;22:1–7. doi:10.2196/14855.
Beede E, Baylor E, Hersch F, Iurchenko A, Wilcox L, Ruamviboonsuk P, et al. A human-centered evaluation of a deep learning system deployed in clinics for the detection of diabetic retinopathy. In: ; 2020. p. 1–12. doi:10.1145/3313831.3376683.

(continued)

List of primary studies (continued)

**Reference**
Bersani K, Fuller TE, Garabedian P, Espares J, Mlaver E, Businger A, et al. Use, perceived usability, and barriers to implementation of a patient safety dashboard integrated within a vendor EHR. Appl Clin Inform. 2020;11:34–45. doi:10.1055/s-0039-3402756.
Blomaard LC, Mooijaart SP, Bolt S, Lucke JA, Gelder J de, Booijen AM, et al. Feasibility and acceptability of the 'Acutely Presenting Older Patient' screener in routine emergency department care. Age Ageing. 2020;49:1034–41. doi:10.1093/ageing/afaa078.
Burdick H, Pino E, Gabel-Comeau D, McCoy A, Gu C, Roberts J, et al. Effect of a sepsis prediction algorithm on patient mortality, length of stay and readmission: a prospective multicentre clinical outcomes evaluation of real-world patient data from US hospitals. BMJ Health Care Inform 2020. doi:10.1136/bmjhci-2019-100042.
Carlile M, Hurt B, Hsiao A, Hogarth M, Longhurst CA, Dameff C. Deployment of artificial intelligence for radiographic diagnosis of COVID-19 pneumonia in the emergency department. J Am Coll Emerg Physicians Open. 2020;1:1459–64.
Chen D, Wu L, Li Y, Zhang J, Liu J, Huang L, et al. Comparing blind spots of unsedated ultrafine, sedated, and unsedated conventional gastroscopy with and without artificial intelligence: a prospective, single-blind, 3-parallel-group, randomized, single-center trial. Gastrointest Endosc. 2020;91:332–9. doi:10.1016/j.gie.2019.09.020.
Chiang J, Kumar A, Morales D, Saini D, Hom J, Shieh L, et al. Physician usage and acceptance of a machine learning recommender system for simulated clinical order entry. AMIA Summits Translat. Sci. Proc. 2020;2020.
Corazza F, Snijders D, Arpone M, et al. Development and usability of a novel interactive tablet app (PediAppRREST) to support the management of pediatric cardiac arrest: Pilot high-fidelity simulation-based study. JMIR Mhealth Uhealth. 2020;8:e19070. doi:10.2196/19070.
Cox JL, others. Integrated Management Program Advancing Community Treatment of Atrial Fibrillation (IMPACT-AF): a cluster randomized trial of a computerized clinical decision support tool. American heart journal. 2020;224:35–46.
Fan W, Liu J, Zhu S, Pardalos PM. Investigating the impacting factors for the healthcare professionals to adopt artificial intelligence-based medical diagnosis support system (AIMDSS). Ann Oper Res. 2020;294:567–92. doi:10.1007/s10479-018-2818-y.
Gavelli F, Castello LM, Patrucco F, Bellan M, Sainaghi PP, Avanzi GC. Insights from Italy: the Novara-COVID Score for rapid destination of COVID-19 patients at Emergency Department presentation. Minerva Med. 2020;111:300–2. doi:10.23736/S0026-4806.20.06609-4.
Hendriks MP, Verbeek, Xander A. A. M., van Manen JG, van der Heijden SE, Go SHL, Gooiker GA, et al. Clinical decision trees support systematic evaluation of multidisciplinary team recommendations. Breast cancer research and treatment. 2020;183:355–63. doi:10.1007/s10549-020-05769-1.
Heselmans A, Delvaux N, Laenen A, et al. Computerized clinical decision support system for diabetes in primary care does not improve quality of care: a cluster-randomized controlled trial. Implement Sci. 2020;15:5. doi:10.1186/s13012-019-0955-6.
Jauk S, Kramer D, Großauer B, Rienmüller S, Avian A, Berghold A, et al. Risk prediction of delirium in hospitalized patients using machine learning: an implementation and prospective evaluation study. J Am Med Inform Assoc. 2020;27:1383–92. doi:10.1093/jamia/ocaa113.
Jin Z, Cui S, Guo S, Gotz D, Sun J. Carepre: an intelligent clinical decision assistance system. ACM Trans. Comput. Healthcare. 2020;1:1–20. doi:10.1145/3344258.
Jung SY, Hwang H, Lee K, Lee HY, Kim E, Kim M, et al. Barriers and facilitators to implementation of medication decision support systems in electronic medical records: mixed methods approach based on structural equation modeling and qualitative analysis. JMIR Med Inform. 2020;8:e18758.
Kim M-S, Park H-Y, Kho B-G, Park C-K, Oh I-J, Kim Y-C, et al. Artificial intelligence and lung cancer treatment decision: agreement with recommendation of multidisciplinary tumor board. Translational lung cancer research. 2020;9:507–14. doi:10.21037/tlcr.2020.04.11.
Klingberg A, Sawe HR, Hammar U, et al. M-health for burn injury consultations in a low-resource setting: an acceptability study among health care providers. Telemed J E Health. 2020;26:395–405. doi:10.1089/tmj.2019.0048.
Kumar A, Aikens RC, Hom J, Shieh L, Chiang J, Morales D, et al. OrderRex clinical user testing: a randomized trial of recommender system decision support on simulated cases. J. Am. Med. Inform. Assoc. 2020;27:1850–9. doi:10.1093/jamia/ocaa190.
Lam Shin Cheung J, Paolucci N, Price C, Sykes J, Gupta S. A system uptake analysis and GUIDES checklist evaluation of the Electronic Asthma Management System: A point-of-care computerized clinical decision support system. Journal of the American Medical Informatics Association : JAMIA. 2020;27:726–37. doi:10.1093/jamia/ocaa019.
Lemke AA, Thompson J, Hulick PJ, et al. Primary care physician experiences utilizing a family health history tool with electronic health record-integrated clinical decision support: an implementation process assessment. J Community Genet. 2020;11:339–50. doi:10.1007/s12687-020-00454-8.
Liu P-Y, Tsai Y-S, Chen P-L, Tsai H-P, Hsu L-W, Wang C-S, et al. Application of an artificial intelligence trilogy to accelerate processing of suspected patients with SARS-CoV-2 at a smart quarantine station: Observational study. J Med Internet Res. 2020;22:e19878. doi:10.2196/19878.
McKie PM, et al. Computerized Advisory decision support for cardiovascular diseases in primary care: a cluster randomized trial. The American Journal of Medicine. 2020;133:750–6.

(continued)

List of primary studies (continued)

Reference
Mulder-Wildemors LGM, Heringa M, Floor-Schreudering A, Jansen PAF, Bouvy ML. Reducing Inappropriate Drug Use in Older Patients by Use of Clinical Decision Support in Community Pharmacy: A Mixed-Methods Evaluation. Drugs & aging. 2020;37:115–23. doi:10.1007/s40266-019-00728-y.
Murphy ME, McSharry J, Byrne M, Boland F, Corrigan D, Gillespie P, et al. Supporting care for suboptimally controlled type 2 diabetes mellitus in general practice with a clinical decision support system: a mixed methods pilot cluster randomised trial. BMJ open. 2020;10:e032594-NA. doi:10.1136/bmjopen-2019-032594.
Pantanowitz L, Quiroga-Garza GM, Bien L, Heled R, Laifenfeld D, Linhart C, et al. An artificial intelligence algorithm for prostate cancer diagnosis in whole slide images of core needle biopsies: a blinded clinical validation and deployment study. Lancet Digit Health. 2020;2:e407-e416. doi:10.1016/S2589-7500(20)30159-X.
Paulsen MM, et al. Effects of using the MyFood decision support system on hospitalized patients' nutritional status and treatment: A randomized controlled trial. Clinical Nutrition. 2020;39:3607–17.
Petitgand C, Motulsky A, Denis J-L, Régis C. Investigating the barriers to physician adoption of an artificial intelligence-based decision support system in emergency care: an interpretative qualitative study. In: Digital personalized health and medicine: IOS Press; 2020. p. 1001–1005.
Pianykh OS, Langs G, Dewey M, Enzmann DR, Herold CJ, Schoenberg SO, Brink JA. Continuous learning AI in radiology: implementation principles and early applications. Radiology. 2020;297:6–14. doi:10.1148/radiol.2020200038.
Proffitt RD, Hooper G. Evaluation of the (qSOFA) Tool in the Emergency Department Setting: Nurse Perception and the Impact on Patient Care. Adv Emerg Nurs J. 2020;42:54–62. doi:10.1097/TME.0000000000000281.
Quan AML, Stiell I, Perry JJ, et al. Mobile clinical decision tools among emergency department clinicians: web-based survey and analytic data for evaluation of the Ottawa rules app. JMIR Mhealth Uhealth. 2020;8:e15503. doi:10.2196/15503.
Redjdal A, Bouaud J, Guézennec G, Gligorov J, Seroussi B. Creating synthetic patients to address interoperability issues: A case study with the management of breast cancer patients. In: Studies in Health Technology and Informatics; 2020. p. 177–181. doi:10.3233/SHTI200718.
Reynolds EL, et al. Randomized controlled trial of a clinical decision support system for painful polyneuropathy. Muscle & Nerve. 2020;61:640–4.
Rodríguez S, Sanz AM, Llano G, et al. Acceptability and usability of a mobile application for management and surveillance of vector-borne diseases in Colombia: an implementation study. PloS one. 2020;15:e0233269. doi:10.1371/journal.pone.0233269.
Romero-Brufau S, Wyatt KD, Boyum P, et al. A lesson in implementation: a pre-post study of providers' experience with artificial intelligence-based clinical decision support. Int J Med Inform. 2020;137:104072.
Romero-Brufau S, Wyatt KD, Boyum P, Mickelson M, Moore M, Cognetta-Rieke C. Implementation of artificial intelligence-based clinical decision support to reduce hospital readmissions at a regional hospital. Appl Clin Inform. 2020;11:570–7. doi:10.1055/s-0040-1711535.
Romero-Brufau S, Wyatt KD, Boyum P, Mickelson M, Moore M, Cognetta-Rieke C. A lesson in implementation: A pre-post study of providers' experience with artificial intelligence-based clinical decision support. International journal of medical informatics. 2020;137:104072-NA. doi:10.1016/j.ijmedinf.2019.104072.
Sandhu S, Lin AL, Brajer N, Sperling J, Ratliff W, Bedoya AD, et al. Integrating a Machine Learning System Into Clinical Workflows: Qualitative Study. J Med Internet Res. 2020;22:e22421. doi:10.2196/22421.
Sendak M, Elish MC, Gao M, Futoma J, Ratliff W, Nichols M, et al. "The human body is a black box" supporting clinical decision-making with deep learning. In: ; 2020. p. 99–109. doi:10.1145/3351095.3372850.
Sendak MP, Ratliff W, Sarro D, Alderton E, Futoma J, Gao M, et al. Real-World Integration of a Sepsis Deep Learning Technology Into Routine Clinical Care: Implementation Study. JMIR Med Inform. 2020;8:e15182. doi:10.2196/15182.
Shekarriz J, Keck T, Shekarriz H. Computerized Medical Evidence-Based Decision Assistance System "MEBDAS®" improves in-hospital outcome after pancreatoduodenectomy for pancreatic cancer. Pancreatology : official journal of the International Association of Pancreatology (IAP) ... [et al.]. 2020;20:746–50. doi:10.1016/j.pan.2020.04.007.
Sohn JH, Chillakuru YR, Lee S, Lee AY, Kelil T, Hess CP, et al. An open-source, vendor agnostic hardware and software pipeline for integration of artificial intelligence in radiology workflow. J Digit Imaging. 2020;33:1041–6. doi:10.1007/s10278-020-00348-8.
Strohm L, Hehakaya C, Ranschaert ER, Boon WP, Moors EH. Implementation of artificial intelligence (AI) applications in radiology: hindering and facilitating factors. Eur Radiol. 2020;30:5525–32. doi:10.1007/s00330-020-06946-y.
Subbaswamy A, Saria S. From development to deployment: dataset shift, causality, and shift-stable models in health AI. Biostatistics. 2020;21:345–52. doi:10.1093/biostatistics/kxz041.
Sutham K, Khuwuthyakorn P, Thinnukool O. Thailand medical mobile application for patients triage based on criteria based dispatch protocol. BMC Med Inform Decis Mak. 2020;20:66. doi:10.1186/s12911-020-1075-6.

(continued)

List of primary studies (continued)

Reference
Tian Y, Liu X, Wang Z, Cao S, Liu Z, Ji Q, et al. Concordance Between Watson for Oncology and a Multidisciplinary Clinical Decision-Making Team for Gastric Cancer and the Prognostic Implications: Retrospective Study. Journal of medical Internet research. 2020;22:e14122-NA. doi:10.2196/14122.
Westafer LM, Kunz A, Bugajska P, Hughes A, Mazor KM, Schoenfeld EM, et al. Provider perspectives on the use of evidence-based risk stratification tools in the evaluation of pulmonary embolism: a qualitative study. Acad Emerg Med. 2020;27:447–56. doi:10.1111/acem.13908.
Wijnberge M, Geerts BF, Hol L, Lemmers N, Mulder MP, Berge P, et al. Effect of a machine learning–derived early warning system for intraoperative hypotension vs standard care on depth and duration of intraoperative hypotension during elective noncardiac surgery: the HYPE randomized clinical trial. JAMA. 2020;323:1052–60. doi:10.1001/jama.2020.0660.
Zhao X, Zhang Y, Ma X, Chen Y, Xi J, Yin X, et al. Concordance between treatment recommendations provided by IBM Watson for Oncology and a multidisciplinary tumor board for breast cancer in China. Japanese journal of clinical oncology. 2020;50:852–8. doi:10.1093/jjco/hyaa051.
Zou F, Tang Y, Liu C, Ma J, Hu C. Concordance Study Between IBM Watson for Oncology and Real Clinical Practice for Cervical Cancer Patients in China: A Retrospective Analysis. Frontiers in genetics. 2020;11:200. doi:10.3389/fgene.2020.00200.
Abdulaal A, Patel A, Al-Hindawi A, Charani E, Alqahtani SA, Davies GW, et al. Clinical utility and functionality of an artificial intelligence–based app to predict mortality in COVID-19: mixed methods analysis. JMIR Format. Res. 2021;5:e27992. doi:10.2196/27992.
Aikemu B, Xue P, Hong H, Jia H, Wang C, Li S, et al. Artificial Intelligence in Decision-Making for Colorectal Cancer Treatment Strategy: An Observational Study of Implementing Watson for Oncology in a 250-Case Cohort. Frontiers in oncology. 2021;10:594182-NA. doi:10.3389/fonc.2020.594182.
Allen B, Agarwal S, Coombs L, Wald C, Dreyer K. 2020 ACR data science institute artificial intelligence survey. J Am Coll Radiol. 2021;18:1153–9.
Allen B, Dreyer K, Stibolt R, JR, Agarwal S, Coombs L, Treml C, et al. Evaluation and real-world performance monitoring of artificial intelligence models in clinical practice: try it, buy it, check it. J Am Coll Radiol. 2021;18:1489–96. doi:10.1016/j.jacr.2021.08.022.
Benrimoh D, Tanguay-Sela M, Perlman K, Israel S, Mehltretter J, Armstrong C, et al. Using a simulation centre to evaluate preliminary acceptability and impact of an artificial intelligence-powered clinical decision support system for depression treatment on the physician–patient interaction. BJPsych Open. 2021;7:e22. doi:10.1192/bjo.2020.127.
Blezek DJ, Olson-Williams L, Missert A, Korfiatis P. AI integration in the clinical workflow. J Digit Imaging. 2021;34:1435–46. doi:10.1007/s10278-021-00525-3.
Blomaard LC, Olthof M, Meuleman Y, Groot B de, Gussekloo J, Mooijaart SP. Experiences with and attitudes towards geriatric screening among older emergency department patients: a qualitative study. BMC Geriatr. 2021;21:198. doi:10.1186/s12877-021-02144-7.
Botwe BO, others. The integration of artificial intelligence in medical imaging practice: perspectives of African radiographers. Radiography. 2021;27:861–6. doi:10.1016/j.radi.2021.01.008.
Calisto FM, Santiago C, Nunes N, Nascimento JC. Introduction of human-centric AI to aid radiologists for multimodal breast image classification. International Journal of Human – Computer Studies. 2021;150:102607.
Coppola F, others. Artificial intelligence: radiologists' expectations and opinions gleaned from a nationwide online survey. Radiol Med. 2021;126:63–71. doi:10.1007/s11547-020-01205-y.
Dontchos BN, Yala A, Barzilay R, Xiang J, Lehman CD. External validation of a deep learning model for predicting mammographic breast density in routine clinical practice. Acad Radiol. 2021;28:475–80.
Eche T, Schwartz LH, Mokrane FZ, Dercle L. Toward generalizability in the deployment of artificial intelligence in radiology: role of computation stress testing to overcome underspecification. Radiol Artif Intell. 2021;3:e210097. doi:10.1148/ryai.2021210097.
Ellington LE, Najjingo I, Rosenfeld M, et al. Health workers' perspectives of a mobile health tool to improve diagnosis and management of paediatric acute respiratory illnesses in Uganda: a qualitative study. BMJ open. 2021;11:e049708. doi:10.1136/bmjopen-2021-049708.
Fenn A, Davis C, Buckland DM, et al. Development and validation of machine learning models to predict admission from emergency department to inpatient and intensive care units. Ann Emerg Med. 2021;78:290–302.
Garrett Fernandes M, Bussink J, Stam B, et al. Deep learning model for automatic contouring of cardiovascular substructures on radiotherapy planning CT images: dosimetric validation and reader study based clinical acceptability testing. Radiother Oncol. 2021;165:52–9.
Gavelli F, Castello LM, Bellan M, Azzolina D, Hayden E, Beltrame M, et al. Clinical stability and in-hospital mortality prediction in COVID-19 patients presenting to the Emergency Department. Minerva Med. 2021;112:118–23. doi:10.23736/S0026-4806.20.07074-3.
Gomes S, Wood D, Ayis S, Haliasos N, Roland D. Evaluation of a novel approach to recognising community-acquired paediatric sepsis at ED triage by combining an electronic screening algorithm with clinician assessment. Emerg Med J. 2021;38:132–8. doi:10.1136/emermed-2019-208746.
Hassan AE, Ringheanu VM, Preston L, Tekle W. Abstract P248: CSC Implementation of Artificial Intelligence Software Significantly Improves Door-In to Groin Puncture Time Interval and Recanalization Rates. Stroke. 2021;52:AP248-AP. doi:10.1161/STR.52.suppl_1.AP248.

(continued)

List of primary studies (continued)

**Reference**
Hogue S-C, Chen F, Brassard G, Lebel D, Bussières J-F, Durand A, et al. Pharmacists' perceptions of a machine learning model for the identification of atypical medication orders. J Am Med Inform Assoc. 2021;28:1712–8. doi:10.1093/jamia/ocab083.
Huisman M, others. An international survey on AI in radiology in 1041 radiologists and radiology residents part 2: expectations, hurdles to implementation, and education. Eur Radiol. 2021;31:8797–806. doi:10.1007/s00330-021-07782-4.
Jacobs M, He J, Pradier F, Lam M, Ahn B, McCoy AC, et al. Designing AI for trust and collaboration in time-constrained medical decisions: a sociotechnical lens. In: ; 2021. p. 1–14. doi:10.1145/3411764.3445413.
Jacobs M, Pradier MF, McCoy TH, Perlis RH, Doshi-Velez F, Gajos KZ. How machine-learning recommendations influence clinician treatment selections: the example of antidepressant selection. Translational Psychiatry. 2021;11:108. doi:10.1038/s41398-021-01224-x.
Jauk S, Kramer D, Avian A, Berghold A, Leodolter W, Schulz S. Technology Acceptance of a Machine Learning Algorithm Predicting Delirium in a Clinical Setting: a Mixed-Methods Study. J Med Syst. 2021;45:1–8. doi:10.1007/s10916-021-01759-2.
Jones CM, Danaher L, Milne MR, et al. Assessment of the effect of a comprehensive chest radiograph deep learning model on radiologist reports and patient outcomes: a real-world observational study. BMJ open. 2021;11:e052902.
Juluru K, Shih HH, Keshava Murthy KN, et al. Integrating AI algorithms into the clinical workflow. Radiol Artif Intell. 2021;3:e210013.
Keikes L, Kos M, Verbeek, Xander A. A. M., van Vegchel T, Nagtegaal ID, Lahaye MJ, et al. Conversion of a colorectal cancer guideline into clinical decision trees with assessment of validity. International journal for quality in health care : journal of the International Society for Quality in Health Care. 2021;33:NA-NA. doi:10.1093/intqhc/mzab051.
Kloub M, Gerigoorian A. A cross-sectional technology acceptance study of an AI CAD system in a breast screening unit 2021.
Laka M, Milazzo A, Merlin T. Factors that impact the adoption of Clinical Decision Support Systems (CDSS) for antibiotic management. Int J Environ Res Public Health. 2021;18:1901. doi:10.3390/ijerph18041901.
Lee MH, Siewiorek DP, Smailagic A, Bernardino A, Badia S. A human-ai collaborative approach for clinical decision making on rehabilitation assessment. In: ; 2021. p. 1–14. doi:10.1145/3411764.3445310.
Leiner T, Bennink E, Mol CP, Kuijf HJ, Veldhuis WB. Bringing AI to the clinic: blueprint for a vendor-neutral AI deployment infrastructure. Insights Imaging. 2021;12:11. doi:10.1186/s13244-020-00931-1.
Mammen JR, Java JJ, Halterman JS, Berliant MN, Crowley A, Frey SM, et al. Development and preliminary results of an Electronic Medical Record (EMR)-integrated smartphone telemedicine program to deliver asthma care remotely. Journal of telemedicine and telecare. 2021;27:217–30. doi:10.1177/1357633x19870025.
Marcolino MS, Oliveira JAQ, Cimini CCR, et al. Development and implementation of a decision support system to improve control of hypertension and diabetes in a resource-constrained area in Brazil: mixed methods study. J Med Internet Res. 2021;23:e18872. doi:10.2196/18872.
Minian N, Noormohamed A, Lingam M, et al. Integrating a brief alcohol intervention with tobacco addiction treatment in primary care: qualitative study of health care practitioner perceptions. Addict Sci Clin Pract. 2021;16:17. doi:10.1186/s13722-021-00225-x.
Morey JR, Zhang X, Yaeger KA, Fiano E, Marayati NF, Kellner CP, et al. Real-World Experience with Artificial Intelligence-Based Triage in Transferred Large Vessel Occlusion Stroke Patients. Cerebrovasc Dis. 2021;50:450–5. doi:10.1159/000514263.
Morrison K. Artificial intelligence and the NHS: a qualitative exploration of the factors influencing adoption. Future Healthc J. 2021;8:e648. doi:10.7861/fhj.2020-0258.
Mugabe KV. Barriers and facilitators to the adoption of artificial intelligence in radiation oncology: a New Zealand study. Tech Innov Patient Support Radiat Oncol. 2021;18:16–21. doi:10.1016/j.tipsro.2021.03.004.
Müller A, Mertens SM, Göstemeyer G, Krois J, Schwendicke F. Barriers and enablers for artificial intelligence in dental diagnostics: a qualitative study. Journal of Clinical Medicine. 2021;10:1612.
Pierce JD, Rosipko B, Youngblood L, Gilkeson RC, Gupta A, Bittencourt LK. Seamless integration of artificial intelligence into the clinical environment: our experience with a novel pneumothorax detection artificial intelligence algorithm. J Am Coll Radiol. 2021;18:1497–505. doi:10.1016/j.jacr.2021.08.023.
Popescu C, Golden G, Benrimoh D, Tanguay-Sela M, Slowey D, Lundrigan E, et al. Evaluating the Clinical Feasibility of an Artificial Intelligence-Powered, Web-Based Clinical Decision Support System for the Treatment of Depression in Adults: Longitudinal Feasibility Study. JMIR Form Res. 2021;5:e31862. doi:10.2196/31862.
Prakash AV, Das S. Medical practitioner's adoption of intelligent clinical diagnostic decision support systems: a mixed-methods study. Inf Manage 2021. doi:10.1016/j.im.2021.103524.
Pumplun L, Fecho M, Wahl-Islam N, Buxmann P. Machine learning systems in clinics – how mature is the adoption process in medical diagnostics? In: ; 2021.
Qurashi AA, Alanazi RK, Alhazmi YM, Almohammadi AS, Alsharif WM, Alshamrani KM. Saudi radiology personnel's perceptions of artificial intelligence implementation: a cross-sectional study. J Multidiscip Healthc. 2021;14:3225–31. doi:10.2147/jmdh.S340786.

(continued)

List of primary studies (continued)

**Reference**
Redjdal A, Bouaud J, Guézennec G, Gligorov J, Seroussi B. Reusing decisions made with one decision support system to assess a second decision support system: Introducing the notion of complex cases. In: Public Health and Informatics: Proceedings of MIE 2021; 2021. p. 649–653. doi:10.3233/SHTI210251.
Repici A, Spadaccini M, Antonelli G, Correale L, Maselli R, Galtieri PA, et al. Artificial intelligence and colonoscopy experience: lessons from two randomised trials. Gut. 2021;71:757–65. doi:10.1136/gutjnl-2020-322847.
Salwei ME, Carayon P, Hoonakker PL, Hundt AS, Wiegmann D, Pulia M, et al. Workflow integration analysis of a human factors-based clinical decision support in the emergency department. Appl Ergon. 2021;97:103498. doi:10.1016/j.apergo.2021.103498.
Scheetz J, Koca D, McGuinness M, et al. Real-world artificial intelligence-based opportunistic screening for diabetic retinopathy in endocrinology and indigenous healthcare settings in Australia. Sci Rep. 2021;11:15808.
Tran AQ, Nguyen LH, Nguyen HSA, Nguyen CT, Vu LG, Zhang M, et al. Determinants of Intention to Use Artificial Intelligence-Based Diagnosis Support System Among Prospective Physicians. Frontiers in Public Health. 2021;9.
Trinkley KE, Kroehl ME, Kahn MG, et al. Applying clinical decision support design best practices with the practical robust implementation and sustainability model versus reliance on commercially available clinical decision support tools: randomized controlled trial. JMIR Med Inform. 2021;9:e24359. doi:10.2196/24359.
van der Stap L, Heij AH de, van der Heide A, Reyners AK, van der Linden YM. Clinical decision support system to optimise symptom management in palliative medicine: focus group study. BMJ Support Palliat Care 2021. doi:10.1136/bmjspcare-2021-002940.
Verma AA, Murray J, Greiner R, Cohen JP, Shojania KG, Ghassemi M, et al. Implementing machine learning in medicine. CMAJ. 2021;193:E1351-E1357. doi:10.1503/cmaj.202434.
Wang B, Jin S, Yan Q, Xu H, Luo C, Wei L, et al. AI-assisted CT imaging analysis for COVID-19 screening: building and deploying a medical AI system. Appl Soft Comput. 2021;98:106897. doi:10.1016/j.asoc.2020.106897.
Wang D, Wang L, Zhang Z, Zhu H, Gao Y, et al. "Brilliant AI doctor" in rural clinics: challenges in AI-powered clinical decision support system deployment. In: ; 2021. p. 1–18. doi:10.1145/3411764.3445525.
Wiggins WF, Magudia K, Schmidt TM, O'Connor SD, Carr CD, Kohli MD, Andriole KP. Imaging AI in practice: a demonstration of future workflow using integration standards. Radiol Artif Intell. 2021;3:e210152. doi:10.1148/ryai.2021210152.
Wilson A, Saeed H, Pringle C, Eleftheriou I, Bromiley PA, Brass A. Artificial intelligence projects in healthcare: 10 practical tips for success in a clinical environment. BMJ Health Care Inform. 2021;28:e100323. doi:10.1136/bmjhci-2021-100323.
Wong J, Huang V, Wells D, et al. Implementation of deep learning-based auto-segmentation for radiotherapy planning structures: a workflow study at two cancer centers. Radiat Oncol. 2021;16:101.
Yao X, Rushlow DR, Inselman JW, McCoy RG, Thacher TD, Behnken EM, et al. Artificial intelligence–enabled electrocardiograms for identification of patients with low ejection fraction: a pragmatic, randomized clinical trial. Nat Med. 2021;27:815–9. doi:10.1038/s41591-021-00423-1.
Zhai H, others. Radiation oncologists' perceptions of adopting an artificial intelligence–assisted contouring technology: model development and questionnaire study. J Med Internet Res 2021. doi:10.2196/27122.
Zhang X, Svec M, Tracy R, Ozanich G. Clinical decision support systems with team-based care on type 2 diabetes improvement for Medicaid patients: a quality improvement project. Int J Med Inform. 2021;158:104626.
Abuzaid MM, Elshami W, Tekin H, Issa B. Assessment of the willingness of radiologists and radiographers to accept the integration of artificial intelligence into radiology practice. Acad Radiol. 2022;29:87–94. doi:10.1016/j.acra.2020.09.014.
Alamoudi A. Acceptance of artificial intelligence (AI) and machine learning (ML) among radiologists in Saudi Arabia. Int J Adv Appl Sci. 2022;9:154–7. doi:10.21833/ijaas.2022.01.018.
Alcorn SR, LaVigne AW, Elledge CR, Fiksel J, Hu C, Kleinberg L, et al. Evaluation of the Clinical Utility of the Bone Metastases Ensemble Trees for Survival Decision Support Platform (BMETS-DSP): A Case-Based Pilot Assessment. JCO Clinical Cancer Informatics. 2022:e2200082. doi:10.1200/CCI.22.00082.
Aldhafeeri FM. Perspectives of radiographers on the emergence of artificial intelligence in diagnostic imaging in Saudi Arabia. Insights Imaging. 2022;13:178. doi:10.1186/s13244-022-01319-z.
Aldughayfiq B, Sampalli S. Patients', pharmacists', and prescribers' attitude toward using blockchain and machine learning in a proposed ePrescription system: online survey. JAMIA open. 2022;5:ooab115.
Ankolekar A, van der Heijden B, Dekker A, et al. Clinician perspectives on clinical decision support systems in lung cancer: Implications for shared decision-making. Health Expect. 2022;25:1342–51.
Calisto FM, Santiago C, Nunes N, Nascimento JC. BreastScreening-AI: evaluating medical intelligent agents for human-AI interactions. Artif Intell Med 2022. doi:10.1016/j.artmed.2022.102285.

(continued)

List of primary studies (continued)

**Reference**
Calisto FM, Nunes N, Nascimento JC. Modeling adoption of intelligent agents in medical imaging. International Journal of Human-Computer Studies. 2022;168:102922. doi:10.1016/j.ijhcs.2022.102922.
Cheikh AB, Gorincour G, Nivet H, et al. How artificial intelligence improves radiological interpretation in suspected pulmonary embolism. Eur Radiol. 2022;32:5831–42.
Cheng M, Li X, Xu J. Promoting Healthcare Workers' Adoption Intention of Artificial-Intelligence-Assisted Diagnosis and Treatment: The Chain Mediation of Social Influence and Human–Computer Trust. International Journal of Environmental Research and Public Health 2022. doi:10.3390/ijerph192013311.
Choudhury A, Asan O, Medow JE. Effect of risk, expectancy, and trust on clinicians' intent to use an artificial intelligence system – Blood Utilization Calculator. Appl Ergon. 2022;101:103708.
Cornelissen L, Egher C, van Beek V, Williamson L, Hommes D. The Drivers of Acceptance of Artificial Intelligence–Powered Care Pathways Among Medical Professionals: Web-Based Survey Study. JMIR Form Res. 2022;6:e33368. doi:10.2196/33368.
Creed TA, Kuo PB, Oziel R, et al. Knowledge and attitudes toward an artificial intelligence-based fidelity measurement in community cognitive behavioral therapy supervision. Adm Policy Ment Health. 2022;49:343–56.
Ebben, Kees C W J, Hendriks MP, Markus L, Kos M, Hingh IHJT de, Oddens JR, et al. Using guideline-based clinical decision support in oncological multidisciplinary team meetings: A prospective, multicenter concordance study. International journal for quality in health care : journal of the International Society for Quality in Health Care. 2022;34:NA-NA. doi:10.1093/intqhc/mzac007.
Eschert T, Schwendicke F, Krois J, Bohner L, Vinayahalingam S, Hanisch M. A survey on the use of artificial intelligence by clinicians in dentistry and oral and maxillofacial surgery. Medicina-Lithuania. 2022;58:1059. doi:10.3390/medicina58081059.
Fujimori R, Liu K, Soeno S, Naraba H, Ogura K, Hara K, et al. Acceptance, barriers, and facilitators to implementing artificial intelligence-based decision support systems in emergency departments: quantitative and qualitative evaluation. JMIR Form Res. 2022;6:e36501. doi:10.2196/36501.
Goel K, Sindhgatta R, Kalra S, Goel R, Mutreja P. The effect of machine learning explanations on user trust for automated diagnosis of COVID-19. Comput Biol Med. 2022;146:105587.
Hendrix N, others. Radiologist preferences for artificial intelligence-based decision support during screening mammography interpretation. J Am Coll Radiol. 2022;19:1098–110. doi:10.1016/j.jacr.2022.06.019.
Jaber D, Hajj H, Maalouf F, El-Hajj W. Medically-oriented design for explainable AI for stress prediction from physiological measurements. BMC Med Inform Decis Mak. 2022;22:38.
Joshi M, Mecklai K, Rozenblum R, Samal L. Implementation approaches and barriers for rule-based and machine learning-based sepsis risk prediction tools: a qualitative study. JAMIA open. 2022;5:ooac022. doi:10.1093/jamiaopen/ooac022.
Kanakaraj P, Ramadass K, Bao S, Basford M, Jones LM, Lee HH, et al. Workflow integration of research AI tools into a hospital radiology rapid prototyping environment. J Digit Imaging. 2022;35:1023–33. doi:10.1007/s10278-022-00601-2.
Kim EY, Kim YJ, Choi WJ, et al. Concordance rate of radiologists and a commercialised deep-learning solution for chest X-ray: real-world experience with a multicenter health screening cohort. PloS one. 2022;17:e0264383.
Künzel LA, Nachbar M, Hagmüller M, et al. Clinical evaluation of autonomous, unsupervised planning integrated in MR-guided radiotherapy for prostate cancer. Radiother Oncol. 2022;168:229–33.
Lupei MI, Li D, Ingraham NE, Baum KD, Benson B, Puskarich M, et al. A 12-hospital prospective evaluation of a clinical decision support prognostic algorithm based on logistic regression as a form of machine learning to facilitate decision making for patients with suspected COVID-19. PloS one. 2022;17.
Lutz W, Deisenhofer A-K, Rubel J, Bennemann B, Giesemann J, Poster K, Schwartz B. Prospective evaluation of a clinical decision support system in psychological therapy. J Consult Clin Psychol. 2022;90:90–106. doi:10.1037/ccp0000642.
Macchia G, Ferrandina G, Patarnello S, Autorino R, Masciocchi C, Pisapia V, et al. Multidisciplinary Tumor Board Smart Virtual Assistant in Locally Advanced Cervical Cancer: A Proof of Concept. Frontiers in oncology. 2022;11:797454-NA. doi:10.3389/fonc.2021.797454.
Mammen JR, Schoonmaker JD, Java JJ, Halterman JS, Berliant MN, Crowley A, et al. Going mobile with primary care: smartphone-telemedicine for asthma management in young urban adults (TEAMS). The Journal of asthma : official journal of the Association for the Care of Asthma. 2022;59:1–13. doi:10.1080/02770903.2020.1830413.
Moret-Tatay C, Radawski HM, Guariglia C. Health professionals' experience using an azure voice-bot to examine cognitive impairment (WAY2AGE). Healthcare (Basel). 2022;10:783.
Pangti R, Gupta S, Gupta P, Dixit A, Sati HC. Acceptability of artificial intelligence among Indian dermatologists. Indian Journal of Dermatology, Venereology and Leprology 2022. doi:10.25259/IJDVL_210_2021.
Panigutti C, Beretta A, Giannotti F, Pedreschi D. Understanding the impact of explanations on advice-taking: a user study for AI-based clinical Decision Support Systems. In: New York, NY, USA: Association for Computing Machinery; 2022. doi:10.1145/3491102.3502104.

(continued)

List of primary studies (continued)

**Reference**
Ploegmakers KJ, Medlock S, Linn AJ, Lin Y, Seppälä LJ, Petrovic M, et al. Barriers and facilitators in using a Clinical Decision Support System for fall risk management for older people: a European survey. Eur Geriatr Med. 2022;13:395–405. doi:10.1007/s41999-021-00599-w.
Pou-Prom C, Murray J, Kuzulugil S, Mamdani M, Verma AA. From compute to care: lessons learned from deploying an early warning system into clinical practice. Front Digit Health. 2022;4:932123. doi:10.3389/fdgth.2022.932123.
Rabinovich D, Mosquera C, Torrens P, Aineseder M, Benitez S. User satisfaction with an AI system for chest X-ray analysis implemented in a hospital's emergency setting. Stud Health Technol Inform. 2022;294:8–12. doi:10.3233/SHTI220386.
Rainey C, others. UK reporting radiographers' perceptions of AI in radiographic image interpretation - current perspectives and future developments. Radiography. 2022;28:881–8. doi:10.1016/j.radi.2022.06.006.
Scheder-Bieschin J, Blümke B, Buijzer E de, et al. Improving emergency department patient-physician conversation through an artificial intelligence symptom-taking tool: mixed methods pilot observational study. JMIR Form Res. 2022;6:e28199.
Shelmerdine SC, Rosendahl K, Arthurs OJ. Artificial intelligence in paediatric radiology: international survey of health care professionals' opinions. Pediatric Radiology. 2022;52:30–41. doi:10.1007/s00247-021-05195-5.
Shiang T, Garwood E, Debenedectis CM. Artificial intelligence-based decision support system (AI-DSS) implementation in radiology residency: introducing residents to AI in the clinical setting. Clinical Imaging. 2022;92:32–7. doi:10.1016/j.clinimag.2022.09.003.
Soltan AA, Yang J, Pattanshetty R, Novak A, Yang Y, Rohanian O, et al. Real-world evaluation of rapid and laboratory-free COVID-19 triage for emergency care: external validation and pilot deployment of artificial intelligence driven screening. Lancet Digit Health. 2022;4:e266-e278. doi:10.1016/S2589-7500(21)00272-7.
Svedberg P, Reed J, Nilsen P, Barlow J, Macrae C, Nygren J. Toward successful implementation of artificial intelligence in health care practice: protocol for a research program. JMIR Res Protoc. 2022;11:e34920. doi:10.2196/34920.
Tamori H, Yamashina H, Mukai M, Morii Y, Suzuki T, Ogasawara K. Acceptance of the Use of Artificial Intelligence in Medicine Among Japan's Doctors and the Public: A Questionnaire Survey. JMIR Hum Factors. 2022;9:e24680. doi:10.2196/24680.
Tanguay-Sela M, Benrimoh D, Popescu C, Perez T, Rollins C, Snook E, et al. Evaluating the perceived utility of an artificial intelligence-powered clinical decision support system for depression treatment using a simulation center. Psychiatry Res. 2022;308:114336. doi:10.1016/j.psychres.2021.114336.
Agrawal A, Khatri GD, Khurana B, Sodickson AD, Liang Y, Dreizin D. A survey of ASER members on artificial intelligence in emergency radiology: trends, perceptions, and expectations. Emerg Radiol. 2023;30:267–77. doi:10.1007/s10140-023-02121-0.
Baysari MT, van Dort BA, Stanceski K, Hargreaves A, Zheng WY, Moran M, et al. Is evidence of effectiveness a driver for clinical decision support selection? A qualitative descriptive study of senior hospital staff. Int J Qual Health Care. 2023;35:mzad004. doi:10.1093/intqhc/mzad004.
Catalina QM, others. Knowledge and perception of primary care healthcare professionals on the use of artificial intelligence as a healthcare tool. Digit Health. 2023;9:20552076231180511. doi:10.1177/20552076231180511.
Dalvi-Esfahani M, Mosharaf-Dehkordi M, Leong LW, Ramayah T, Jamal Kanaan-Jebna AM. Exploring the drivers of XAI-enhanced clinical decision support systems adoption: Insights from a stimulus-organism-response perspective. Technological Forecasting and Social Change. 2023;195:122768. doi:10.1016/j.techfore.2023.122768.
Edzie EKM, others. Perspectives of radiologists in Ghana about the emerging role of artificial intelligence in radiology. Heliyon 2023. doi:10.1016/j.heliyon.2023.e15558.
Eiskjær S, Pedersen CF, Skov ST, Andersen MØ. Usability and performance expectancy govern spine surgeons' use of a clinical decision support system for shared decision-making on the choice of treatment of common lumbar degenerative disorders. Frontiers in Digital Health. 2023;5.
Griewing S, Gremke N, Wagner U, Lingenfelder M, Kuhn S, Boekhoff J. Challenging ChatGPT 3.5 in Senology-An Assessment of Concordance with Breast Cancer Tumor Board Decision Making. Journal of personalized medicine. 2023;13:1502. doi:10.3390/jpm13101502.
Hamd ZY, Elshami W, Al Kawas S, Aljuaid H, Abuzaid MM. A closer look at the current knowledge and prospects of artificial intelligence integration in dentistry practice: a cross-sectional study. Heliyon 2023. doi:10.1016/j.heliyon.2023.e17089.
Haugsten ER, Vestergaard T, Trettin B. Experiences regarding use and implementation of artificial intelligence-supported follow-up of atypical moles at a dermatological outpatient clinic: qualitative study. JMIR Dermatol. 2023;6:1. doi:10.2196/44913.
Hsieh P-J. Determinants of physicians' intention to use AI-assisted diagnosis: An integrated readiness perspective. Computers in Human Behavior. 2023;147:107868. doi:10.1016/j.chb.2023.107868.
King H, Williams B, Treanor D, Randell R. How, for whom, and in what contexts will artificial intelligence be adopted in pathology? A realist interview study. J Am Med Inform Assoc. 2023;30:529–38. doi:10.1093/jamia/ocac254.
Kleine A-K, Kokje E, Lermer E, Gaube S. Attitudes Toward the Adoption of 2 Artificial Intelligence–Enabled Mental Health Tools Among Prospective Psychotherapists: Cross-sectional Study. JMIR Hum Factors. 2023;10:e46859. doi:10.2196/46859.

(continued)

List of primary studies (continued)

Reference
Lukac S, Dayan D, Fink V, Leinert E, Hartkopf A, Veselinovic K, et al. Evaluating ChatGPT as an adjunct for the multidisciplinary tumor board decision-making in primary breast cancer cases. Archives of gynecology and obstetrics. 2023;308:1831–44. doi:10.1007/s00404-023-07130-5.
Ng SST, Oehring R, Ramasetti N, Roller R, Thomas P, Chen Y, et al. Concordance of a decision algorithm and multidisciplinary team meetings for patients with liver cancer-a study protocol for a randomized controlled trial. Trials. 2023;24:577-NA. doi:10.1186/s13063-023-07610-8.
O'Connor H, Melanophy G, Martin CM, Flattery M, O'Dea E. Transition to ePrescribing for systemic anti-cancer therapy - Perceptions of a multidisciplinary haematology/oncology team in a large teaching hospital. J Oncol Pharm Pract. 2023;29:1404–17. doi:10.1177/10781552221126102.
Thavanesan N, Bodala I, Walters Z, Ramchurn S, Underwood TJ, Vigneswaran G. Machine learning to predict curative multidisciplinary team treatment decisions in oesophageal cancer. European journal of surgical oncology : the journal of the European Society of Surgical Oncology and the British Association of Surgical Oncology. 2023;49:106986. doi:10.1016/j.ejso.2023.106986.
Verma H, others. Rethinking the role of AI with physicians in oncology: revealing perspectives from clinical and research workflows. In: Hamburg, Germany; 2023. doi:10.1145/3544548.3581506.
Wang W, Chen L, Xiong M, Wang Y. Accelerating AI Adoption with Responsible AI Signals and Employee Engagement Mechanisms in Health Care. Information Systems Frontiers. 2023;25:2239–56. doi:10.1007/s10796-021-10154-4.
Yang X, Man D, Yun K, zhang S, Han X. Factors influencing doctors' acceptance of artificial intelligence-enabled clinical decision support systems in tertiary hospitals in China; 2023.
