# Supplementary material for: Barriers and facilitators to the implementation and adoption of computerised clinical decision support systems: an overview of reviews
Source: Syst Rev. 2026 May 13;15:166. doi: 10.1186/s13643-026-03200-2 (PMC13173960; doi:10.1186/s13643-026-03200-2)
Supplement: Supplementary file 5 — Additional file 5. Influencing factors: Mapping to the included reviews. [file 13643_2026_3200_MOESM5_ESM.pdf]

Influencing factors: Mapping to included reviews

**Influencing factor**	Abell et al. (2023)	Adepoju et al. (2017)	Araujo et al. (2020)	Chan et al. (2023)	Chen et al. (2022)	Chima et al. (2019)	Devaraj et al. (2014)	Dingel et al. (2024)	Fernández-Barceló et al. (2023)	Gao et al. (2022)	Hendriks et al. (2024)	Higgins et al. (2023)	Hua et al. (2024)	Jun et al. (2018)	Kamel Rahimi et al. (2024)	Kilsdonk et al. (2017)	Kouri et al. (2022)	Liu et al. (2021)	Marcilly et al. (2015)	Meunier et al. (2023)	Miller et al. (2015)	Moxey et al. (2010)	Olouch et al. (2012)	Perivolaris et al. (2024)	Shakibaei Bonakdeh et al. (2024)	Tricco et al. (2023)	van Dort et al. (2019)	Wang et al. (2023)	Westerbeek et al. (2021)	Wohlgemut et al. (2023)
Usefulness and perceived benefits	x	x		x	x		x	x		x	x		x	x	x	x		x	x	x		x		x	x	x	x		x	x
Trust	x	x	x	x	x	x	x	x				x	x		x	x				x				x	x	x	x	x	x	x
Attitude/Preferences/Values	x			x			x	x		x	x		x			x				x		x		x			x	x	x	x
Perceived autonomy of action	x				x				x		x		x			x		x		x	x	x		x			x	x	x	
Resistance/Readiness to use/technology/change			x		x			x		x			x			x				x		x		x					x	x
Agreement with recommendations					x	x	x			x	x					x				x			x	x					x	
Beliefs about impact on professional competence					x						x			x		x				x		x				x	x		x	
Need for support/CDSS					x											x				x		x		x			x	x	x	
Stress										x						x				x		x		x						
Role and responsibility	x																			x		x		x						
Prior experiences							x																					x	x	
Frustration			x																										x	x
Confidence in using the system					x								x							x				x						
Satisfaction					x																			x						
Voluntariness										x						x														
Job security																				x				x						
Perceived risk								x																x						
Medicolegal concerns	x			x										x						x		x		x			x	x		x
Security/privacy concerns	x									x	x				x					x		x	x	x						
Knowledge of the system	x			x			x			x						x				x			x	x	x				x	

(continued)

Influencing factors: Mapping to included reviews (continued)

**Influencing factor**	Abell et al. (2023)	Adepoju et al. (2017)	Araujo et al. (2020)	Chan et al. (2023)	Chen et al. (2022)	Chima et al. (2019)	Devaraj et al. (2014)	Dingel et al. (2024)	Fernández-Barceló et al. (2023)	Gao et al. (2022)	Hendriks et al. (2024)	Higgins et al. (2023)	Hua et al. (2024)	Jun et al. (2018)	Kamel Rahimi et al. (2024)	Kilsdonk et al. (2017)	Kouri et al. (2022)	Liu et al. (2021)	Marcilly et al. (2015)	Meunier et al. (2023)	Miller et al. (2015)	Moxey et al. (2010)	Olouch et al. (2012)	Perivolaris et al. (2024)	Shakibaei Bonakdeh et al. (2024)	Tricco et al. (2023)	van Dort et al. (2019)	Wang et al. (2023)	Westerbeek et al. (2021)	Wohlgemut et al. (2023)
Awareness of the system	x		x	x	x		x							x		x				x							x		x	
Clarity of the purpose of the system	x												x	x		x				x				x		x				x
Training	x	x	x	x	x		x		x	x	x			x	x	x				x		x	x	x		x	x	x	x	x
Technical skills	x		x				x			x	x		x		x	x				x		x	x					x	x	
Education and improvement	x			x	x		x			x						x				x		x		x			x		x	x
Professional experience or expertise				x						x						x				x							x		x	x
Familiarity with CDSS					x					x					x	x								x						x
Social acceptance (use/opinion by others)							x	x					x	x		x													x	x
Discrete accessibility							x																						x	
Conflicts due to CDSS use																				x										
Age																x				x										
Usability / ease of use	x			x	x		x	x	x	x	x			x	x	x		x	x	x		x		x	x	x	x	x	x	x
Design (UX)	x			x	x		x			x	x					x			x	x		x		x		x	x		x	x
Interoperability, Integration and information standards	x				x		x		x	x	x				x	x				x	x	x			x			x	x	
(Local) feasibility/applicability				x	x		x			x	x					x				x		x	x	x	x				x	x
Flexibility/adaptability			x				x			x	x				x	x			x	x					x	x			x	x
Decision model complexity / Transferability	x			x	x					x	x				x	x				x	x							x	x	
Transparency/Explainability											x	x				x			x	x				x		x		x		

(continued)

Influencing factors: Mapping to included reviews (continued)

**Influencing factor**	Abell et al. (2023)	Adepoju et al. (2017)	Araujo et al. (2020)	Chan et al. (2023)	Chen et al. (2022)	Chima et al. (2019)	Devaraj et al. (2014)	Dingel et al. (2024)	Fernández-Barceló et al. (2023)	Gao et al. (2022)	Hendriks et al. (2024)	Higgins et al. (2023)	Hua et al. (2024)	Jun et al. (2018)	Kamel Rahimi et al. (2024)	Kilsdonk et al. (2017)	Kouri et al. (2022)	Liu et al. (2021)	Marcilly et al. (2015)	Meunier et al. (2023)	Miller et al. (2015)	Moxey et al. (2010)	Olouch et al. (2012)	Perivolaris et al. (2024)	Shakibaei Bonakdeh et al. (2024)	Tricco et al. (2023)	van Dort et al. (2019)	Wang et al. (2023)	Westerbeek et al. (2021)	Wohlgemut et al. (2023)
Correct functionality (Technical reliability)					x											x			x	x				x		x			x	
Product standardisation				x						x															x				x	
Device (size and shape)																x				x										x
Active vs. passive CDSS																				x									x	
Novelty (of technology)																													x	x
Performance assessment															x									x						
Decision model validity / reliability of evidence base				x	x		x			x	x			x		x				x	x		x	x	x		x		x	
Correctness/accuracy (output)				x						x				x		x			x	x		x	x	x		x		x	x	x
Relevance (output)	x				x					x				x		x			x	x		x		x			x	x	x	
Alert fatigue	x						x		x	x					x	x			x	x		x				x	x		x	
Understandability/Clarity (output)										x				x	x	x			x	x		x		x					x	x
Completeness (output)					x		x		x	x						x				x	x	x							x	x
Timing				x	x					x						x			x	x		x				x			x	
Conciseness (output)										x				x		x			x	x		x					x		x	
Data quality (input)					x				x		x				x	x	x											x		
Information overload	x				x											x			x	x									x	x
Feedback				x	x				x	x									x	x									x	
Data availability (input)					x						x					x	x			x									x	
Guidance				x	x					x						x				x									x	
Guideline conformity					x					x	x					x													x	

(continued)

Influencing factors: Mapping to included reviews (continued)

**Influencing factor**	Abell et al. (2023)	Adepoju et al. (2017)	Araujo et al. (2020)	Chan et al. (2023)	Chen et al. (2022)	Chima et al. (2019)	Devaraj et al. (2014)	Dingel et al. (2024)	Fernández-Barceló et al. (2023)	Gao et al. (2022)	Hendriks et al. (2024)	Higgins et al. (2023)	Hua et al. (2024)	Jun et al. (2018)	Kamel Rahimi et al. (2024)	Kilsdonk et al. (2017)	Kouri et al. (2022)	Liu et al. (2021)	Marcilly et al. (2015)	Meunier et al. (2023)	Miller et al. (2015)	Moxey et al. (2010)	Olouch et al. (2012)	Perivolaris et al. (2024)	Shakibaei Bonakdeh et al. (2024)	Tricco et al. (2023)	van Dort et al. (2019)	Wang et al. (2023)	Westerbeek et al. (2021)	Wohlgemut et al. (2023)
Involvement of stakeholders in development and design / Iterative process									x	x		x			x	x				x					x				x	
Workflow integration/compatibility	x		x	x	x		x		x	x	x		x	x		x				x	x			x	x	x	x	x	x	x
Workload/Effort	x	x		x	x	x			x	x			x			x		x		x	x					x		x	x	x
Time expenditure/Efficiency	x			x	x					x	x					x				x				x		x	x	x	x	x
Quality of care	x				x		x								x	x				x	x	x		x			x	x	x	x
Communication	x			x	x				x	x										x				x		x		x	x	x
Interdisciplinarity/Collaboration	x		x	x						x					x	x				x									x	x
Lack of time (cannot use)			x				x		x	x	x					x				x		x							x	
Clinical error							x									x								x			x		x	
Change in practice										x						x								x					x	
Productivity							x													x				x						
Effectiveness																				x				x					x	
Organisational readiness (Infrastructure and Resources)	x	x	x	x	x			x		x			x	x	x	x		x		x	x		x	x	x	x		x	x	x
User/customer support		x			x		x			x						x				x	x					x			x	
Staffing				x						x					x	x				x				x					x	
Technical difficulties	x									x	x					x				x	x								x	
Already existing solution																				x									x	
Organisation support / culture / leadership	x	x	x	x			x								x	x				x		x				x	x		x	

(continued)

Influencing factors: Mapping to included reviews (continued)

**Influencing factor**	Abell et al. (2023)	Adepoju et al. (2017)	Araujo et al. (2020)	Chan et al. (2023)	Chen et al. (2022)	Chima et al. (2019)	Devaraj et al. (2014)	Dingel et al. (2024)	Fernández-Barceló et al. (2023)	Gao et al. (2022)	Hendriks et al. (2024)	Higgins et al. (2023)	Hua et al. (2024)	Jun et al. (2018)	Kamel Rahimi et al. (2024)	Kilsdonk et al. (2017)	Kouri et al. (2022)	Liu et al. (2021)	Marcilly et al. (2015)	Meunier et al. (2023)	Miller et al. (2015)	Moxey et al. (2010)	Olouch et al. (2012)	Perivolaris et al. (2024)	Shakibaei Bonakdeh et al. (2024)	Tricco et al. (2023)	van Dort et al. (2019)	Wang et al. (2023)	Westerbeek et al. (2021)	Wohlgemut et al. (2023)
Funding/financing/cost	x	x		x			x			x					x	x				x		x			x	x			x	
(Financial) incentives		x			x		x		x	x						x				x		x			x				x	
Governance	x				x										x	x													x	
Organisational goals/expectations	x																								x					
Vendor considerations	x																								x					
Adopter-vendor communication																				x					x					
Procurement experience																									x					
Engagement (strategy)	x		x	x	x											x									x	x	x			
Implementation planning	x								x	x						x		x							x				x	
Local champions	x		x		x											x				x										
Implementation team				x	x																									
Responsible person					x																									
Addressing other barriers to the behaviour change targeted by the CDSS																	x													
Target condition				x										x								x							x	
Ward/department/setting	x			x										x															x	
Target population				x																										
Priorities in the healthcare area				x																										
Relationship to the patient / communication	x				x					x						x				x		x		x				x	x	
Patient's preferences/attitude/understanding					x						x									x		x							x	x

(continued)

Influencing factors: Mapping to included reviews (continued)

**Influencing factor**	Abell et al. (2023)	Adepoju et al. (2017)	Araujo et al. (2020)	Chan et al. (2023)	Chen et al. (2022)	Chima et al. (2019)	Devaraj et al. (2014)	Dingel et al. (2024)	Fernández-Barceló et al. (2023)	Gao et al. (2022)	Hendriks et al. (2024)	Higgins et al. (2023)	Hua et al. (2024)	Jun et al. (2018)	Kamel Rahimi et al. (2024)	Kilsdonk et al. (2017)	Kouri et al. (2022)	Liu et al. (2021)	Marcilly et al. (2015)	Meunier et al. (2023)	Miller et al. (2015)	Moxey et al. (2010)	Olouch et al. (2012)	Perivolaris et al. (2024)	Shakibaei Bonakdeh et al. (2024)	Tricco et al. (2023)	van Dort et al. (2019)	Wang et al. (2023)	Westerbeek et al. (2021)	Wohlgemut et al. (2023)
Patient engagement																				x				x						
Patient’s trust											x													x						
Governmental initiatives																				x					x					
Competition				x																										
National/regional projects																				x										
Progression towards digitalisation																									x					

x = Barrier, **x = Facilitator**, **x = Both**, **x = Influencing factor without direction of effect**; Abbreviations: CDSS computerised clinical decision support systems
