# Supplementary material for: Barriers and facilitators to the implementation and adoption of computerised clinical decision support systems: an overview of reviews
Source: Syst Rev. 2026 May 13;15:166. doi: 10.1186/s13643-026-03200-2 (PMC13173960; doi:10.1186/s13643-026-03200-2)
Supplement: Supplementary file 6 — Additional file 6. Influencing factors: Manifestations as barriers and facilitators. [file 13643_2026_3200_MOESM6_ESM.pdf]

Human factors: Manifestations as barriers and facilitators

**Influencing factor**	**Manifestations as a barrier**	**Manifestations as a facilitator**
Usefulness and perceived benefits	Lack of usefulness and benefits	• Usefulness/helpfulness and perceived benefits<br>• Presence of specific functionalities and features (classified as facilitators)
Trust	• Lack of trust<br>• Various concerns regarding CDSS	Trust
Attitude/Preferences/Values	• Negative attitude towards CDSS<br>• Opposing preferences<br>• Violation of value	• Positive attitude towards CDSS<br>• Matching values and preferences
Perceived autonomy of action	Limited/reduced autonomy of action	High/enhanced autonomy of action
Resistance/Readiness to use/technology/change	Resistance to use/technology/change	Readiness/willingness to use/technology/change
Agreement with recommendations	Disagreement with recommendations	Agreement with recommendations
Beliefs about impact on professional competence	• De-skilling<br>• Questioned/undermined competence<br>• Cookbook medicine<br>• Over-dependence on technology	–
Need for support/CDSS	No need for CDSS	Need for CDSS
Stress	Increased stress	Less stress
Role/responsibility	Negative perceived impact on or unclear role/responsibility	Positive perceived impact on role/responsibility
Prior experiences	Prior negative experience with CDSS use	Prior positive experience with CDSS use
Frustration	Frustration with CDSS use	–
Confidence in using the system	Low confidence in using the system	High confidence in using the system
Satisfaction	No satisfaction	Satisfaction
Voluntariness	Use not voluntary but management opposed	Voluntary use
Job security	Lack of job security	Job security
Perceived risk	High perceived potential of negative consequences associated with technology use	No perceived potential of negative consequences associated with technology use

(continued)

Human factors: Manifestations as barriers and facilitators (continued)

**Influencing factor**	**Manifestations as a barrier**	**Manifestations as a facilitator**
Alert fatigue	• Alert fatigue<br>• Too many alerts	Low alert burden
Information overload	Information overload	Appropriate amount of information
Medicolegal concerns	Medicolegal concerns	• Legal protection<br>• Medicolegal safety
Security/privacy concerns	Security/privacy/data loss concerns	Data security/privacy
Knowledge of the system	• Lack of knowledge about system<br>• Low literacy	• Education on the system<br>• Receiving and communication of updates on the system
Awareness of the system	Lack of awareness of the system	–
Clarity of the purpose of the system	Lack of understanding/awareness of the system's purpose and capabilities	• Clear purpose<br>• Perceived as support tool
Training	• Lack of training<br>• Poor training<br>• Delay between training and implementation	(Sufficient) training
Technical skills	Lack of technical/computer skills	Technical skills
Education/improvement	No potential of education and improvement	• CDSS enhances expertise<br>• Used as education/teaching tool
Professional experience/expertise	• Not useful/adequate for experienced professionals ("experience supersedes guidelines", "experts do not need decision support")<br>• CDSSs may create more confusion among young physicians	Helpful for less experienced staff
Familiarity with CDSS	Lack of familiarity with CDSS	Familiarity with CDSS
Social acceptance (use/opinion by others)	Lack of social acceptance	• Perception that important others believe that the CDSS should be used<br>• Perception that the use of CDSS is expected<br>• Perceived use by others

(continued)

Human factors: Manifestations as barriers and facilitators (continued)

Influencing factor	Manifestations as a barrier	Manifestations as a facilitator
Discrete accessibility	–	Discrete accessibility
Conflicts	Conflicts in teams due to CDSS use	–
Age	Old age	Young age

Abbreviations: CDSS computerised clinical decision support systems

Technology-related factors: Manifestations as barriers and facilitators

Influencing factor	Manifestations as a barrier	Manifestations as a facilitator
Usability / ease of use	Usability issues	• Ease of use<br>• Absence of usability flaws
Design (UX)	Poor user interface design / user experience	Good user interface design / user experience
Interoperability, Integration and information standards	Lack of interoperability and integration with other systems	• Interoperability and integration with other systems<br>• Information standards
Flexibility/Adaptability	• Lack of flexibility/adaptability/customisation<br>• Presence of work around pathways	Flexibility/adaptability/customisation of system
Decision model complexity / Transferability	Insufficient decision model complexity:<br>• Not sensitive to complexity of patients or practice<br>• Not transferable to a different context<br>• Limited number of conditions<br>• Lack of learning capacities<br>• Not adequate for some subpopulations<br>• Some patient cases are not supported<br>• CDSS is not considering all context factors	• Adaptation to suit the context<br>• Enlarging the coverage of CDSSs<br>• Continuous learning capacity
Transparency/Explainability	Lack of transparency/explainability	Transparency/explainability
Correct functionality (Technical reliability)	• Incorrect functionality<br>• Lacking technical reliability<br>• Inconsistent output	• Correct functionality<br>• Technically reliable
Product standardisation	Lack of standardisation for CDSS technology	Standardisation (of product, tool, data collection, assessment, health information technology)
Device (size and shape)	–	• Smaller devices<br>• CDSS use on tablet<br>• General size and shape
Active vs. passive CDSS	• Passive CDSS<br>• Interruptive alerts	• Minimally or non-interruptive alterts<br>• Pull functions<br>• Passive CDSS<br>• Active CDSS

(continued)

Technology-related factors: Manifestations as barriers and facilitators (continued)

Influencing factor	Manifestations as a barrier	Manifestations as a facilitator
Novelty of technology	–	Novelty of the system
Performance assessment	Insufficient performance assessment	Robust performance monitoring and evaluation
Decision model validity/reliability of evidence base	• Decision model not validated<br>• Unreliable evidence base / information<br>• Lacking evidence base<br>• Outdated information	• Decision model validated<br>• Reliable evidence base / information / recommendations
Correctness / accuracy (output)	• Inadequate performance<br>• Incorrect output<br>• Lack of accuracy in CDSS recommendations<br>• Lacking quality of information	• Accurate recommendations/data<br>• High specificity and sensitivity of information
Relevance (output)	• Lacking relevance of output in practice<br>• Irrelevant information	Relevance of output in practice
Understandability/Clarity (output)	Lacking understandability/clarity of output	Understandability/clarity of output
Completeness (output)	• Incompleteness of recommendation (missing information, missing contextualisation)<br>• Lacking provision of extra information (metrics of use and effectiveness of CDSS, link to guidelines / supporting information, education sheet)	• Completeness of CDSS<br>• Provision of extra information (historical data, relevant patient info, references, risk calculation, care plan, background information, explanations for recommendations, linked medication order, link to guidelines, alternative treatments, costs)
Timing	Wrong timing (output too late / output not displayed at the right time)	Right timing (close to decision moment)
Conciseness (output)	Output not concise	Concise output
Data quality (input)	• Poor input data quality<br>• Reliance on that data	• High input data quality<br>• Evaluation of input data quality
Feedback	No feedback to users	Feedback to users
Data availability (input)	Lacking data availability	• Data availability<br>• Evaluation of data availability

(continued)

Technology-related factors: Manifestations as barriers and facilitators (continued)

**Influencing factor**	**Manifestations as a barrier**	**Manifestations as a facilitator**
Guidance	Lacking guidance on workflow	Guidance on actions (hands-on information)
Guideline conformity	Conflicting guidelines	–
Involvement of stakeholders in development and design / Iterative process	–	• Involvement of stakeholders in development process<br>• Iterative development process

Abbreviations: CDSS computerised clinical decision support systems

Contextual factors: Manifestations as barriers and facilitators

**Influencing factor**	**Manifestations as a barrier**	**Manifestations as a facilitator**
Workflow integration/ compatibility	• Lack of workflow integration and compatibility<br>• Workflow interruption	• Workflow integration and compatibility<br>• Improved workflow<br>• Not interruptive to workflow
Workload/Effort	High/increased effort/workload	• No increase / decrease in workload<br>• Low effort to use CDSS
(Local) Feasibility/Applicability	• Use not feasible (due to practical circumstances, loco-regional circumstances/constraints, patients cannot afford recommended treatment)<br>• Lack of applicability	• Easy to implement<br>• Applicability<br>• Taking into account the loco-regional characteristics
Time expenditure/Efficiency	• High/increased time expenditure<br>• Reduced efficiency	• Low reduced time expenditure<br>• High/increased efficiency
Quality of care	Reduced quality of care	• Improved quality of care<br>• Potential for improved quality of care
Communication	• Interference with communication<br>• Lack of communication	• Improved / incresed communication<br>• Communication between CDSS stakeholders
Interdisciplinarity / Collaboration	• Minimisation of group situational awareness<br>• Diversity of medical practices involved complicates the development of shared clinical content	Interdisciplinarity/Collaboration
Lack of time (cannot use)	Lack of time	–
Clinical error	Potential for new sources of clinical error (prefilled information, data entry errors)	Potential to decrease clinical error
Change in practice	Change in practice disliked	Heavy users adapt behaviour
Productivity	Loss of productivity	Improved productivity
Effectiveness	No impact on decision making	Effective support

(continued)

Contextual factors: Manifestations as barriers and facilitators (continued)

**Influencing factor**	**Manifestations as a barrier**	**Manifestations as a facilitator**
Organisational readiness (Infrastructure and Resources)	• Lack of organisational readiness (infrastructure and resources)<br>• Increased resources	High organisational readiness (infrastructure and resources)
User/customer support	Lack of user/customer support	User/customer support
Staffing	• Not adequate staffing<br>• High turnover	Sufficient and well-suited staffing
Technical difficulties	Technical difficulties	Low or no technical dependencies
Already existing solution	Already existing solution	–
Organisation support/ culture/ leadership	• Lack of organisation/leadership support<br>• Workplace culture	• Strong organisation support/culture/leadership<br>• Endorsement by management / professional body
Funding/financing/cost	• Uncertain/lacking funding/financial support<br>• Financial constraints<br>• High costs	• Adequate financial support / funding<br>• Potential cost savings<br>• Cost-effectiveness
(Financial) incentives	• Lack of incentives (e.g. financial)<br>• Risk of wrong incentives (solely financial or to improve rankings)	Incentives (e.g. financial)
Governance	Governance	–
Organisational goals / expectations	Lacking alignment of CDSS with organisational goals and expectations	Alignment of CDSS with organisational goals and expectations
Vendor considerations	• Vendor considerations<br>• Market share and policy of vendors<br>• Market share and policy of vendors	
Adopter-vendor communication	Knowledge imbalance between hospitals and vendors regarding the capabilities and requirements of CDSS technology	Known mediator between the user and the vendor<br>communication path between adopter and vendor
Procurement experience	Lack of experience with CDSS procurement procedure	–

(continued)

Contextual factors: Manifestations as barriers and facilitators (continued)

Influencing factor	Manifestations as a barrier	Manifestations as a facilitator
Engagement (strategy)	Lack of engagement in planning and implementation (clinician, user)	(Early) involvement/engagement in planning and implementation (clinician, adopter, user, administration, management)
Implementation planning	Lack of or dissatisfying strategic implementation planning	Strategic implementation planning
Local champions	Lack of local/internal champions	Local/internal champions
Implementation team	–	• Dedicated implementation teams<br>• Responsible person<br>• Team work
	–	Responsible person
Addressing other barriers to the behaviour change targeted by the CDSS	–	Identifying and addressing other barriers to the behaviour change targeted by the CDSS
Target condition	Clinical condition (e.g. the target condition is very rare)	Clinical condition (e.g. treating patients with less severe complaints or polypharmacy, useful for patients with unknown or unfamiliar infection sources, chronic conditions, psychiatric conditions)
Ward/department/setting	• Lack of contextual fit of CDSS with setting<br>• Inappropriate setting for CDSS	Contextual fit of CDSS with setting
Target population	–	Fit of the target population
Priorities in the healthcare area	–	• Existing priorities and efforts in the target area<br>• Tool contributes to solving a priority health care area/problem in the region or country
Relationship to the patient / communication	(Fear of) negative impact on clinician-patient relationship/communication	• Positive impact on clinician-patient relationship<br>• enhanced communication
Patient's preferences/attitude/understanding	• Patient's own preferences / health literacy / lack of understanding<br>• Patient disagreement with CDSS recommendations	Patient satisfaction / perception / acceptance of CDSS

(continued)

Contextual factors: Manifestations as barriers and facilitators (continued)

**Influencing factor**	**Manifestations as a barrier**	**Manifestations as a facilitator**
Patient engagement	–	CDSS helps to increase patient engagement
Patient’s trust	–	Patients’ trust in doctors
Governmental initiatives	–	Governmental support
Competition	–	Competition due to implementation at other sites
National/regional projects	–	Being part of a national project
Progression towards digitalisation	–	Progression towards digitalisation

Abbreviations: CDSS computerised clinical decision support systems
