# Supplementary material for: Barriers and facilitators to the implementation and adoption of computerised clinical decision support systems: an overview of reviews
Source: Syst Rev. 2026 May 13;15:166. doi: 10.1186/s13643-026-03200-2 (PMC13173960; doi:10.1186/s13643-026-03200-2)
Supplement: Supplementary file 7 — Additional file 7. Frequency of occurrence of influential factors (subgroup: low risk of bias). [file 13643_2026_3200_MOESM7_ESM.pdf]

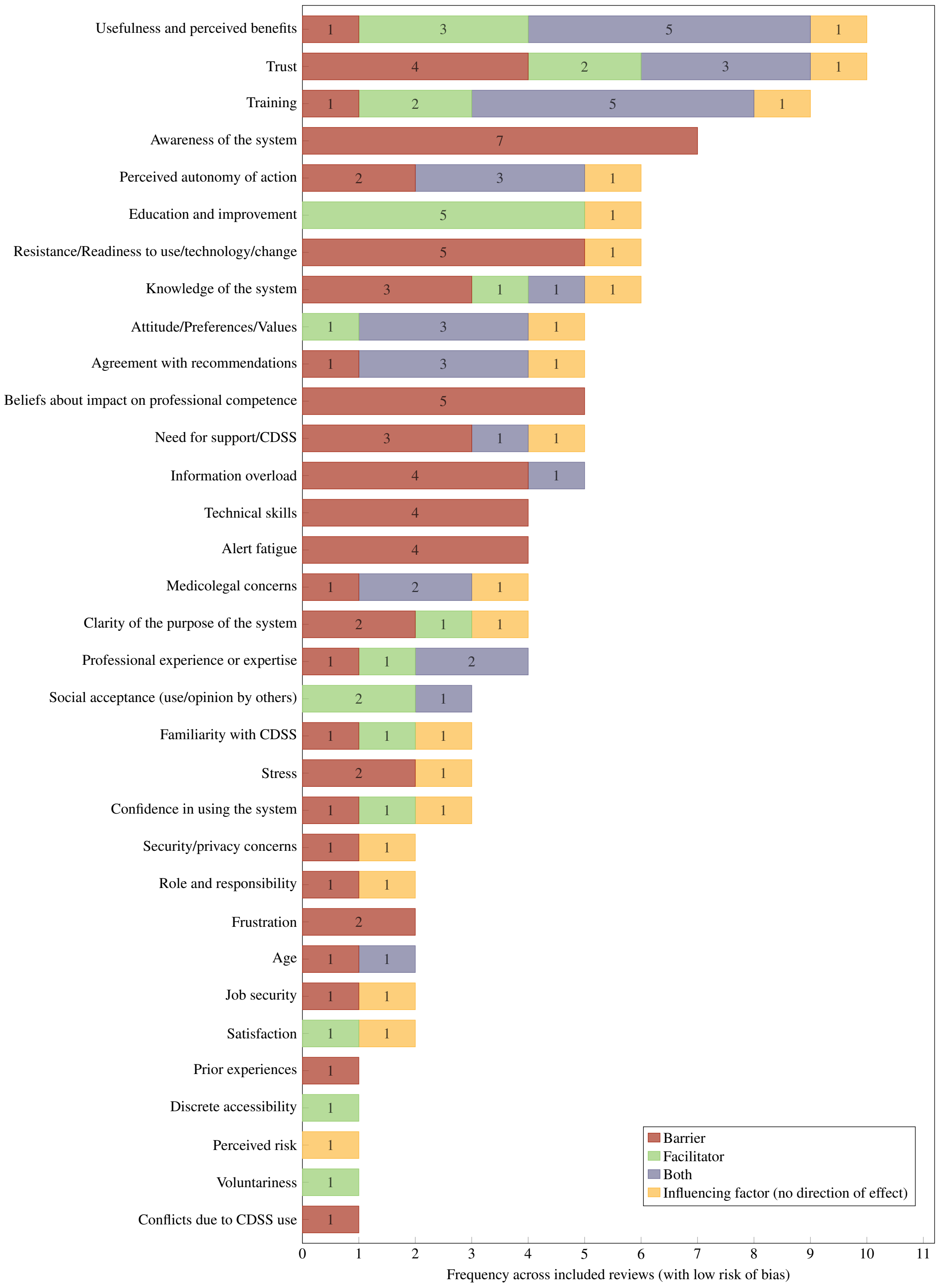

Human factors: Frequency across included reviews with low risk of bias (n=14) by direction of effect. CDSS: computerised clinical decision support system

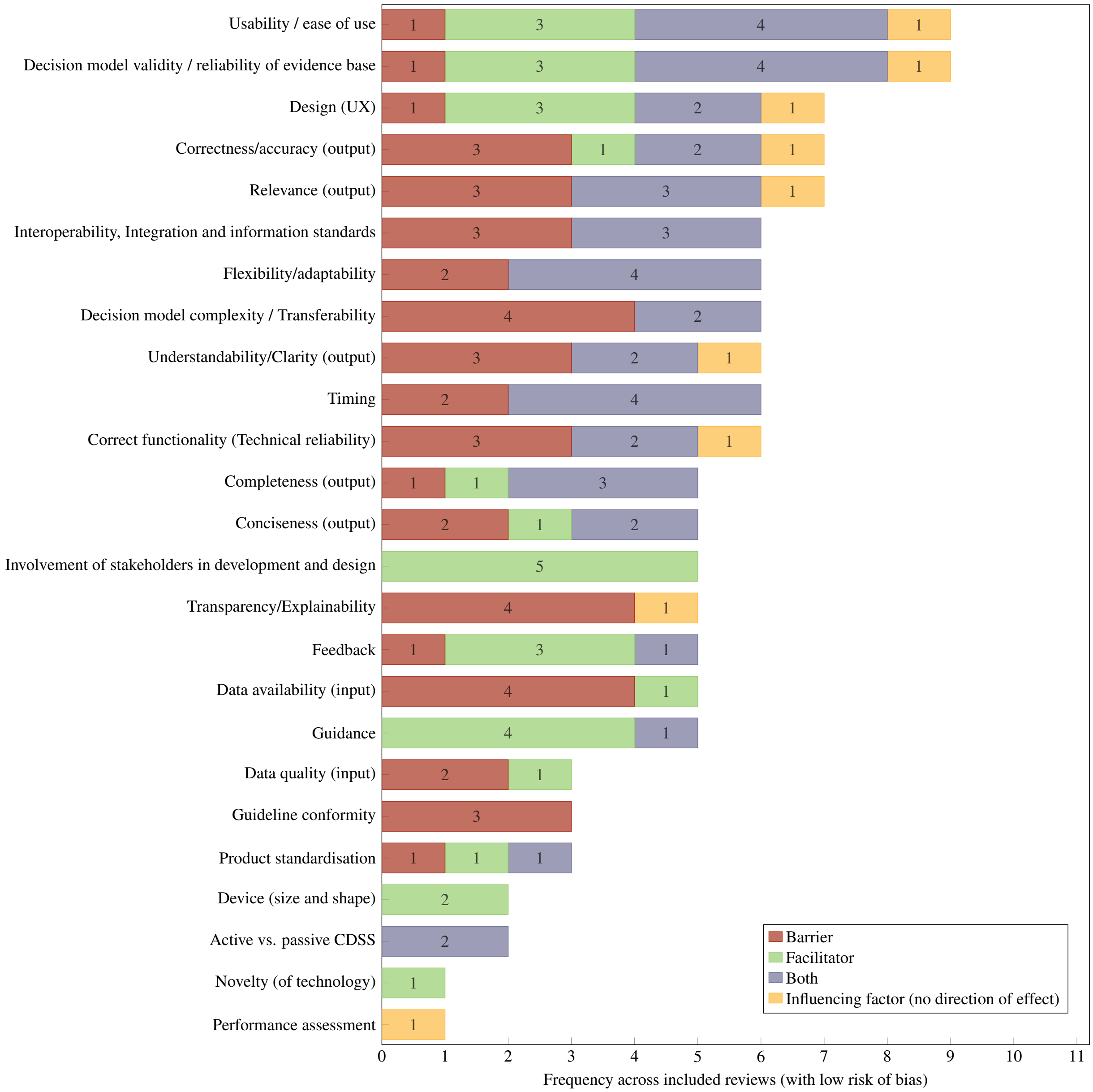

Technology-related factors: Frequency across included reviews with low risk of bias (n=14) by direction of effect. CDSS: computerised clinical decision support system, UX user experience

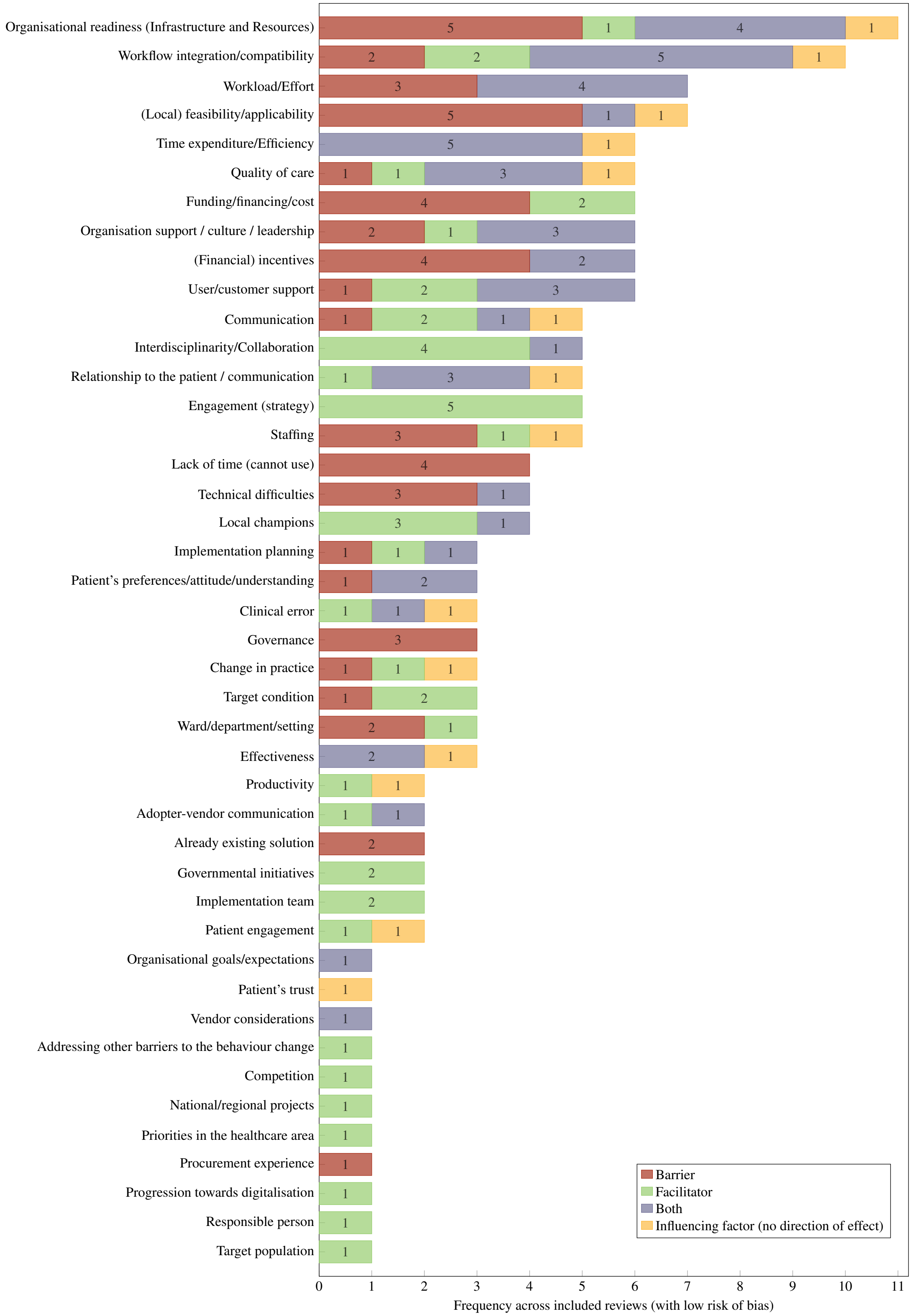

Contextual factors: Frequency across included reviews with low risk of bias (n=14) by direction of effect. CDSS: computerised clinical decision support system
